# Comparative effectiveness of darbepoetin vs other agents in chronic kidney disease-related anemia: a systematic review and network meta-analysis

**DOI:** 10.1186/s12882-025-04557-7

**Published:** 2025-11-17

**Authors:** Muhammad Faique Hassan, Muhammad Shaheer Bin Faheem, Shamikha Cheema, Fathimathul Henna, Saman Javid, M. Rafiqul Islam, Insha Habib, Muhammad Usman, Yousif Hameed Kurmasha, Sumaya Samadi, Yaqub Nadeem Mohammad

**Affiliations:** 1 King Edward Medical University, Lahore, Pakistan; 2 Karachi Institute of Medical Sciences, KIMS, Karachi, Pakistan; 3 College of Medicine, Dubai Medical College for Girls, Dubai, UAE; 4 CMH Kharian Medical College, Kharian Cantt, Pakistan; 5 Shaheed Suhrawardy Medical College Hospital, Dhaka, Bangladesh; 6 Al Nafees Medical College, Islamabad, Pakistan; 7 Dow University of Health Sciences, Karachi, Sindh Pakistan; 8 College of Medicine, University of Kufa, Najaf, Iraq; 9 Kabul University of Medical Sciences “Abu Ali Ibn Sina”, Kabul, Afghanistan; 10 Ascension Borgess Hospital, Kalamazoo, Michigan USA

**Keywords:** Chronic kidney disease, Erythropoietin-stimulating agents, Darbepoetin alfa, Network meta-analysis

## Abstract

**Introduction:**

In the advanced stages of chronic kidney disease (CKD), anemia impacts 78.9% to 96.5% of patients. Darbepoetin is utilized for the treatment of anemia associated with chronic kidney disease (CKD). The function of darbepoetin alfa can be clarified by comparing it with other agents. A comprehensive review and network meta-analysis were conducted to assess the safety and efficacy of Darbepoetin alfa compared to other agents in the treatment of anemia associated with chronic kidney disease (CKD).

**Methods:**

A systematic review and network meta-analysis were conducted adhering to PRISMA-NMA guidelines. A systematic search was performed in databases Medline, Embase, and Scopus from inception to November 24, 2024, without language limitations. Randomized controlled trials assessing erythropoietin-stimulating agents (ESAs) or placebo in adults with chronic kidney disease (CKD)-related anemia were included. Data extraction and risk of bias assessment (RoB 2.0) were performed separately. Bayesian network meta-analysis using random-effects models was carried out using BUGSnet in R. Treatment effects were measured as odds ratios (ORs) or mean differences (MDs) with 95% credible intervals. Heterogeneity, inconsistency, and publication bias were evaluated, and certainty of evidence was assessed using the GRADE approach.

**Results:**

In this network meta-analysis of 21 randomized trials involving over 4,000 CKD patients with anemia, Methoxy polyethylene glycol-epoetin beta showed the greatest hemoglobin increase, while Molidustat demonstrated the best cardiovascular and thrombotic safety despite lower efficacy. Daprodustat provided moderate hemoglobin improvement with a safety profile comparable to darbepoetin, showing no significant differences in mortality or cardiovascular events. Transferrin saturation, hypertension, and diabetes-related adverse events did not differ significantly across treatments. Roxadustat was associated with a higher incidence of gastrointestinal adverse events, particularly diarrhea.

**Conclusion:**

Daprodustat and roxadustat indicated equivalent or higher efficacy when compared to darbepoetin in elevating hemoglobin levels in CKD-related anemia, with daprodustat demonstrating a good safety profile. While conventional ESAs remain beneficial, HIF-PHIs offer promising oral alternatives with possible benefits in cardiovascular safety and decreased hypertension risk. Further head-to-head trials are necessary to confirm these findings and guide individualized treatment strategies.

**Clinical trial number:**

Not applicable.

**Supplementary information:**

The online version contains supplementary material available at 10.1186/s12882-025-04557-7.

## Introduction

Chronic kidney disease (CKD) is a major global health burden affecting 13.4% (11.7–15.1%) of the world’s population and highly associated with comorbidities such as diabetes and cardiovascular disease [1–5]. As CKD is defined as GFR < 60 mL/min/1.73 m2 and evidence of kidney deterioration markers like proteinuria, worsening of this condition also results in a clear decline in the functioning capacities of kidneys. Taking into account all these findings, the need for exclusive inquiry becomes imminent.

Anemia in patients with chronic kidney disease is an extremely recurrent complication with almost 78.9–96.5% of patients suffering from it in later stages. Quality of life and physical capacity are remarkably affected within this population. Moreover, anemia of CKD is also associated with high cardiovascular comorbidities. Interventions targeting underlying causes are used for treating this disorder. Renal anemia [6] is the result of a decrease in EPO production cells which is regulated by hypoxia-inducible factor.

Erythropoietin-stimulating agents (ESAs) are used for renal anemia because these increase hematocrit levels, diminish the need for RBC transfusions, and help alleviate the symptoms of renal anemia [7, 8]. Limitations for the use of ESAs include frequent dosing and no benefit in anemia for CV patients as documented by clinical trials like CHOIR and TREAT. An ongoing trial investigating a new agent (Darbepoetin alfa) for anemia of CKD, titled “A Phase 3 Study of Efepoetin Alfa for Treatment of Anemia in Patients With Chronic Kidney Disease on Dialysis” (Clinicaltrial.gov Identifier: NCT06466785) is in progress and may provide helpful evidence about its efficacy and safety.

Darbepoetin alfa (DPO) commonly known as Aranesp is also an erythropoietin stimulating agent but it has 2 extra N-liked carbohydrate addition sites which make it more favorable than other EPOs. Advantages of shifting to this new EPO include a long half-life, reduction in dosing [9], and its usefulness. So, its effectiveness must be explored to mitigate anemia of CKD. Prior meta-analyses have demonstrated the role of darbepoetin alfa in comparison with other agents like epoetin alfa [10] and vadadustat [11]. To analyze the fundamental role of darbepoetin alfa, its comprehensible comparative comparison with other agents is critically important. Hence, keeping in mind the need for remedy for anemic patients of CKD, we conducted a systematic review and network meta-analysis on the safety and efficacy of darbepoetin alfa in patients with CKD. This analysis will surely help in understanding the etiology behind the disorder and the best management option for anemic patients by correlating several interventions.

## Methods

We conducted a systematic review and meta-analysis by the approach recommended by the Preferred Reporting Items for Systematic Reviews and Meta-analysis (PRISMA) incorporating network meta-analysis (PRISMA-NMA) statement for conducting a meta-analysis of intervention studies [12].

### Data sources and searches

We conducted a systematic review of the literature by searching Medline, Embase, and Scopus (from inception till 24th November 2024) without any language or article type restriction. We used all known spelling of search terms relevant to this study as well as the Medical Subject Headings (MeSH) including the terms: “anemia”, “chronic kidney disease”, “CKD”, “renal failure”, “darbepoetin”, “Daprodustat”, “epoetin” etc. A complete list of search terms and search strategies is given in Supplementary Table S1. To find any more pertinent studies, the reference lists of the indicated trials and review articles were manually examined. We also looked for randomized trials that were recorded as finished but not yet published on the ClinicalTrials.gov website. If any specific information was needed, we contacted the authors to request the data.

### Study selection

Our primary aim was to include all randomized controlled trials (RCTs) studying erythropoietin stimulating agents and Placebo for anemia in adult patients with chronic kidney disease. Hence, only RCTs comparing any erythropoietin stimulating agent with another ESA or placebo were studied in patients with anemia and CKD, with CKD being defined as glomerular filtration rate [GFR], 60 mL/min/1.73 m2, or elevated serum creatinine level or albuminuria with albumin excretion. 30 mg/d, or abnormalities detected by histology or dialysis, and anemia as a hemoglobin concentration < 11.0 g/dL, adequate iron stores (as determined by serum ferritin > 100 ug/L), serum vitamin B 12 and folate levels above the lower limit of the normal range. Any other study design studying drugs other than ESAs was excluded. Patients with any other co-morbid or hematologic disorders causing anemia were also excluded. The literature was searched and individual titles and articles were screened for inclusion criteria by 2 investigators (SC, MFH) independently and all conflicts were resolved by discussion.

### Data extraction and quality assessment

Two authors extracted the data from the included studies using standardized data extraction forms created in Microsoft Excel, which were checked by another author before analysis. Baseline patient characteristics, medication dosage, length of follow-up, blood pressure change, outcome events, and adverse events were among the data extracted. These data were taken from either studies that only included individuals with chronic renal disease or subgroups of previous trials that provided baseline data for the population with CKD. If the required data was not provided in text or tables, we used PlotDigitzer (https://plotdigitizer.com) to extract the relevant data from the figures. In case of missing data authors were contacted.

The methodological quality of each included study was evaluated with the Cochrane Risk of Bias 2.013 assessment technique and reported in RevMan5 (3). Each study’s internal validity (bias) was defined by five domains: (1) randomization, (2) deviations from intended interventions, (3) missing outcome data, (4) outcome measurement, and (5) reported result selection. These domains are then used to calculate the overall risk of bias, which is classified as low, medium, or high.

### Data synthesis and analyses

R (version 2.13.1; R Foundation for Statistical Computing) was used to perform network meta-analysis with a random-effects mixed-treatment comparisons model for multi-arm trials within the Bayesian framework on the effects of ESAs on patients suffering from anemia and concomitant CKD. We assumed a binomial distribution for the outcome. Nodes of all the different ESAs and placebo were included in the network analysis and a network plot was generated. The relative probabilities of events in the arms of a study can be parameterized in terms of the logarithm of the odds ratio (OR), and final pooled ORs and their 95% credible intervals were used to compare treatment effects for binary outcomes whereas a change in different continuous parameters (like hemoglobin, etc) in the arms of a study pre and post-treatment can be parameterized in terms of the Mean Difference (MD), and final pooled MDs and their 95% credible intervals were used to compare treatment effects for each outcome. Results are displayed as forest plots compared to placebo, with effect estimates shown as posterior medians of OR or MD with 95% CIs. The link function for binary outcome data was logit, and the likelihood distribution was assumed to be binomial; for continuous data, the link function was identity, and the likelihood distribution was assumed to be normal. Surface under the cumulative ranking (SUCRA) plots and tables are used to display treatment ranking; the SUCRA value, which ranges from 0% to 100%, indicates the likelihood that the treatment is ranked first or among the top ranks. Additionally, results are displayed in league heat plots that show all comparisons within a particular outcome.

## Results

### Study selection and characteristics

#### Literature search

A systematic literature search was conducted across four databases—Pubmed (*n* = 1341), Embase (*n* = 1020), Scopus (*n* = 1079), and ClinicalTrials.gov (*n* = 20)—yielding a total of 3460 records. After removing 560 duplicate entries, 2900 unique records were screened based on title and abstract. Of these, 2835 were excluded for not meeting the eligibility criteria. The full texts of the remaining 65 articles were assessed in detail, and 44 were excluded due to reasons such as irrelevant population, incomplete outcome data, or other eligibility violations.

**Fig. 1:**
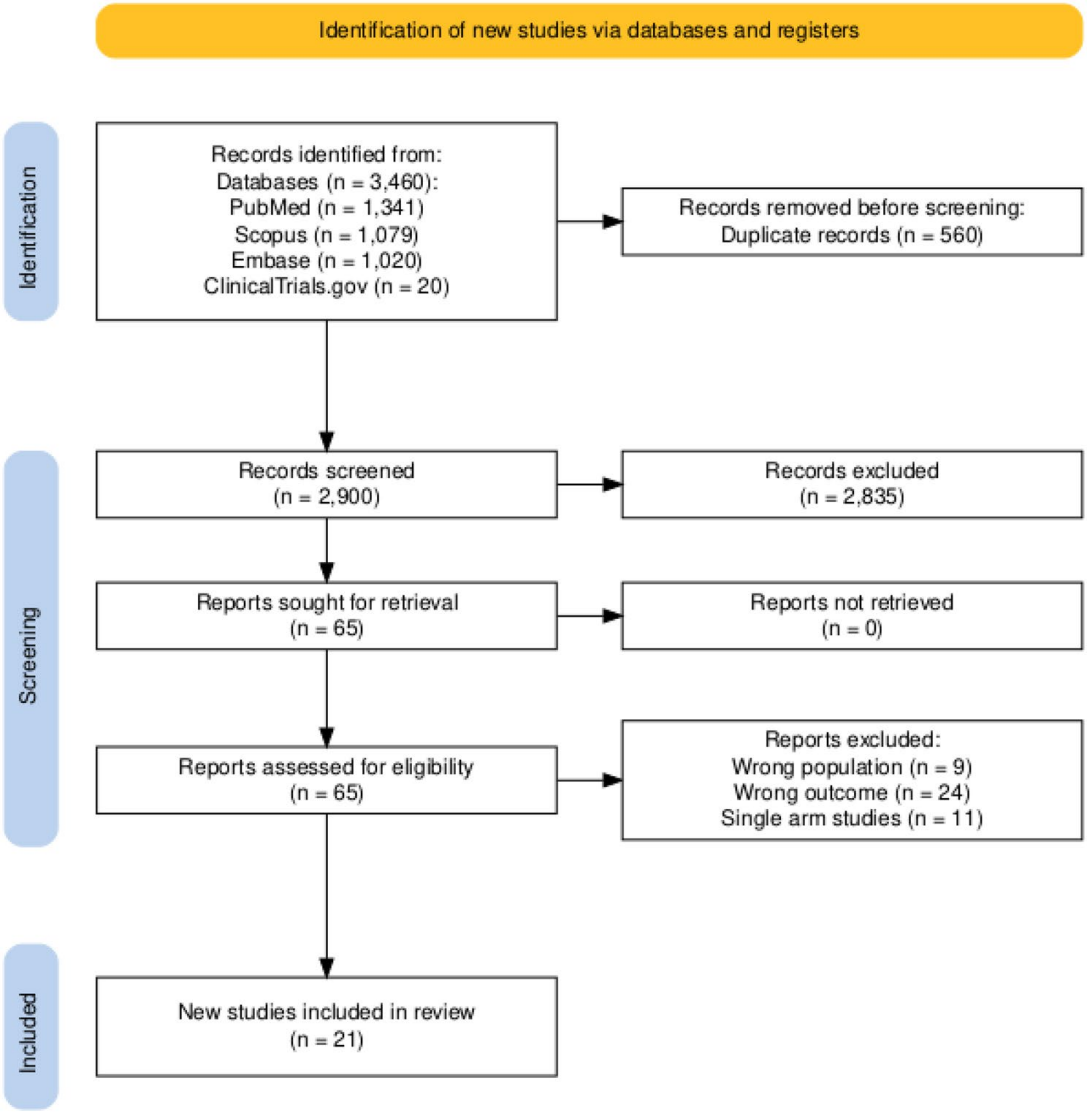
PRISMA flowchart

In this network meta-analysis, a total of 21 randomized controlled trials comprising more than 4,000 patients with anemia secondary to chronic kidney disease were included. The evaluated erythropoiesis-stimulating agents (ESAs) consisted of Daprodustat, Roxadustat, Vadadustat, Epoetin alfa, Continuous Erythropoietin Receptor Activator (CERA), Molidustat, Methoxy polyethylene glycol-epoetin beta (PEG-epoetin beta), and recombinant human erythropoietin (rHuEPO). Populations in these studies consisted of patients with dialysis-dependent or non-dialysis-dependent chronic kidney disease. The baseline characteristics across studies were well balanced, with mean patient ages ranging from 45 to 70 years and similar hemoglobin levels and comorbidity profiles, thereby enhancing the reliability of the comparative analyses. The study characteristics are shown in Tables 1 and 2. The details of the search strategy are shown in the Fig. 1.

**Table 1:** Study design characteristics

First Author, Year					Time of realization		Duration of follow-up (time of points of outcome report)	Criteria	
	Intervention	Place of study	Study Design/Phase	Total sample size	Beginning	End		Inclusion criteria (Define Patients emergent state, Disease State)	Exclusion criteria
Singh et al.	Daprodustat	Across 14 countries	Randomized Clinical Trial	312	May-17	Sep-20	28 to 52 weeks	Between 18 and 99 years of age inclusive - Planning to start chronic dialysis within the next 6 weeks from the date of the screening visit OR have started and received dialysis (as specific below) for end-stage renal disease for a maximum of ≤ 90 days immediately prior to randomization and is not expected to stop dialysis during the duration of the trial: o Hemodialysis ≥2 times a week o Peritoneal dialysis ≥4 times a week including incremental schedule; patients on continuous ambulatory peritoneal dialysis (CAPD) and automated peritoneal dialysis (APD) are eligible - Hemoglobin concentration as measured by HemoCue (ranges inclusive) of 8–10.5 g/dL (5–6.5 mmol/L; 80–105 g/L) at screening and 8–11.0 g/dL (5–6.8 mmol/L; 80–110 g/L) at randomization - Informed consent at screening	- Planned living-related or living-unrelated kidney transplant during the study - Ferritin ≤100 ng/mL (≤100 µg/L) as screening or after IV iron supplementation - Transferrin saturation ≤20% at screening or after IV iron supplementation - Vitamin B12 below the lower limit of the reference range at screening or after vitamin B12 supplementation - Folate < 2.0 ng/mL ( < 4.5 nmol/L) at screening - History of bone marrow aplasia or pure red cell aplasia - Untreated pernicious anemia, thalassemia major, sickle cell disease, or myelodysplastic syndrome - Evidence of actively bleeding gastric, duodenal, or esophageal ulcer disease OR clinically significant gastrointestinal bleeding ≤10 weeks prior to screening through to randomization (Day 1) - Use of any ESA treatment within 8 weeks prior to screening except for limited use as part of dialysis initiation o Limited use defined as < 6 weeks of short-acting ESA (rhEPO or biosimilars; maximum of 20,000 U total) or long-acting ESA (darbepoetin alfa [maximum of 100 µg total] or methoxy polyethylene glycol-epoetin beta [maximum of 125 µg total]) received before or after starting dialysis - Myocardial infarction or acute coronary syndrome ≤10 weeks prior to screening through to randomization (Day 1) - Stroke or transient ischemic attack ≤10 weeks prior to screening through to randomization (Day 1) - Chronic Class IV heart failure, as defined by the New York Heart Association (NYHA) functional classification system - Current uncontrolled hypertension as determined by the Investigator that would contraindicate the use of rhEPO - Day 1 QTcB > 500 msec, or > 530 msec in patients with bundle branch block. There is no QTc exclusion for patients with a predominantly ventricular paced rhythm - Liver disease (any one of the following): o ALT > 2x ULN (screening only) o Bilirubin > 1.5x ULN (screening only, isolated bilirubin > 1.5x ULN acceptable if bilirubin is fractionated and direct bilirubin < 35%) o Current unstable liver or biliary disease per investigator assessment (stable chronic liver disease, including asymptomatic gallstones, chronic hepatitis B or C, of Gilbert’s syndrome, are acceptable if subject otherwise meets entry criteria) - History of malignancy within 2 years prior to screening through to randomization (Day 1), or currently receiving treatment for cancer or complex kidney cyst > 3 cm o Exception is localized squamous cell or basal cell carcinoma of the skin that has been definitively treated ≥10 weeks prior to screening - History of severe allergic reactions of anaphylactic reactions or hypersensitivity to excipients in the investigational product or darbepoetin alfa - Use of strong CYP2C8 inhibitors or strong CYP2C8 inducers Use of other investigational agent or device prior to screening through to randomization (Day 1) (at screening, this exclusion applies to use of the investigational agent within 30 days or within five half-lives, whichever is longer Any prior treatment with daprodustat for treatment duration of > 30 days Females only: subject is pregnant, breastfeeding or is of reproductive potential and does not agree to follow one of the contraceptive options in the List of Highly Effective Methods for Avoiding Pregnancy listed in the study protocol Any other condition, clinical or laboratory abnormality, or examination finding that the investigator considers would put the subject at unacceptable risk, which may affect study compliance (such as intolerance to rhEPO) or prevent understanding of the aims of investigation procedures or possible consequences of the study
Liu et al.	Epoetin	China	Randomized Clinical Trial	416	Apr-13	Sep-14	21 to 28 weeks	The inclusion criteria for this study included: (1) chronic kidney failure, age ≥18 years, ≤70 years; (2) undergoing hemodialysis two or more times in 1 week, and shortacting rHuEPO preparation administration during the 12 weeks before the study; (3) Hb levels in the range of 10 g/dl to 12 g/dl before the study; (4) transferrin saturation (TSAT) ≥ 20% or serum ferritin ≥100 ng/ml during the 4 weeks before the study.	The exclusion criteria in this study were: (1) uncontrollable hypertension (diastolic blood pressure > 100 mmHg before hemodialysis); (2) congestive heart failure (New York Heart Association Class III or IV); (3) subjects having undergone surgery with massive bleeding within 12 weeks before the study; (4) malignant tumors, hematological system diseases or other hemorrhagic disorders; (5) subjects undergoing blood transfusions, or the administration of protein anabolic hormone, testosterone heptane, mepitiostane or other experimental drugs within 12 weeks before the study; (6) AST or ALT values > 3 times the upper limit of normal; (7) severe drug allergies, including epoetin alfa allergy.
Al-Ali et al.	Epoetin	Qatar	Randomized Clinical Trial	219	NA	NA	Monthly till 9 months	This study comprised of hemodialysis patients who are aged ≥18 years, have stable chronic renal anemia (with Hb range of 10–12 g/dL), and on regular hemodialysis three times per week with urea reduction ratio greater than or equal to 65% or KT/V greater than or equal to 1.2. Patients must have received hemodialysis three times weekly for ≥12 weeks before screening and during the 4-week screening/baseline period. Eligible patients must have stable Hb concentrations (stable is defined as ≤25% change ESA over 8 weeks). Recruited patients must have undergone continuous maintenance intravenous conventional epoetin alfa or beta therapy for ≥8 weeks before screening and during the screening/baseline. Patients also should have adequate iron status, defined as serum ferritin ≥100 g/L and transferrin saturation ≥20%.	1. New York Heart Association class III or IV conges-tive heart failure 2. Uncontrolled hypertension (defined as predialysis diastolic blood pressure [BP] ≥105 mmHg or sys-tolic BP ≥ 160 mmHg during the screening period) 3. Evidence of uncontrolled hyperparathyroidism (defined as parathyroid hormone level > 1000 pg/mL with no response to conventional treatment of hyperparathyroidism according to KDOQI guideline during the 12 months prior to baseline) 4. Treatment for grand mal epilepsy 5. Hematological, inflammatory, or infectious conditions that might interfere with the erythropoietin response 6. Received red blood cell transfusions within 12 weeks before screening or during the screening/baseline period 7. C-reactive protein > 30 mg/L 8. The likelihood of early withdrawal or life expectancy of < 12 months 9. Poor compliance with dialysis treatment, evidenced by more than two missed treatment monthly over the previous 3 months 10. Refuse to be involved in the study
Nissenson et al.	Epoetin	United States and Canada	Randomized Clinical Trial	504	NA	NA	21 to 28 weeks	Patient should be on hemodialysis therapy for at least 12 weeks. They also were required to be administered stable IV epoetin alfa therapy three times weekly for a minimum of 8 weeks and have a mean baseline hemoglobin concentration of 9.5 to 12.5 g/dL (95 to 125 g/L). To ensure adequate iron stores to support erythropoiesis, transferrin saturation was required to be 20% or greater.	Patients were excluded fromthe study if they had hematologic, inflammatory, infectious, or other conditions that might interfere with the erythropoietic response or had been administered RBC transfusions within 8 weeks of enrollment. Although no specific laboratory criteria for inflammatory or infectious disease were defined in the study protocol, clinicians were requested to exercise clinical judgment regarding patient exclusion based on underlying disease.
Canaud et al.	CERA	12 countries form Europe, Austrailia and Canada	Randomized Clinical Trial	313	NA	NA	29 to 36 weeks	Adult patients with chronic renal anaemia receiving adequate haemodialysis (Kt/V ≥1.2 or urea reduction ratio ≥ 65%) or peritoneal dialysis (weekly Kt/V ≥1.8) for ≥ 12 weeks and intravenous DA therapy at the same administration interval (either QW or Q2W) for ≥8 weeks were eligible for screening. The values for Hb entry criteria were within the ranges recommended by anaemia treatment guidelines at the time the study was designed. Adequate iron status (serum ferritin ≥100 ng/ml or transferrin saturation ≥20% or hypochromic red cells < 10%) was also required for study entry.	Patients were excluded if they had non-renal causes of anaemia (e.g. folic acid or vitamin B12 deficiency, haemolysis and haemoglobinopathies), C-reactive protein > 30 mg/l or life expectancy < 12 months.
Yamamoto et al. (2)	Molidustat	Japan	Randomized Clinical Trial	162	NA	NA	30 to 52 weeks	Patients included in the study were men or women aged 20 years or older who: had CKD with estimated glomerular filtration rate (eGFR) < 60 mL/min/1.73 m2 and were not undergoing dialysis or were not expected to start undergoing dialysis during the study period; were not receiving ESAs and/or HIF-PH inhibitors in the 8 weeks before randomization; and had mean central Hb levels, based on the last 2 screening measurements, of ≥8.0 g/dL and < 11.0 g/dL, with a difference between the 2 measurements (taken ≥2 days apart) of < 1.2 g/dL.	Key exclusion criteria included a history of cardiovascular or cerebrovascular events in the 6 months before randomization, sustained and poorly controlled arterial hypertension (systolic blood pressure [SBP] ≥180 mmHg or diastolic blood pressure ≥110 mmHg) or hypotension (SBP < 90 mmHg) at randomization stage, and presence of New York Heart Association Class III or IV congestive heart failure.
Carrera et al.	Methoxy polyethylene glycol-epoetin beta	Europe, Canada and Austrailia	Randomized Clinical Trial	335	Dec-06	Nov-07	50 to 53 weeks	Aged ≥18 years, had stable chronic renal anaemia (with a haemoglobin range of 11–13 g/dL) and were on regular haemodialysis (Fig. 1). To be included in the study, patients must have received haemodialysis three times weekly for ≥12 weeks before screening and during the 4-week screening/baseline period. Patients should also have had stable haemoglobin concentrations, defined as a maximum variation of 1 g/dL during the screening/baseline period. They must have undergone continuous once-weekly maintenance intravenous darbepoetin alfa therapy for ≥8 weeks before screening and during the screening/baseline period. Patients also had to have adequate iron status, defined as serum ferritin ≥100 μg/L, transferrin saturation ≥20% or < 10% hypochromic red blood cells.	Patients were excluded from the study if they had overt bleeding that necessitated red blood cell transfusion within 8 weeks of the start of screening or during the screening/baseline period; a non-renal cause of anaemia; C-reactive protein > 30 mg/L; the likelihood of early withdrawal; or life expectancy of < 12 months.
Tolman et al.	Epoetin	NA	Randomized Clinical Trial	169	NA	NA	9 months	All adult (18 yr or older), prevalent hemodialysis patients (receiving hemodialysis 90 d) were invited to participate, regardless of individual iron status, transfusion burden, Hb at randomization, or comorbidities.	Exclusion criteria were inability to give informed consent, receiving home hemodialysis, unsuitability for intravenous iron or erythropoietic agents, and uncontrolled hypertension at randomization (defined as a diastolic BP 100 mmHg).
Locatell et al.	rHuEP	Europe and Australia	Randomized Clinical Trial	166			24 weeks	Age ≥18 years, diagnosis of chronic renal insufficiency (CRI), rHuEPO-naive (no rHuEPO in past 12 weeks), hemoglobin ≤11.0 g/dL, adequate iron stores (serum ferritin ≥100 µg/L), serum vitamin B12 and folate levels above normal lower limit, creatinine clearance ≤30 mL/min.	Uncontrolled hypertension (diastolic BP > 100 mmHg), congestive heart failure (NYHA Class III or IV), hematologic disorders causing anemia, systemic infection, inflammatory diseases, other disorders interfering with NESP/rHuEPO response, recent blood transfusion or androgen therapy (within 8 weeks).
Hirakata et al.	Epoetin	Japan	Randomized Clinical Trial	171	Jul-04	Dec-05	16 and 32 weeks	The dose-response study enrolled adults with anemia (Hb\10 g/dl without administration of EPO in the last 4 weeks) and CKD [creatinine C2 mg/dl (177 lmol/l)] who were aged 20–80 years and weighed 40–80 kg, and who were not expected to initiate regular renal replacement therapy within 16 weeks.	Those with uncon-trolled hypertension, congestive heart failure [New York Heart Association (NYHA) class III–IV] and known history of symptomatic myocardial, pulmonary and cerebral infarction, unstable angina and obstructive arteriosclerosis (Fontaine’s class II–IV) were excluded together with those with malignancy, major bleeding, recent surgery, transfusion or investigational products within 16 weeks.
Vanrenterghem et al., 2002.	Darbepoetin alfa	European and Australian dialysis units.	Multicenter, Randomized, Open-Label Trial	522	November 1997.	Jul-98	Total: 52 weeks.Evaluated at weeks 25–32.	The study included adults aged 18 years and older with chronic renal failure (CRF) who had been on stable hemodialysis (HD) or peritoneal dialysis (PD) for at least six months. Participants were required to have been on stable recombinant human erythropoietin (rHuEPO) therapy for a minimum of three months. Baseline hemoglobin (Hb) levels had to be within the range of 9.5–12.5 g/dL, and serum ferritin levels needed to be ≥ 100 µg/L to ensure adequate iron stores.	Patients were excluded if they had any conditions that could interfere with erythropoiesis, had received a red blood cell (RBC) transfusion within the past month, or had hematological, inflammatory, or infectious disorders.
Wen-Yi Li et al., 2008.	Darbepoetin Alfa	Taiwan	Open-label, single-center, randomized comparative study.	45	NA	NA	Total duration: 5.5 months. Dose Titration Period: First 4 months. Evaluation Period: Final 1.5 months.	The study included patients aged 18 years or older with end-stage renal disease (ESRD) who had been on peritoneal dialysis (PD) for at least 3 months. Patients were required to have a stable subcutaneous r-HuEPO therapy for 3 months prior to enrollment and hemoglobin levels of 8.0–12.0 g/dL. Iron stores were confirmed as sufficient (serum ferritin > 100 ng/mL, transferrin saturation ≥20%).	Patients were excluded if they had uncontrolled hypertension (diastolic blood pressure > 100 mmHg), New York Heart Association (NYHA) class III or IV heart failure, active liver disease, severe hyperparathyroidism (intact PTH ≥800 pg/mL), or hematologic, infectious, or inflammatory conditions. Pregnant or breastfeeding women and those who had received blood transfusions, androgen therapy, or major surgery within 1–3 months were also excluded.
Furukawa et al., 2015	Darbepoetin Alfa	Japan	Open-label, randomized, single-center comparative study.	20	NA	NA	48 weeks	Patients with chronic kidney disease (CKD) not on dialysis who had been receiving darbepoetin alfa for ≥24 weeks before the study. Baseline hemoglobin levels were required to be stable (10–12 g/dL).	Patients with uncontrolled hypertension, significant inflammation or infection, malignancy, or conditions interfering with erythropoiesis were excluded
Simon D. Roger et al., 2014	Aranesp® (Darbepoetin Alfa)	Australia and Canada.	Multicenter, randomized, single-blind, placebo-controlled, parallel-group study.	51	NA	NA	36 weeks.	he study included patients aged ≥70 years with predialysis CKD (Stages 3–5), an estimated glomerular filtration rate (eGFR) between 10 and 60 mL/min/1.73 m^2^, and hemoglobin (Hb) levels < 11.0 g/dL. Patients also needed transferrin saturation ≥15% and normal vitamin B12 and folate levels.	Patients were excluded if they had a clinical history of type 2 diabetes mellitus, were scheduled for renal replacement therapy or kidney transplantation within a year, or had a history of major cardiovascular events or ESA therapy within 12 weeks of enrollment. Other exclusions included active bleeding, uncontrolled hypertension, or hematologic disorders.
Shubhadeep D. Sinha et al., 2019	Darbepoetin Alfa	India.	Randomized, Open-Label, Parallel-Group, Phase-III Clinical Trial	126	NA	NA	36 week	The study included adult patients with chronic kidney disease (CKD) on maintenance dialysis (hemodialysis or peritoneal dialysis) with hemoglobin levels between 7 and 10 g/dL. Patients were required to have adequate iron stores (serum ferritin ≥100 ng/mL and transferrin saturation ≥20%).	Patients with uncontrolled hypertension, recent blood transfusion, active malignancy, severe infection, or hematologic disorders were excluded. Pregnant or breastfeeding women were also excluded.
Nan Chen et al., 2021 Study	Darbepoetin Alfa	across multiple centers in China.	Multicenter, randomized, open-label, parallel-group study.	95			24 weeks	The study included adult patients with anemia secondary to chronic kidney disease (CKD) who were on maintenance dialysis (hemodialysis or peritoneal dialysis). Patients were required to have baseline hemoglobin levels between 7.0 and 10.0 g/dL and adequate iron stores (serum ferritin ≥100 ng/mL and transferrin saturation ≥20%).	Patients were excluded if they had uncontrolled hypertension, recent blood transfusions, active malignancy, hematologic disorders, or infections. Pregnant or breastfeeding women were also excluded.
Michèle Kessler et al., 2010	C.E.R.A.	Europe, North America, and other regions.	Open-label, randomized, multicenter, parallel-group, Phase 3 trial.	324			52 WEEK	Patients eligible for the study were adults aged 18 years or older with chronic kidney disease (CKD) stages 3 or 4 who were not on dialysis. They had baseline hemoglobin (Hb) levels between 8 and 11 g/dL and demonstrated adequate iron status, defined as a serum ferritin level of at least 100 ng/mL or transferrin saturation of at least 20%.	Patients were excluded if they were experiencing rapid CKD progression, defined as a decrease in creatinine clearance of more than 20% within 12 weeks, or if dialysis was anticipated within six months of study initiation. Additional exclusion criteria included recent erythropoiesis-stimulating agent (ESA) use or blood transfusion within the preceding 12 weeks, overt bleeding that necessitated transfusion, and a life expectancy of less than 12 months.
Michèle Kessler et al., 2010	C.E.R.A.	Europe, North America, and other regions.	Open-label, randomized, multicenter, parallel-group, Phase 3 trial.	324			52 WEEK Core period: 28 weeks. Extension period: 24 weeks.	Patients eligible for the study were adults aged 18 years or older with chronic kidney disease (CKD) stages 3 or 4 who were not on dialysis. They had baseline hemoglobin (Hb) levels between 8 and 11 g/dL and demonstrated adequate iron status, defined as a serum ferritin level of at least 100 ng/mL or transferrin saturation of at least 20%.	Patients were excluded if they were experiencing rapid CKD progression, defined as a decrease in creatinine clearance of more than 20% within 12 weeks, or if dialysis was anticipated within six months of study initiation. Additional exclusion criteria included recent erythropoiesis-stimulating agent (ESA) use or blood transfusion within the preceding 12 weeks, overt bleeding that necessitated transfusion, and a life expectancy of less than 12 months.
Marc A. Pfeffer et al., 2009	Darbepoetin Alfa	North America, Europe, Asia, and Australia.	Randomized, double-blind, placebo-controlled trial.	4038			Median follow-up: 29.1 months.	Participants included in the study were adults with Type 2 diabetes and chronic kidney disease (CKD) with an estimated glomerular filtration rate (eGFR) between 20 and 60 mL/min/1.73 m^2^. Eligible participants had hemoglobin levels between 9.0–12.0 g/dL, transferrin saturation of at least 15%, and serum ferritin levels of at least 100 ng/mL.	Participants were excluded if they were dependent on dialysis, had uncontrolled hypertension, or had recent cardiovascular events or a cancer diagnosis. Additionally, those who had used erythropoiesis-stimulating agents within the previous 12 weeks were not eligible for the study.
Laura Kooienga et al., 2024	Vadadustat	North America.	Randomized, open-label, parallel-group study.	319			PEP20 TO 28 WEEK.SEO 46 TO 52	Participants included in the study were adult patients aged 18 years or older who were diagnosed with anemia related to chronic kidney disease (CKD) and undergoing hemodialysis. Eligible participants had baseline hemoglobin levels between 9.0–11.0 g/dL and stable iron status, defined as a transferrin saturation (TSAT) of at least 20% and serum ferritin of at least 100 ng/mL.	Patients were excluded if they had uncontrolled hypertension, recent or active bleeding, a known malignancy, or severe cardiovascular events within the previous 12 weeks. Additionally, individuals with prior use of hypoxia-inducible factor prolyl hydroxylase inhibitors (HIF-PHIs) were not eligible for the study.
Laura Kooienga et al., 2024	Vadadustat	North America.	Randomized, open-label, parallel-group study.	319			PEP20 TO 28 WEEK.SEO 46 TO 52	Participants included in the study were adult patients aged 18 years or older who were diagnosed with anemia related to chronic kidney disease (CKD) and undergoing hemodialysis. Eligible participants had baseline hemoglobin levels between 9.0–11.0 g/dL and stable iron status, defined as a transferrin saturation (TSAT) of at least 20% and serum ferritin of at least 100 ng/mL.	Patients were excluded if they had uncontrolled hypertension, recent or active bleeding, a known malignancy, or severe cardiovascular events within the previous 12 weeks. Additionally, individuals with prior use of hypoxia-inducible factor prolyl hydroxylase inhibitors (HIF-PHIs) were not eligible for the study.
Macdougall et al., 2008 Study	C.E.R.A.	Europe,US,canada astralia	Randomized, open-label, parallel-group, Phase 3 study.	324	Jun-04	Jan-06	Duration of Follow-up: Correction period: 18 weeks. **Evaluation period: 10 weeks.** Extension period: 24 weeks (total follow-up: 52 weeks)	The study included adults aged 18 years or older with chronic kidney disease (CKD) who were undergoing hemodialysis. Eligible participants had been previously treated with stable doses of epoetin for at least 12 weeks and maintained baseline hemoglobin levels between 10.0–13.0 g/dL.	Participants were excluded if they had rapidly progressing kidney disease, severe cardiovascular instability, active bleeding, or had recently received a blood transfusion. Additional exclusions included a known malignancy or any other condition that could interfere with the study outcomes.
Yamamoto et al., 2021	Molidustat	Japan	Randomized, open-label, parallel-group, multicenter, Phase 3 |	164	NA	NA	52 weeks (primary endpoint evaluated at weeks 30–36)	Non-dialysis CKD patients (stages 3–5) treated with ESA; Hb levels ≥10.0 g/dL and < 13.0 g/dL; eGFR < 60 mL/min/1.73 m^2^. |	Conditions causing significant blood loss, active hemolysis, and recent cardiovascular/cerebrovascular events.

**Table:** 

		Age(Years)				Male				Hb				Dialysis				Smoking			
		DA		Comparator		DA		Comparator		DA		comparator		DA		C		DA		C	
	Comparator agent name																				
		mean	SD	mean	SD	n	%	n	%	mean	SD	mean	SD	n	%	n	%	n	%	n	%
Singh et al.	Daprodustat	56	16.46	53.3	13.46	98	63	96	61	9.5	1	9.5	1	151	97	149	95	14	9	21	13
Liu et al.	Epoetin	47.79	12.33	49.03	12.49	149	55.26	75	64.41	11.07	0.79	11.16	0.7	152	99.2	59	99.8	NA	NA	NA	NA
Al-Ali et al.	Epoetin	58.7	14.1	57.3	15.6	29	55.7	36	56.3	11.5	0.35	11.5	0.34	NA	NA	NA	NA	NA	NA	NA	NA
Nissenson et al.	Epoetin	58	NA	57.8	NA	75	44	147	43	11.2	NA	11.2	NA	NA	NA	NA	NA	NA	NA	NA	NA
Canaud et al.	CERA	61.8	14.74	62.3	16.17	81	52	100	64	11.9	0.6	12	0.7	NA	NA	NA	NA	NA	NA	NA	NA
Yamamoto et al. (2)	Molidustat	71.2	10.1	72.1	9.3	50	62.5	50	61	10	0.61	9.84	0.64	NA	NA	NA	NA	7	8.8	10	12.2
Carrera et al.	Methoxy polyethylene glycol-epoetin beta	65.5	13.9	66.2	13.6	156	64	148	60	73.8	16.9	72.3	15.1	NA	NA	NA	NA	NA	NA	NA	NA
Tolman et al.	Epoetin	64	5.95	63	4.54	32	40	33	41	11.86	1.4	11.73	1.7	NA	NA	NA	NA	NA	NA	NA	NA
Locatell et al.	rHuEP	60.4	15	60.6	15.7	70	54	19	51	9.3	1	9.8	1.1	NA	NA	NA	NA	NA	NA	NA	NA
Hirakata et al.	Epoetin	55.55	12.75	55.15	13.17	19	44.2	12	27.9	8.56	0.8	8.6	0.755	NA	NA	NA	NA	NA	NA	NA	NA
Vanrenterghem et al., 2002.	Recombinant human erythropoietin (rHuEPO).	60.1	12.18	60.9	12.28	188	54	100	57	11	522	11	0,56	HD;318 PD;29	HD;92 PD 8	HD;163 PD;12	HD; 93 PD 7	NA	NA	NA	NA
Wen-Yi Li et al., 2008	Recombinant human erythropoietin (rHuEPO).	49.5	9.75	48	11.15	12	54.5	8	34.8	9.98	0.76	9.66	0.78	Continuous Ambulatory Peritoneal Dialysis (CAPD);17Automated Peritoneal Dialysis (APD);5	ontinuous Ambulatory Peritoneal Dialysis (CAPD):;77.3 Automated Peritoneal Dialysis (APD);22.7	Continuous Ambulatory Peritoneal Dialysis (CAPD):;17Automated Peritoneal Dialysis (APD);6	Continuous Ambulatory Peritoneal Dialysis (CAPD):;73.9 Automated Peritoneal Dialysis (APD);26.1	NA	NA	NA	NA
Furukawa et al., 2015	CERA (Continuous Erythropoietin Receptor Activator)	73.4	8.2	69.4	4	NA	NA	NA	NA	11.2	0.2	11.1	0.1	NA	NA	NA	NA	NA	NA	NA	NA
Roger et al., 2014	Placebo	79.2	4.9	81	5.1	15	54	14	61	10.28	1.01	9.97	1.01	NA	NA	NA	NA	NA	NA	NA	NA
Sinha et al., 2019	Erythropoietin Alfa (EPO)	44.8	11.81	48.8	12.6	39	61.9	46	73.02	8.39	0.9	8.8	0.89	63	100	63	100	NA	NA	NA	NA
Chen et al., 2021 Study	Epoetin Alfa	50.4	13	56	9.8	15	71.4	10	71.4	8.3	0.8	8.2	1	HD;21	HD; 100	HD;14	HD;100	NA	NA	NA	NA
Kessler et al., 2010	C.E.R.A. Q2W	66.9	12.8	63.9	13.8	74	49	30	41	12.1	1.11	12.2	1.14	NA	NA	NA	NA	NA	NA	NA	NA
Kessler et al., 2010	C.E.R.A. Q4W	66.9	12.8	63.5	14.9	74	49	30	42	12.1	1.11	12.2	1.14	NA	NA	NA	NA	NA	NA	NA	NA
Pfeffer et al., 2009)	Placebo	67.67	11.13	67.67	11.13	836	41.5	892	44	10.43	0.89	10.37	0.82	NA	NA	NA	NA	CS;185 FS;780	CS;9.2 FS;36.8	CS;192 FS;738	CS;9.5 FS;36.4
Kooienga et al., 2024	Vadadustat Once Daily	60.8	12.8	60.9	13.4	65	12.8	58	55.2	10.2	0.81	10.3	0.71	NA	NA	NA	NA	NA	NA	NA	NA
Kooienga et al., 2024	Vadadustat Three Times Weekly	60.8	12.8	61.2	12.5	65	12.8	60	56.6	10.2	0.81	10.2	0.74	NA	NA	NA	NA	NA	NA	NA	NA
Macdougall et al., 2008 Study	C.E.R.A.	66.9	12.8	63.9	14.1	80	49	70	43	10.2	0.7	10.2	0.6	NA	NA	NA	NA	NA	NA	NA	NA
Yamamoto et al., 2021	Molidustat	72.4	10.3	69	10.3	54	65.9	45	54.9	11.27	0.64	11.31	0.68	NA	NA	NA	NA	NA	NA	NA	NA

**Table 2:** Baseline characteristics of patients included

Study	Diabetes				Duration of diabetes				mean HbA1C				Albumin				Urine protein:creatinine ratio			
	DA		C		DA		C		DA		C		DA		C		DA		C	
	n	%	n	%	mean	SD	mean	SD	mean	SD	mean	SD	mean	SD	mean	SD	mean	SD	mean	SD
Singh et al.	70	45	70	45	NA	NA	NA	NA	NA	NA	NA	NA	3.7	0.37	3.7	0.52	NA	NA	NA	NA
Liu et al.	NA	NA	NA	NA	NA	NA	NA	NA	NA	NA	NA	NA	NA	NA	NA	NA	NA	NA	NA	NA
Al-Ali et al.	32	61.5	43	67.1	NA	NA	NA	NA	NA	NA	NA	NA	NA	NA	NA	NA	NA	NA	NA	NA
Nissenson et al.	62	37	116	34	NA	NA	NA	NA	NA	NA	NA	NA	NA	NA	NA	NA	NA	NA	NA	NA
Canaud et al.	22	14.1	25	15.9	NA	NA	NA	NA	NA	NA	NA	NA	NA	NA	NA	NA	NA	NA	NA	NA
Yamamoto et al. (2)	NA	NA	NA	NA	NA	NA	NA	NA	6	0.75	6.02	0.62	NA	NA	NA	NA	NA	NA	NA	NA
Carrera et al.	72	29	74	30	NA	NA	NA	NA	NA	NA	NA	NA	NA	NA	NA	NA	NA	NA	NA	NA
Tolman et al.	NA	NA	NA	NA	NA	NA	NA	NA	NA	NA	NA	NA	NA	NA	NA	NA	NA	NA	NA	NA
Locatell et al.	32	25	11	30	NA	NA	NA	NA	NA	NA	NA	NA	NA	NA	NA	NA	NA	NA	NA	NA
Hirakata et al.	NA	NA	NA	NA	NA	NA	NA	NA	NA	NA	NA	NA	NA	NA	NA	NA	NA	NA	NA	NA
Vanrenterghem et al., 2002.	NA	NA	NA	NA	NA	NA	NA	NA	NA	NA	NA	NA	NA	NA	NA	NA	NA	NA	NA	NA
Wen-Yi Li et al., 2008	NA	NA	NA	NA	NA	NA	NA	NA	NA	NA	NA	NA	NA	NA	NA	NA	NA	NA	NA	NA
Furukawa et al., 2015	NA	NA	NA	NA	NA	NA	NA	NA	NA	NA	NA	NA	3.9	0.1	3.9	0.1	1.3	0.4	1.5	0.5
Roger et al., 2014	NA	NA	NA	NA	NA	NA	NA	NA	NA	NA	NA	NA	NA	NA	NA	NA	NA	NA	NA	NA
Sinha et al., 2019	NA	NA	NA	NA	NA	NA	NA	NA	NA	NA	NA	NA	NA	NA	NA	NA	NA	NA	NA	NA
Chen et al., 2021 Study	NA	NA	NA	NA	NA	NA	NA	NA	NA	NA	NA	NA	NA	NA	NA	NA	NA	NA	NA	NA
Kessler et al., 2010	NA	NA	NA	NA	NA	NA	NA	NA	NA	NA	NA	NA	NA	NA	NA	NA	NA	NA	NA	NA
Kessler et al., 2010	NA	NA	NA	NA	NA	NA	NA	NA	NA	NA	NA	NA	NA	NA	NA	NA	NA	NA	NA	NA
Pfeffer et al., 2009)	2012	100	2026	100	15.1	10.09	15.13	9.87	7.1	1.41	7	1.26	NA	NA	NA	NA	0.83	1.41	0.77	1.26
Kooienga et al., 2024	74	68.5	66	62.9	NA	NA	NA	NA	NA	NA	NA	NA	NA	NA	NA	NA	NA	NA	NA	NA
Kooienga et al., 2024	74	68.5	69	65.1	NA	NA	NA	NA	NA	NA	NA	NA	NA	NA	NA	NA	NA	NA	NA	NA
Macdougall et al., 2008 Study	NA	NA	NA	NA	NA	NA	NA	NA	NA	NA	NA	NA	38.3	5.3	39.4	5.2	NA	NA	NA	NA
Yamamoto et al., 2021	NA	NA	NA	NA	NA	NA	NA	NA	NA	NA	NA	NA	NA	NA	NA	NA	NA	NA	NA	NA

#### Assessment of risk of bias

According to the ROB-2, the quality of the included studies was assessed as follows: the Eighteen studies were rated as having low quality, two as moderate quality and one as high quality [13]. The primary concerns identified were largely due to blinding, measurements of outcomes, and selection of the reported results in the studies as shown in the figure provided in Supplementary material Figures S1 and S2.

### Efficacy outcomes

#### Hemoglobin change

Mean change in hemoglobin levels from baseline to follow-up was the primary efficacy endpoint. Methoxy polyethylene glycol-epoetin beta demonstrated the greatest efficacy, with a mean hemoglobin increase of +1.04 g/dL and a SUCRA value of 0.91. Daprodustat showed a modest improvement (+0.067 g/dL; SUCRA 0.59), while CERA yielded a minimal change (−0.03 g/dL; SUCRA 0.45). Epoetin alfa and rHuEPO were associated with either minimal or negative changes, with SUCRA values between 0.23 and 0.50. Molidustat exhibited the least favorable response, with a mean decrease of −1.18 g/dL and a SUCRA value of 0.05. In additional analyses, Daprodustat significantly improved hemoglobin compared to darbepoetin (mean difference +0.15 g/dL, 95% CrI: 0.03 to 0.29), whereas Roxadustat showed a nonsignificant trend toward improvement (+0.12 g/dL, 95% CrI: −0.05 to 0.27) as shown in Fig. 2 (A-E)

**Fig. 2A:**
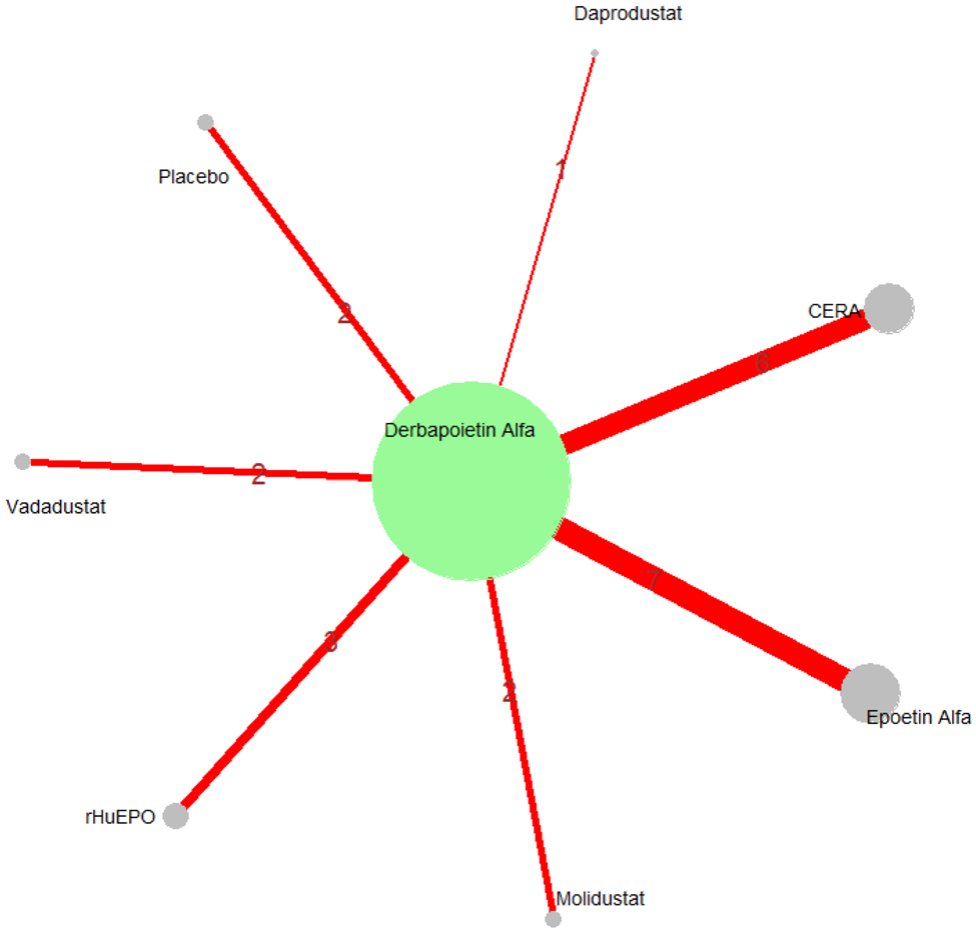
Network geometry plot

**Fig. 2B:**
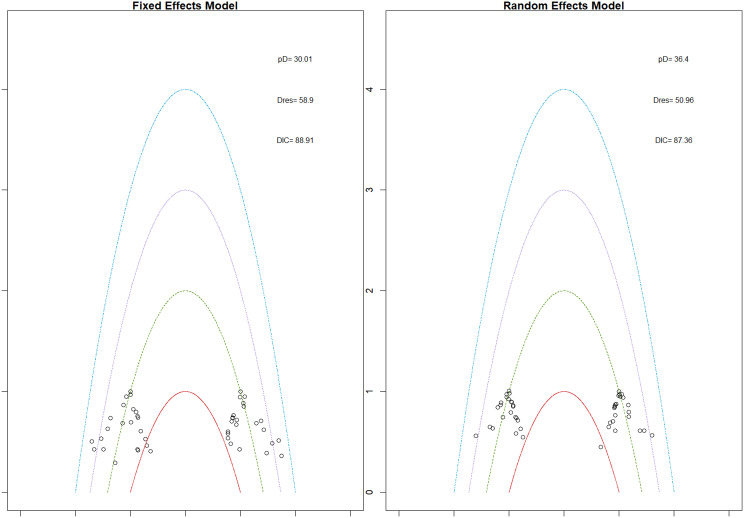
Rainbow plot

**Fig. 2C:**
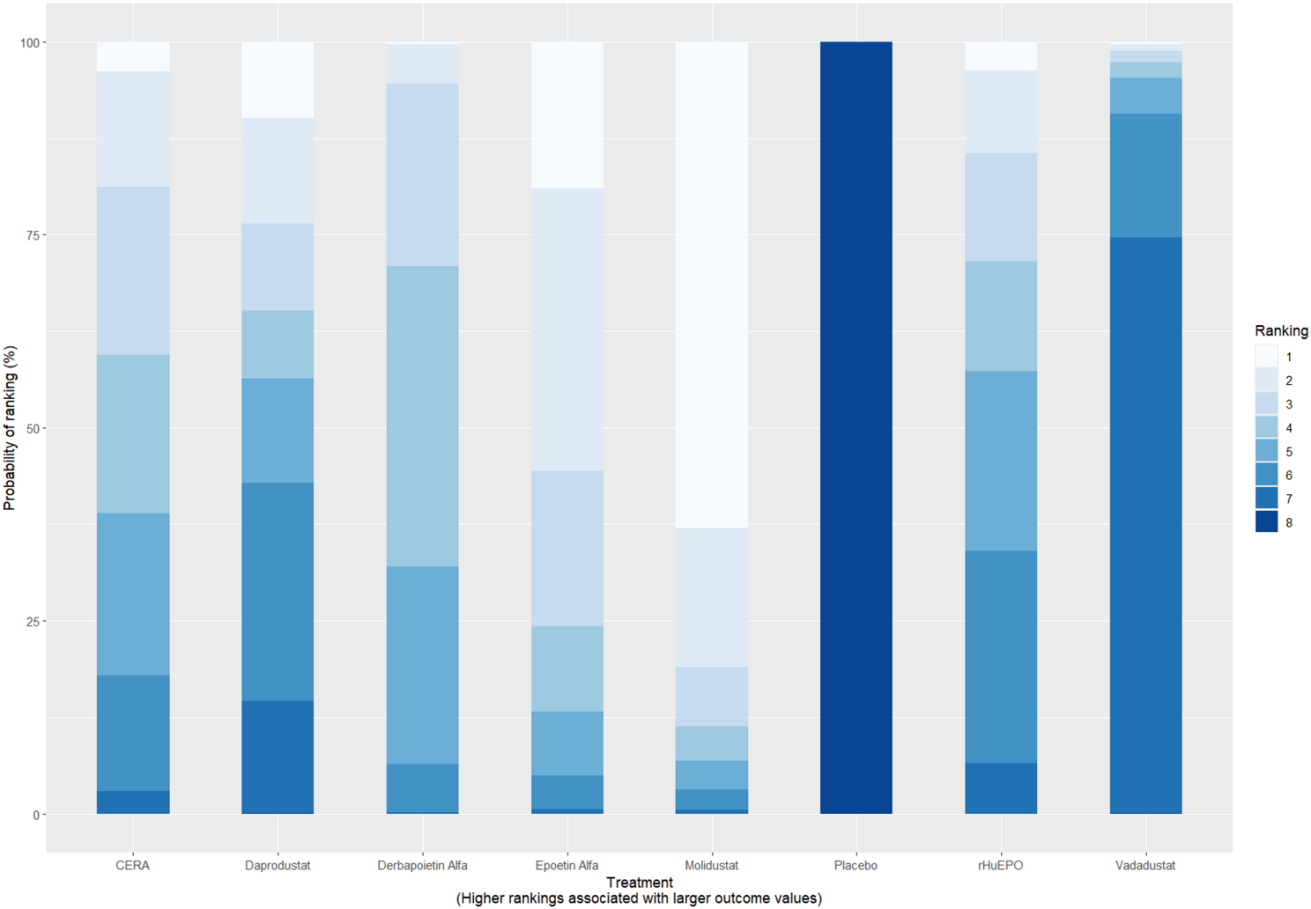
Rankogram

**Fig. 2D:**
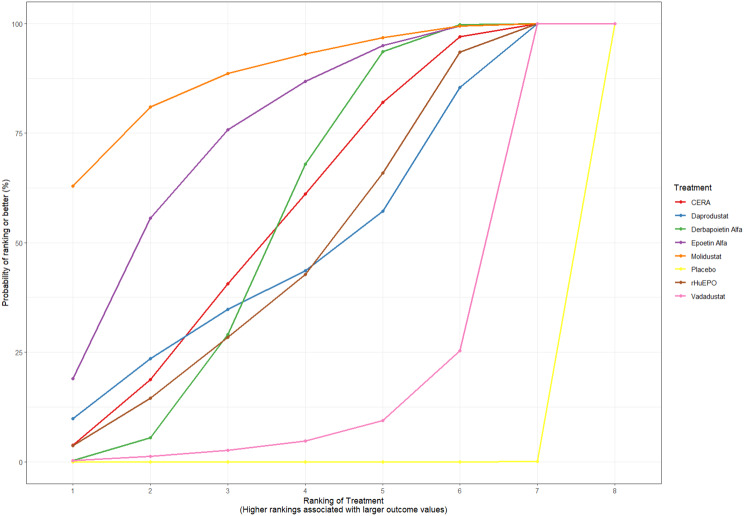
SUCRA plot

**Fig. 2E:**
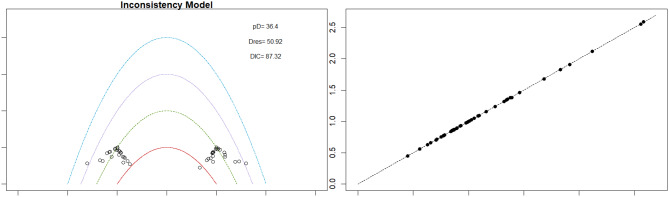
Model Consistency comparison plot

**Fig. 2F:**
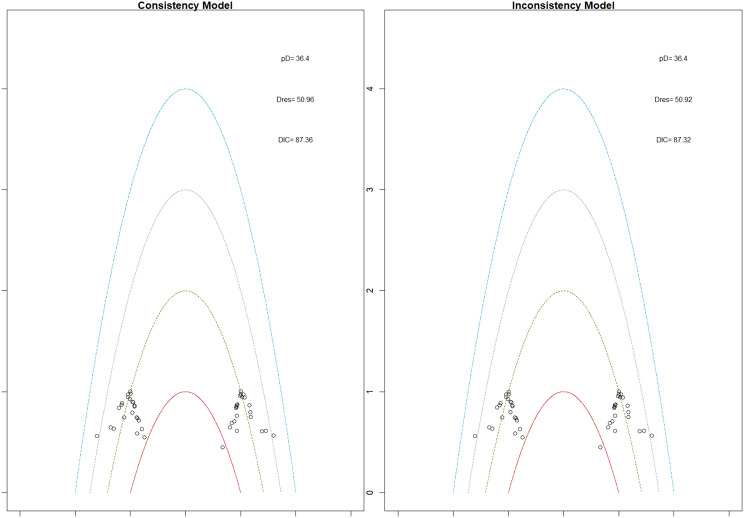
Model consistency and inconsistency rainbow plot

**Fig. 2G:**
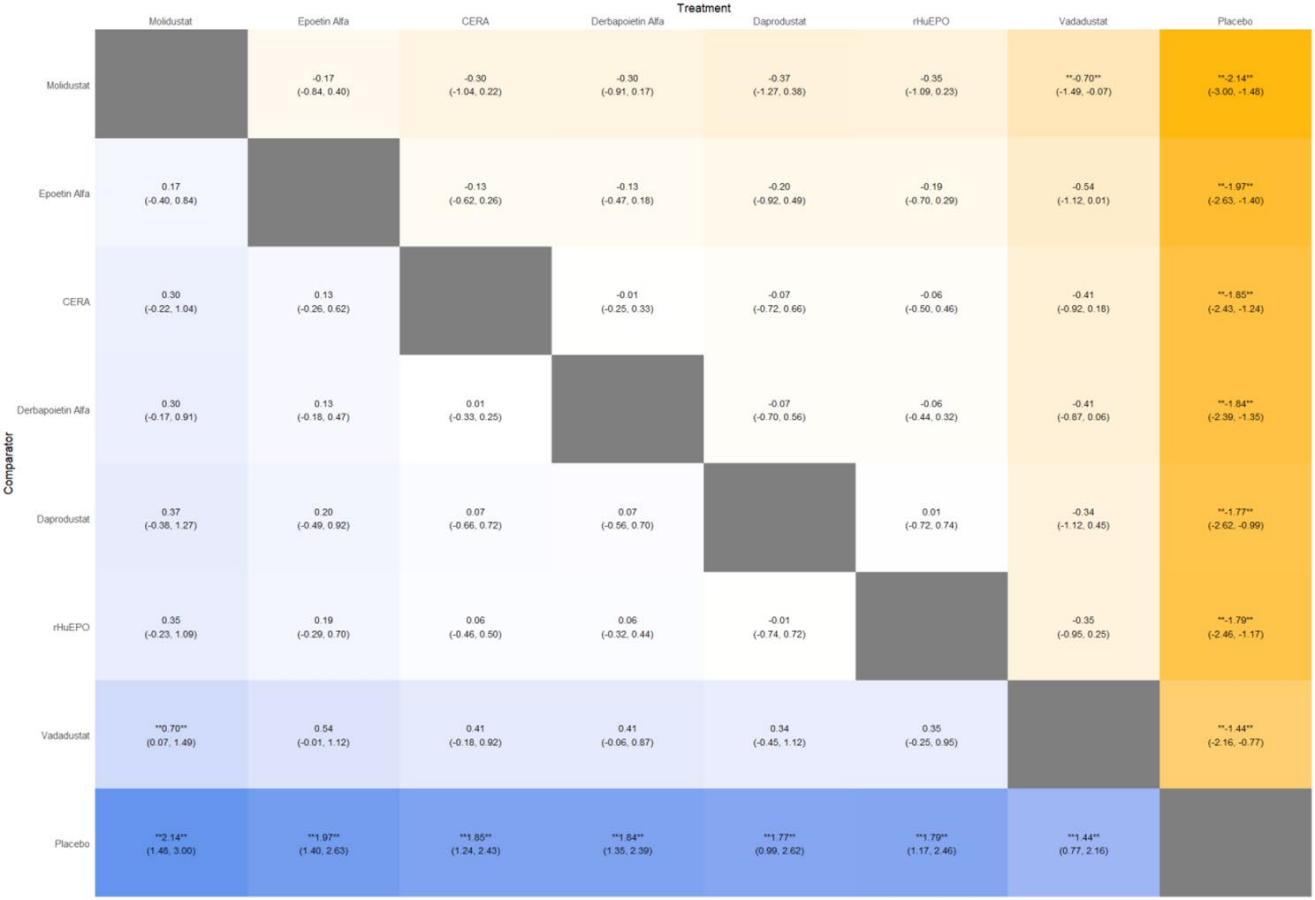
League table

**Fig. 2H:**
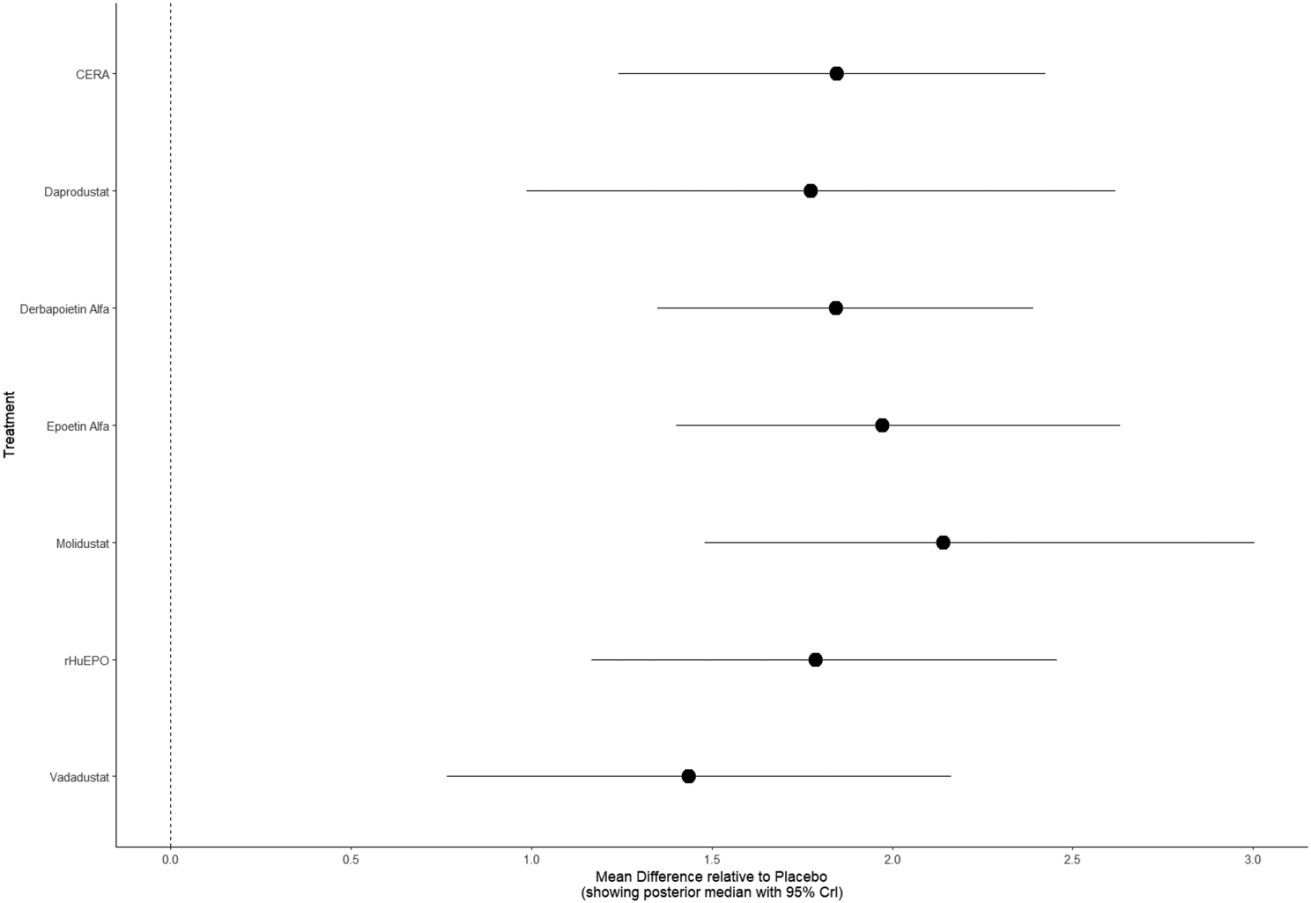
Forest plot

**Fig. 2I:**
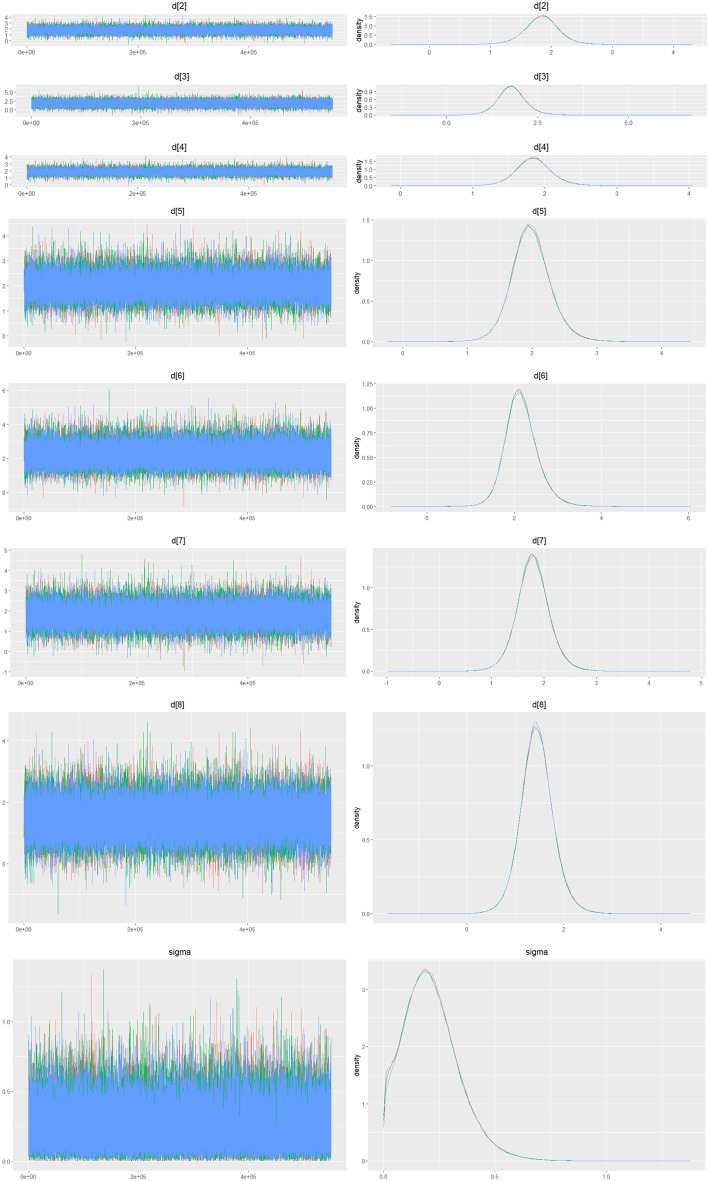
Density and trace plot

#### Transferrin saturation

Changes in transferrin saturation were modest across treatment arms. The pooled mean difference in transferrin saturation was +0.6% (95% CrI: −1.4 to +2.5), and no statistically significant differences were detected among interventions. Mean increases ranged from +1.8% to +2.4% across individual trials. SUCRA rankings for transferrin saturation showed little variation between agents, indicating that no treatment conferred a major advantage in terms of iron availability. The results are shown in Fig. 3 (A-I)

**Fig. 3A:**
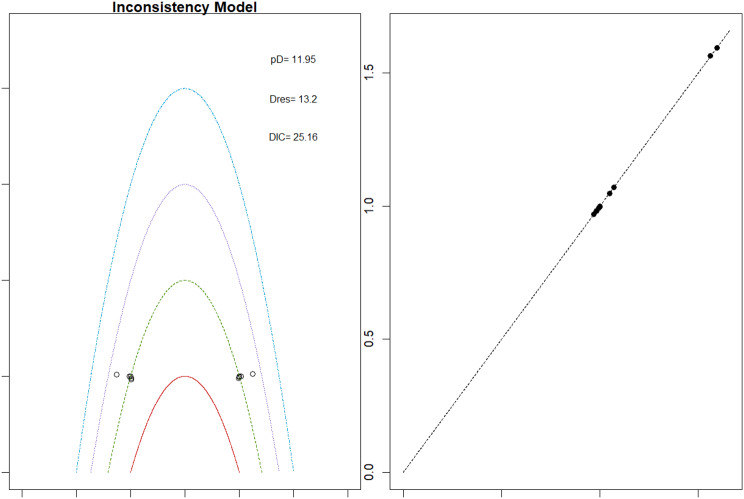
Consistency vs inconsistency comparison plot

**Fig. 3B:**
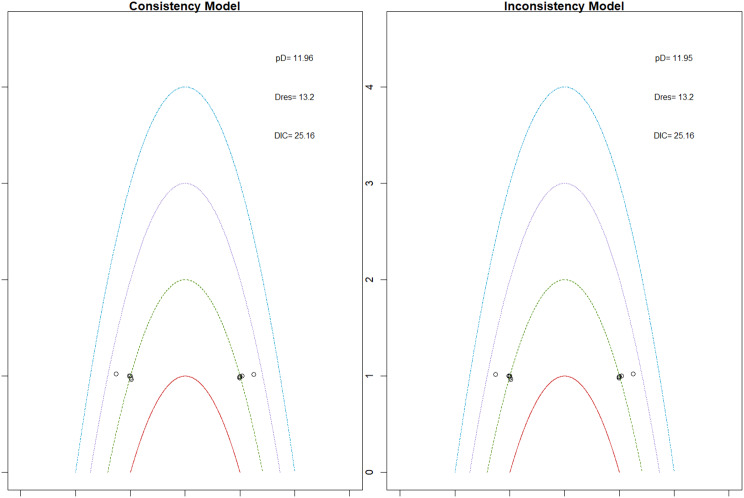
Consistency vs inconsistency rainbow plot

**Fig. 3C:**
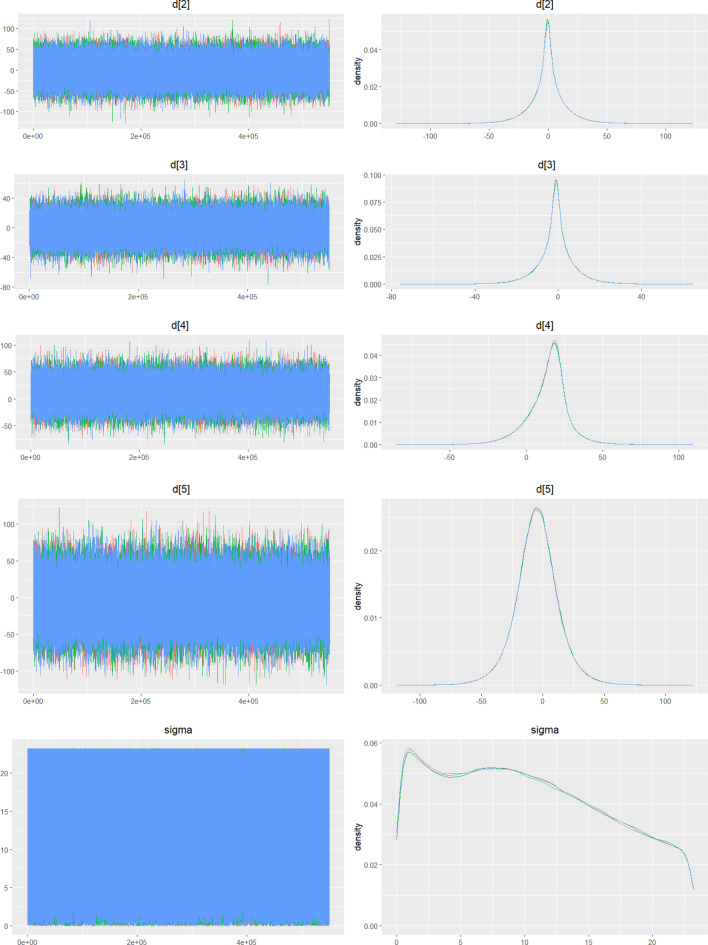
Density and trace plot

**Fig. 3D:**
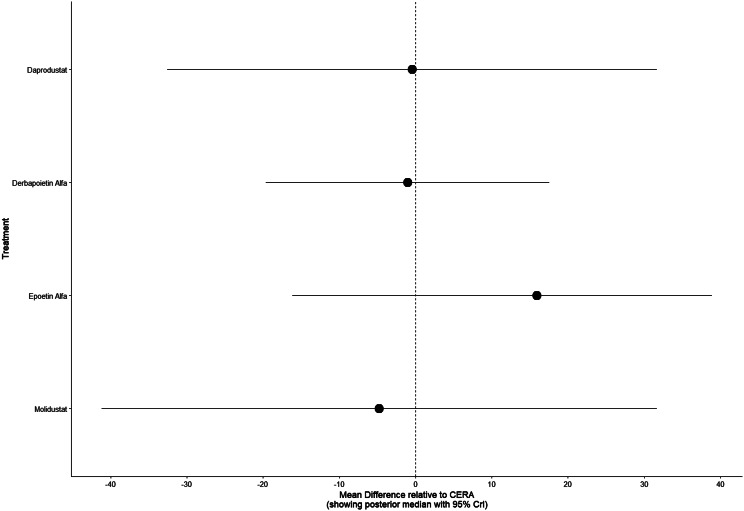
Forest plot

**Fig. 3E:**
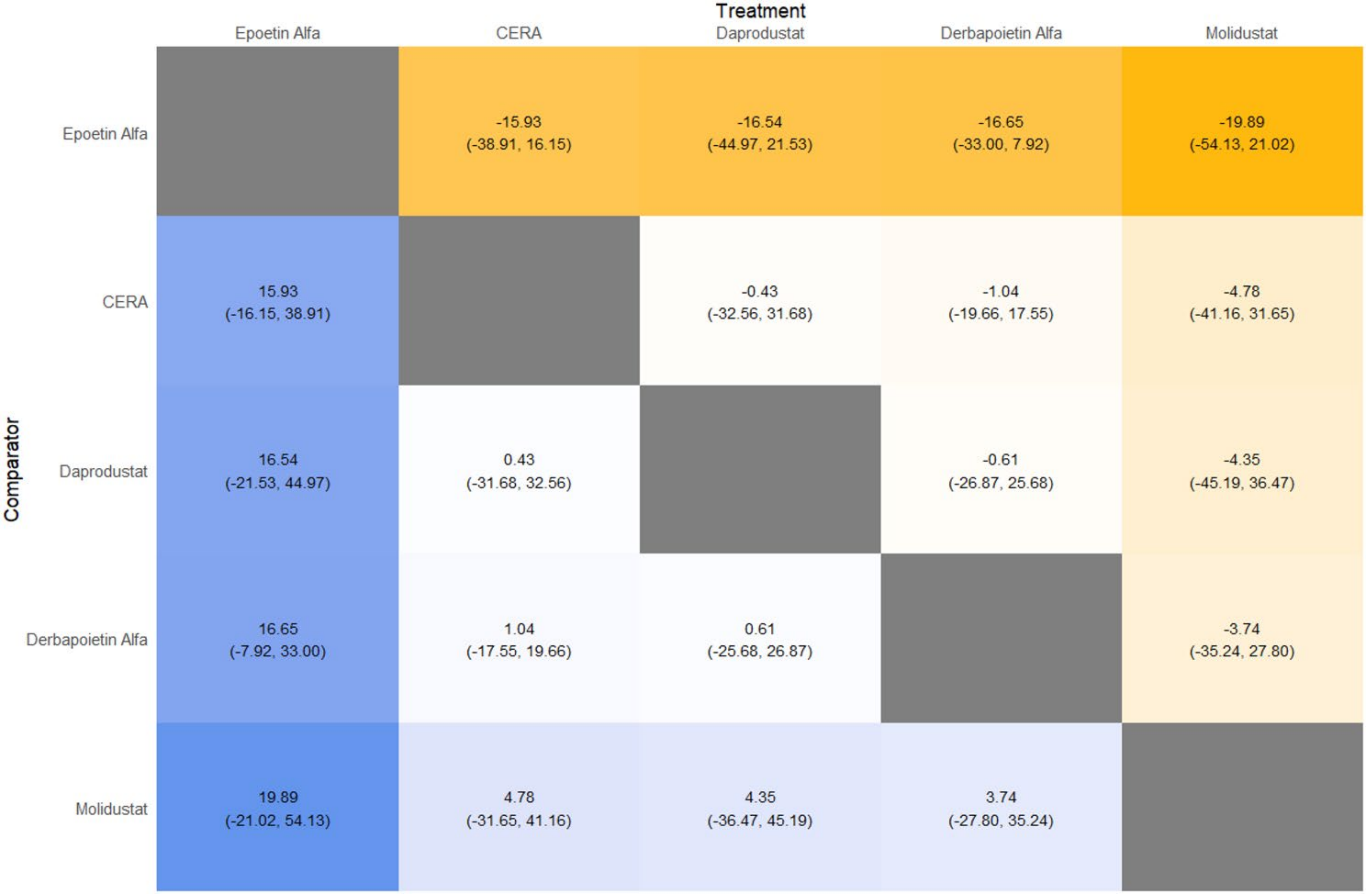
League table

**Fig. 3F:**
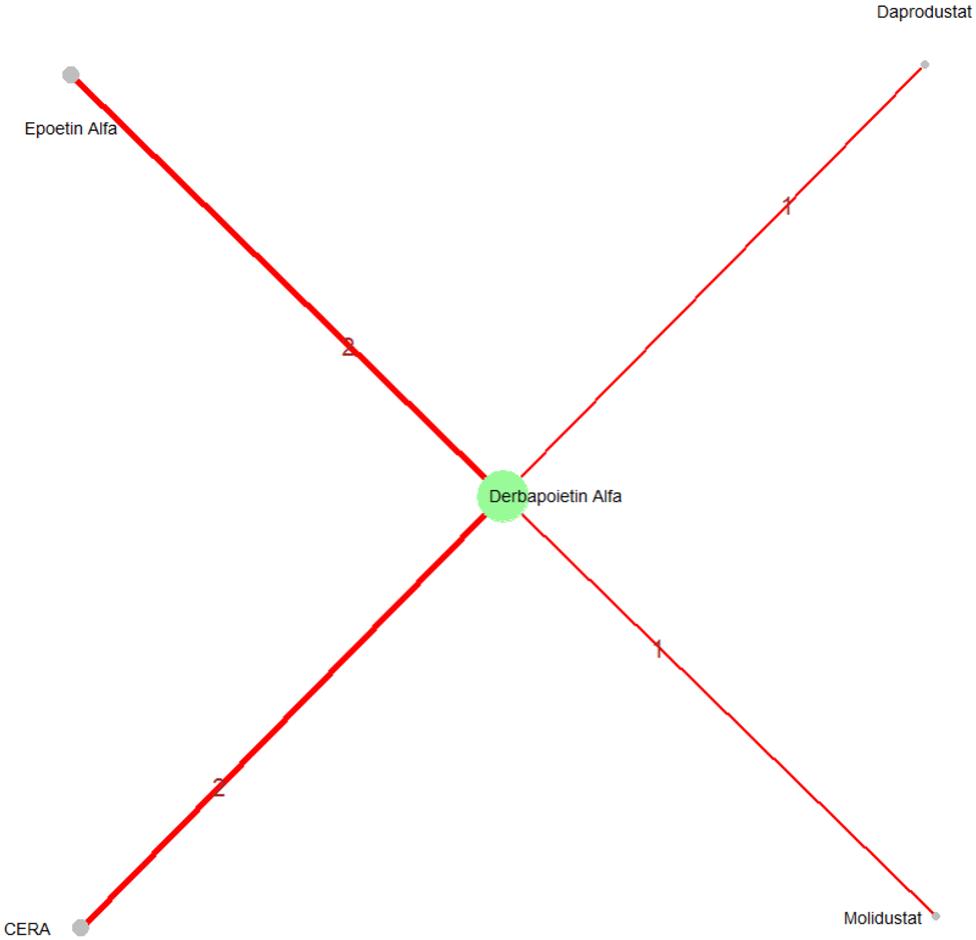
Network geometry

**Fig. 3G:**
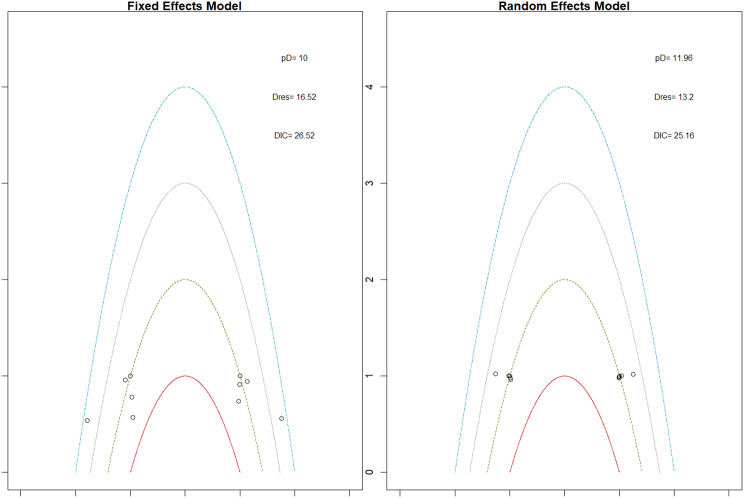
Rainbow plot main model

**Fig. 3H:**
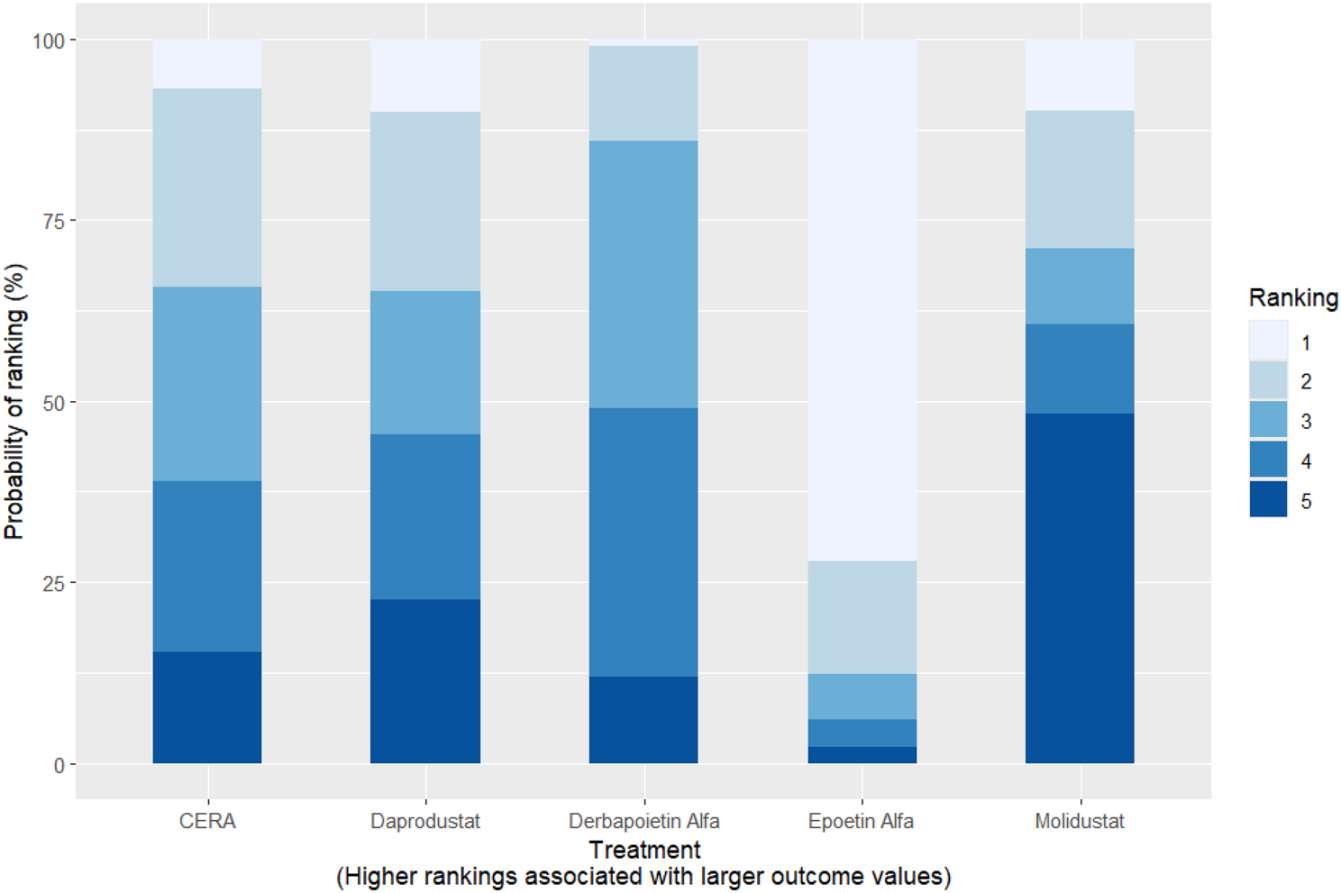
Rankogram

**Fig. 3I:**
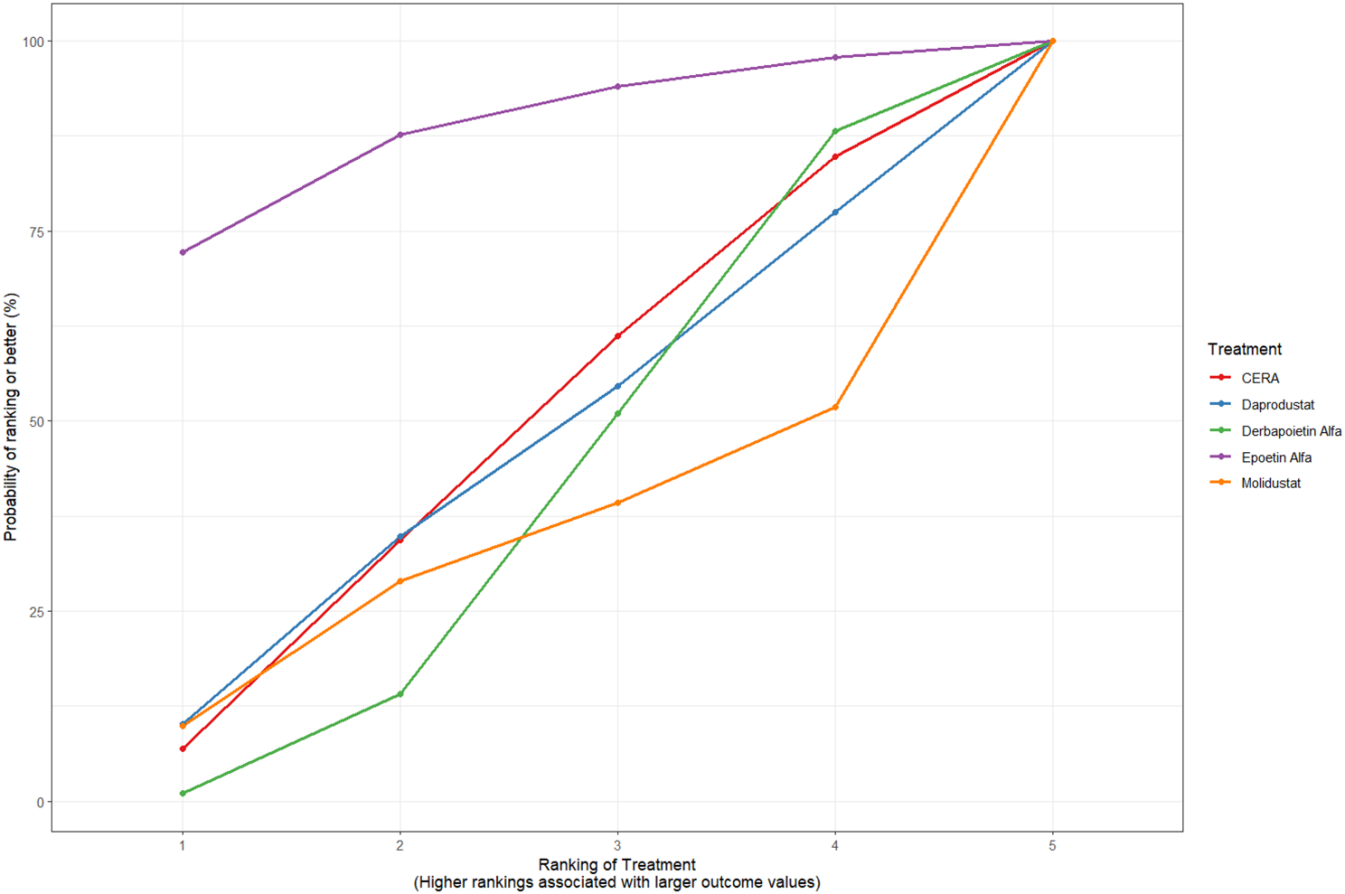
SUCRA plot

### Safety outcomes

#### Mortality

Mortality outcomes were reported across most included studies, but no statistically significant differences in mortality risk were observed between interventions. Daprodustat compared to darbepoetin yielded an odds ratio (OR) of 0.89 (95% CrI: 0.63 to 1.26), suggesting no excess mortality. Vadadustat exhibited a trend toward higher mortality compared to darbepoetin with an OR of 1.12 (95% CrI: 0.81 to 1.56), although this difference was not statistically significant. SUCRA values for mortality ranked Roxadustat highest, followed by Daprodustat and CERA, while darbepoetin, epoetin, and vadadustat ranked lower. The wide credible intervals indicated substantial uncertainty around mortality effects. The results are shown in Fig. 4 (A-F)

**Fig. 4A:**
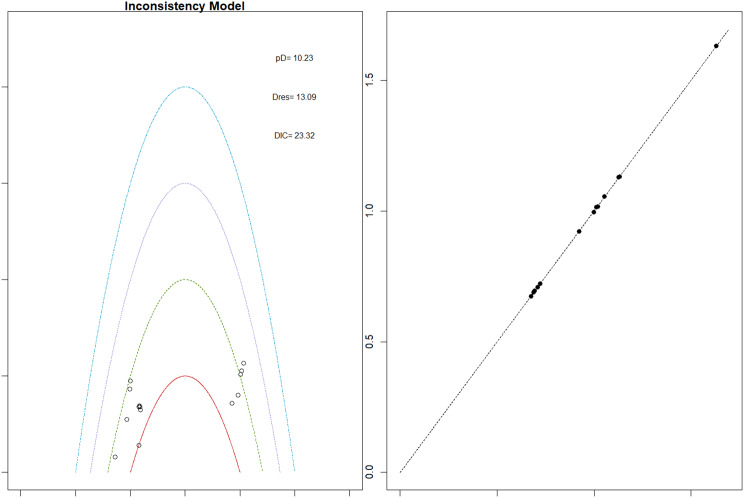
Consistency comparison plot

**Fig. 4B:**
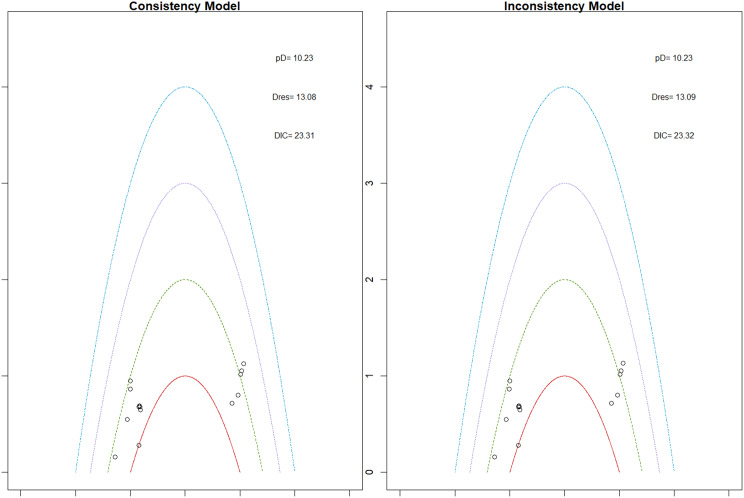
Consistency vs inconsistency model

**Fig. 4C:**
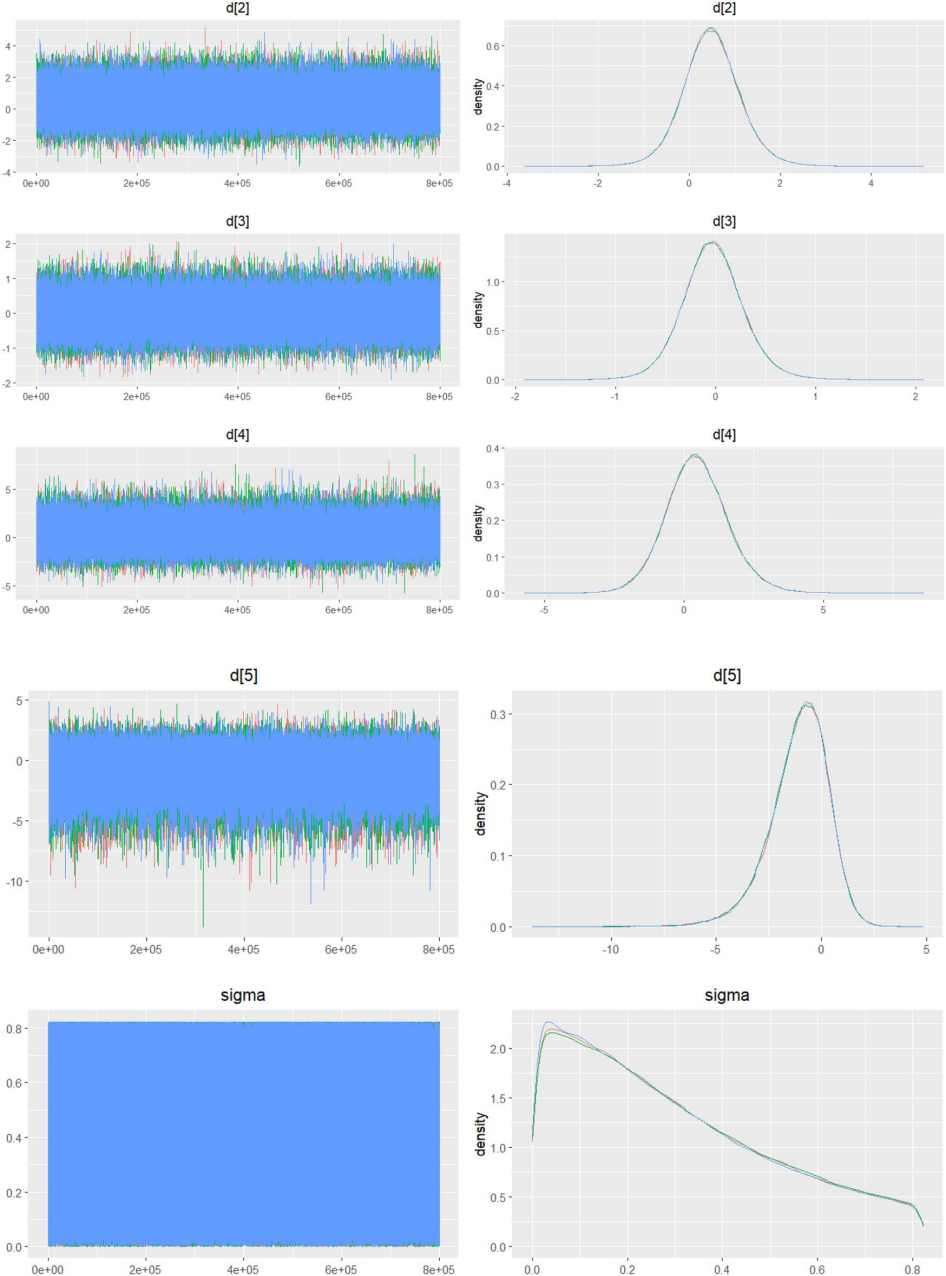
Density and Trace Plot

**Fig. 4D:**
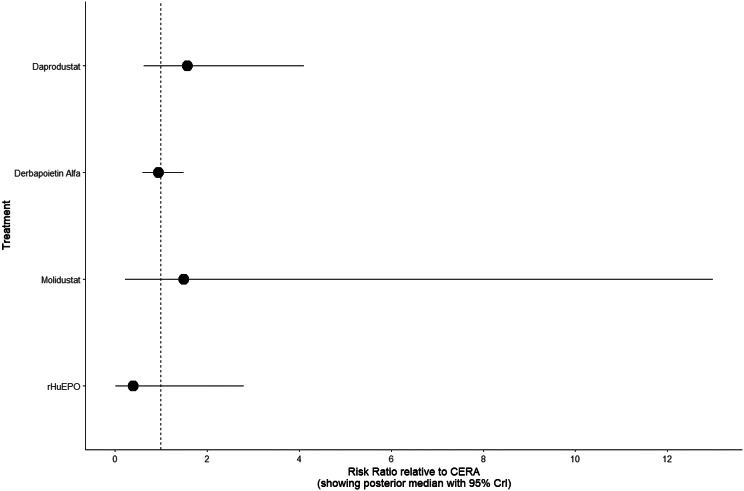
Forest plot

**Fig. 4E:**
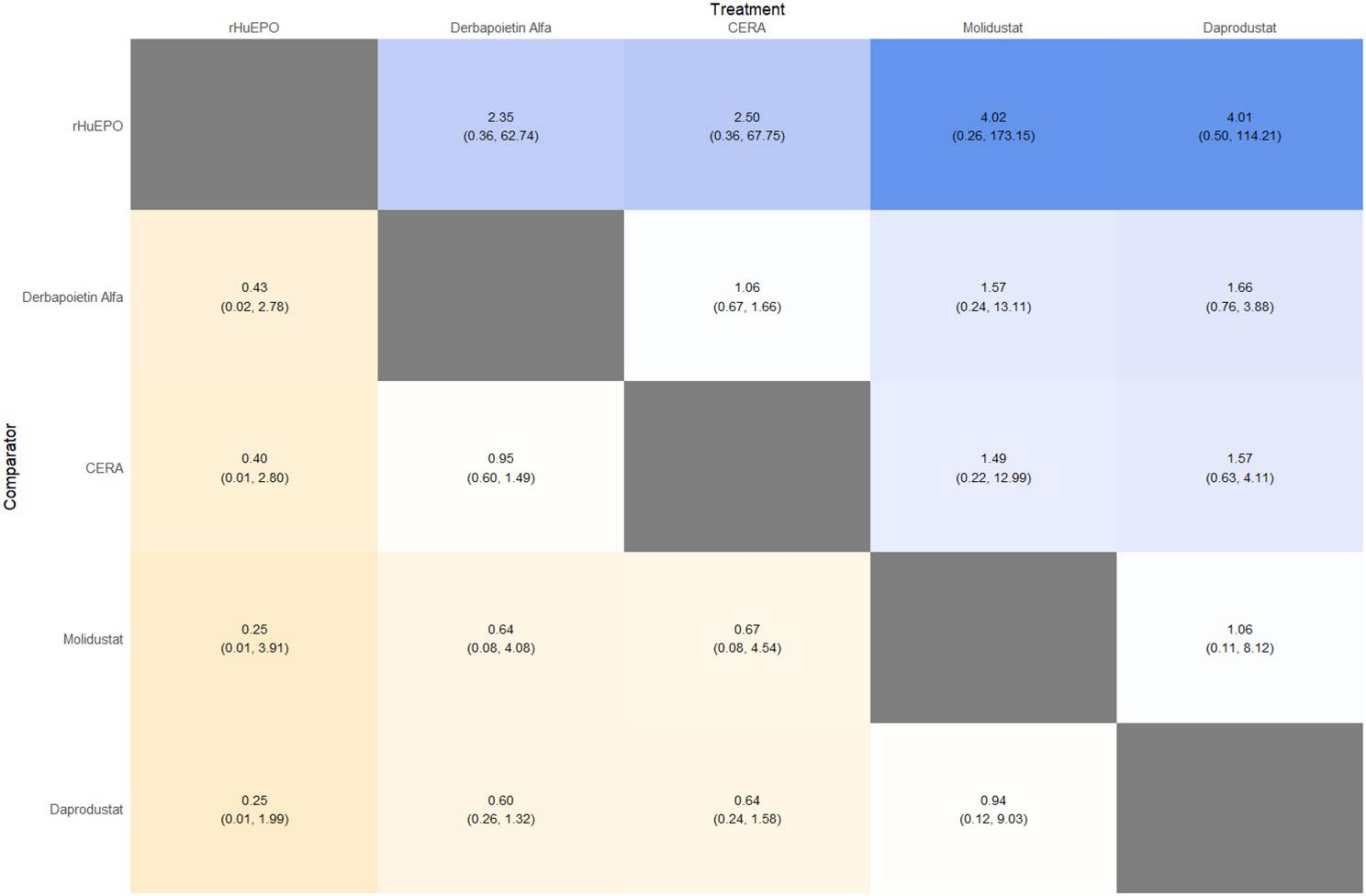
League table

**Fig. 4F:**
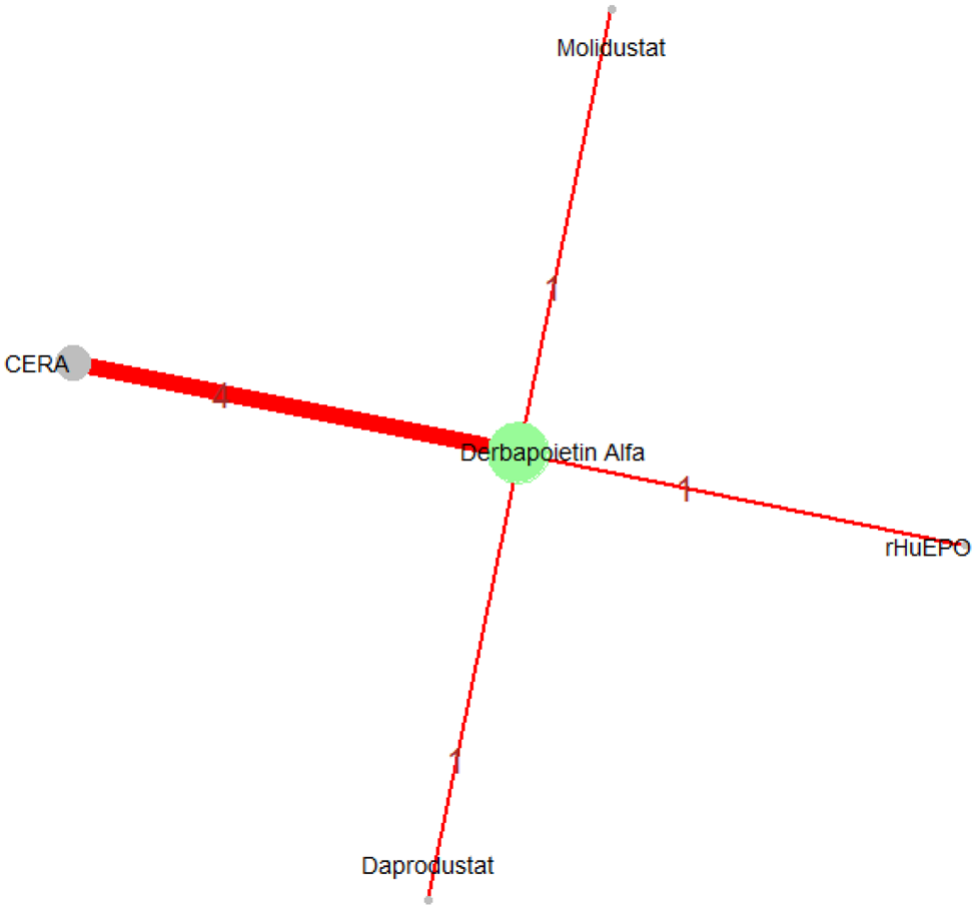
Network geometry

#### Cardiovascular events

Risk ratios for major cardiovascular events were evaluated across nearly all studies. Molidustat demonstrated the most favorable cardiovascular safety profile, with a risk ratio of 0.08 and a SUCRA value of 1.00, followed by CERA and Daprodustat. Daprodustat, in comparison to darbepoetin, displayed a neutral cardiovascular profile with an OR of 0.92 (95% CrI: 0.65 to 1.28). Vadadustat showed a non-significant trend toward higher cardiovascular risk, with an OR of 1.35 (95% CrI: 0.94 to 1.95). Roxadustat ranked highest for cardiovascular safety based on SUCRA analysis, while epoetin and vadadustat demonstrated less favorable rankings as shown in Fig. 5 (A-I)

**Fig. 5A:**
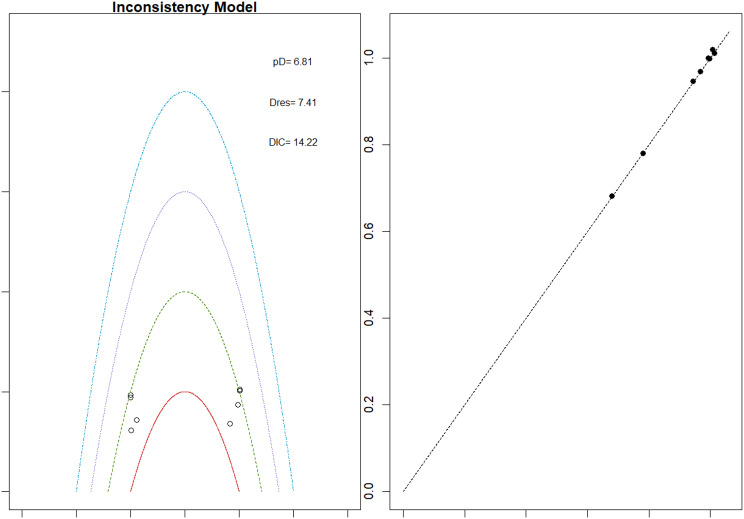
Consistency comparison plot

**Fig. 5B:**
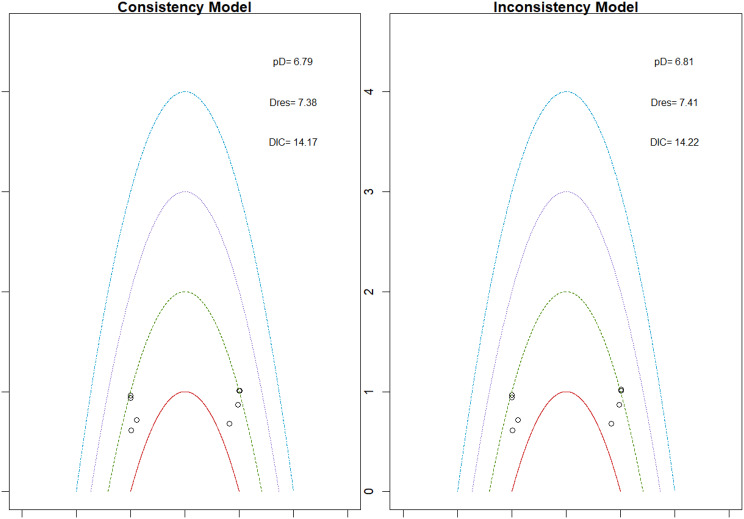
Consistency vs inconsistency plot

**Fig. 5C:**
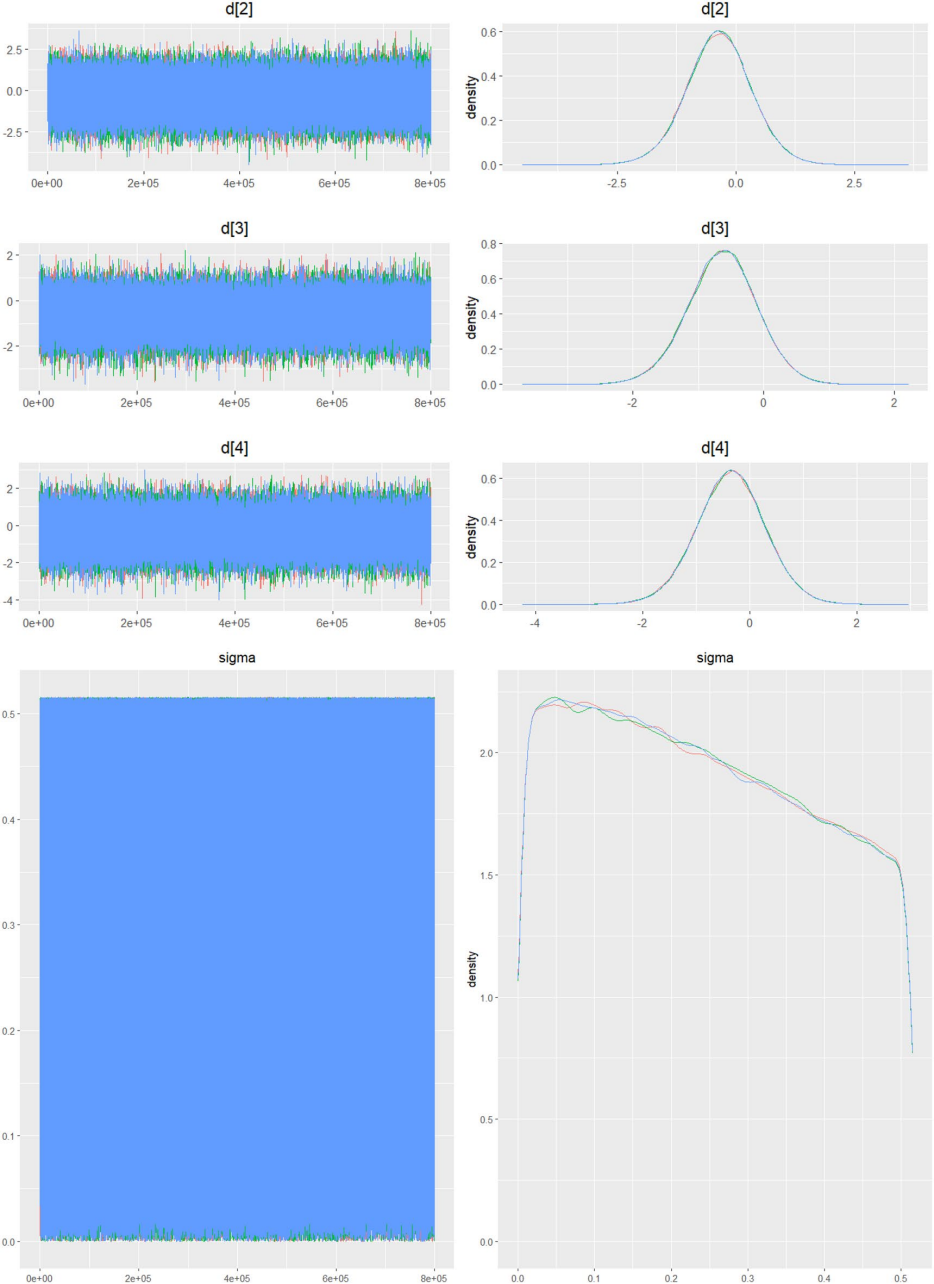
Density and trace plot

**Fig. 5D:**
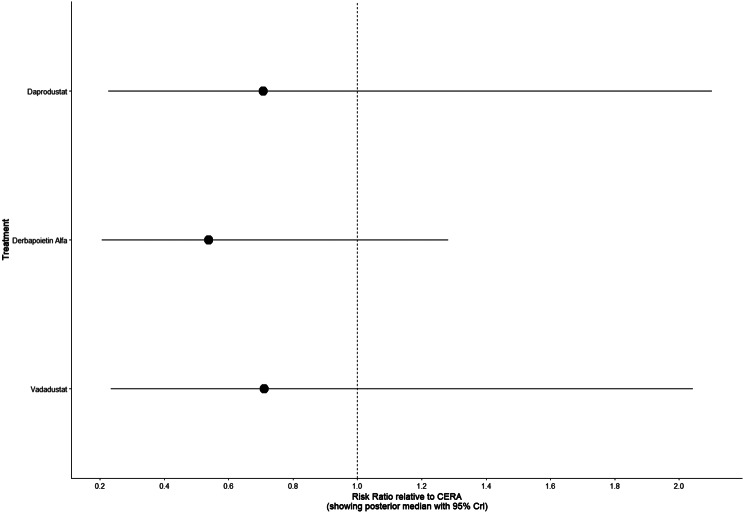
Forest plot

**Fig. 5E:**
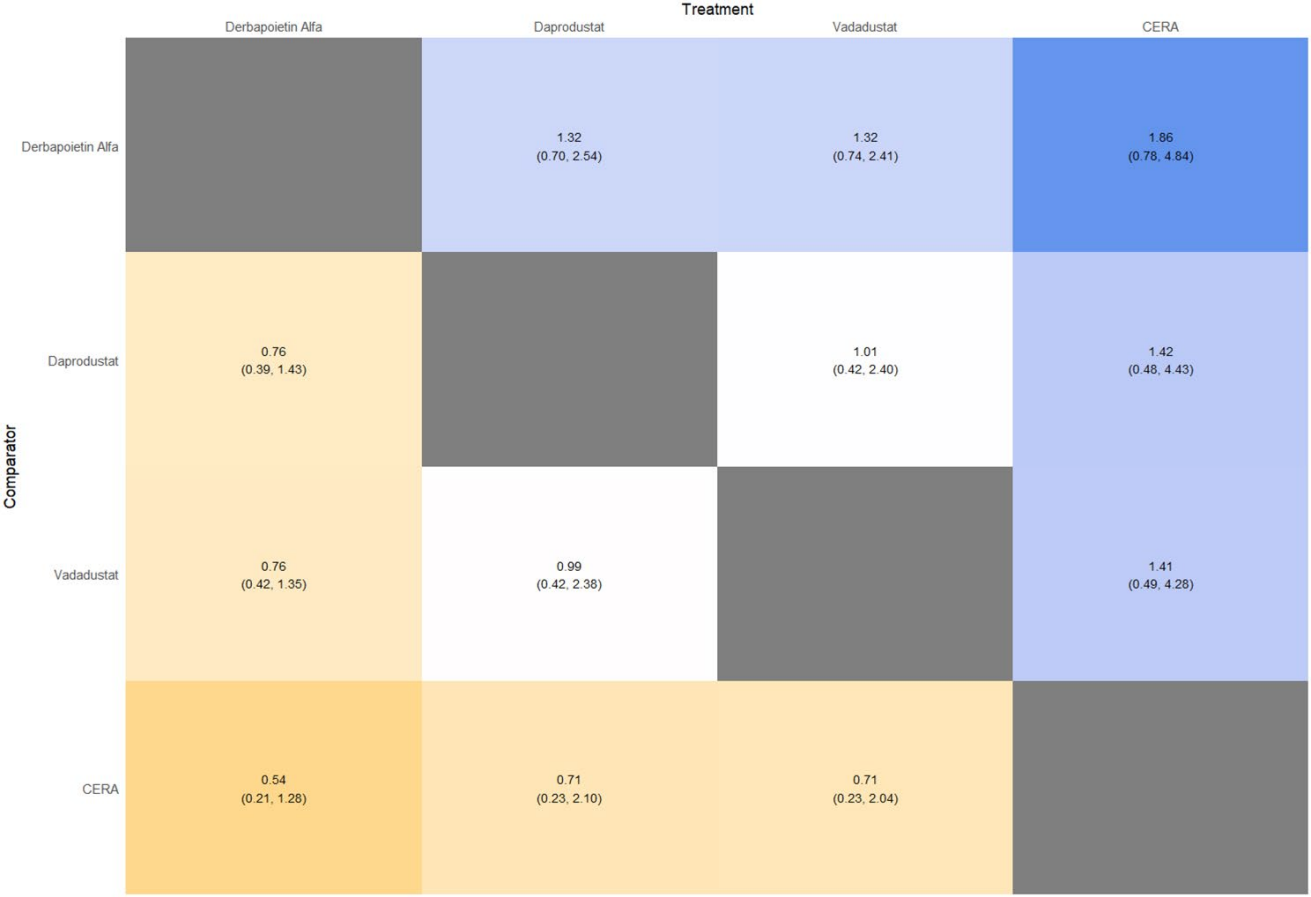
League table

**Fig. 5F:**
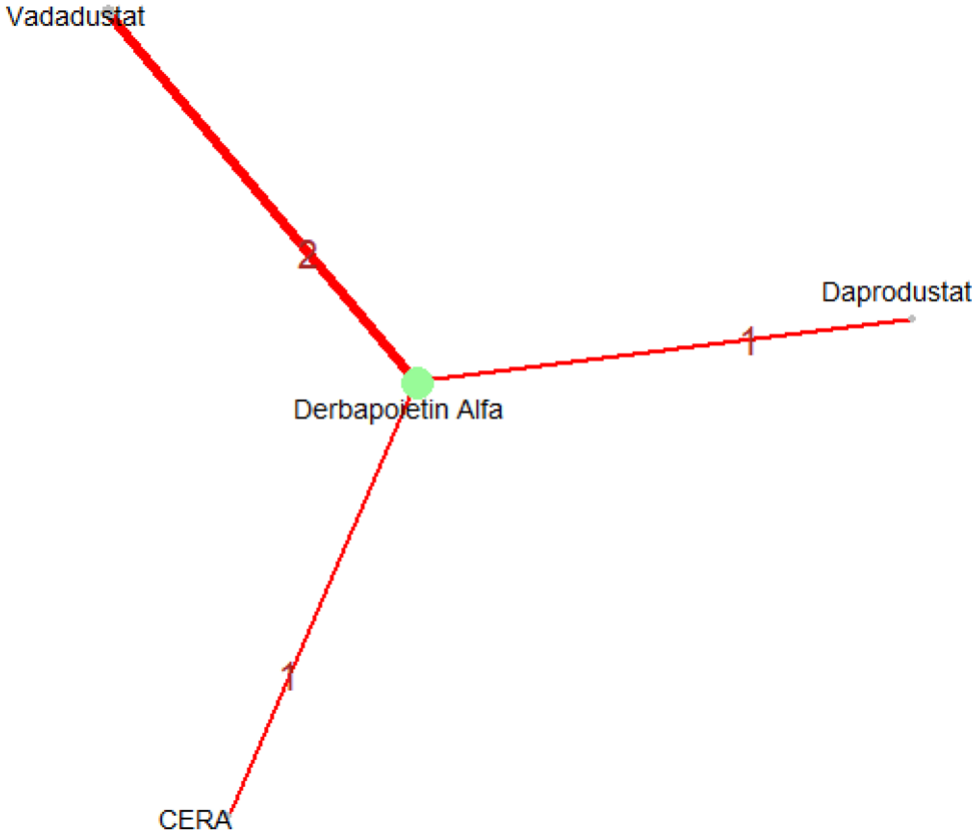
Network geometry plot

**Fig. 5G:**
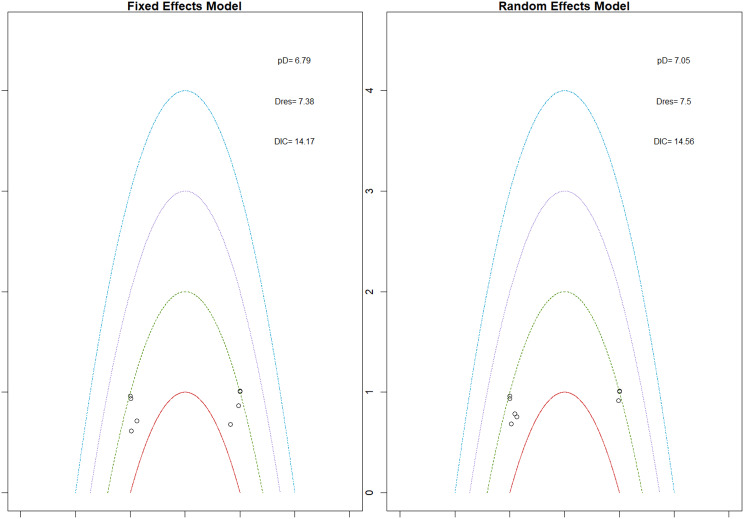
Rainbow plot main model

**Fig. 5H:**
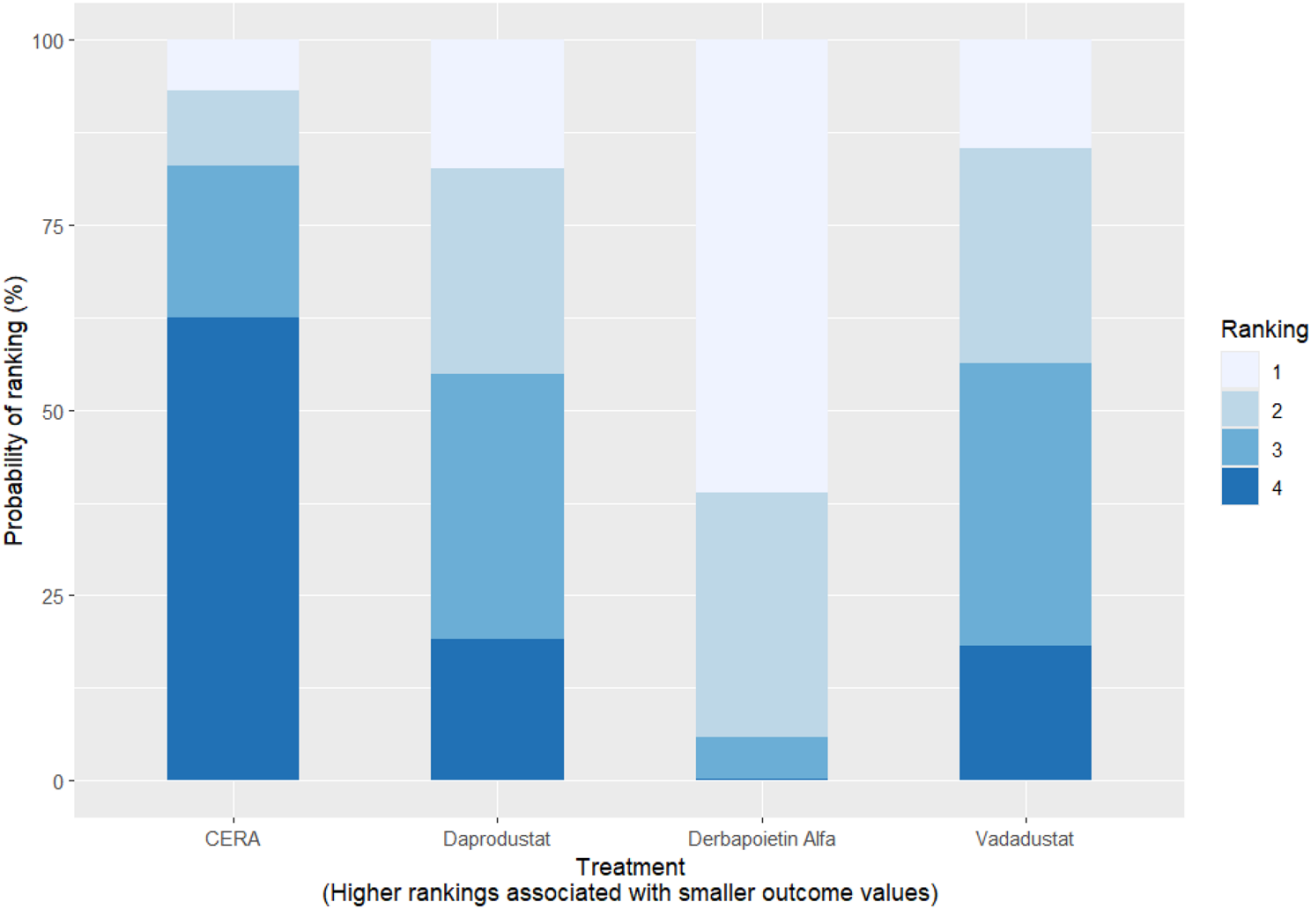
Rankogram

**Fig. 5I:**
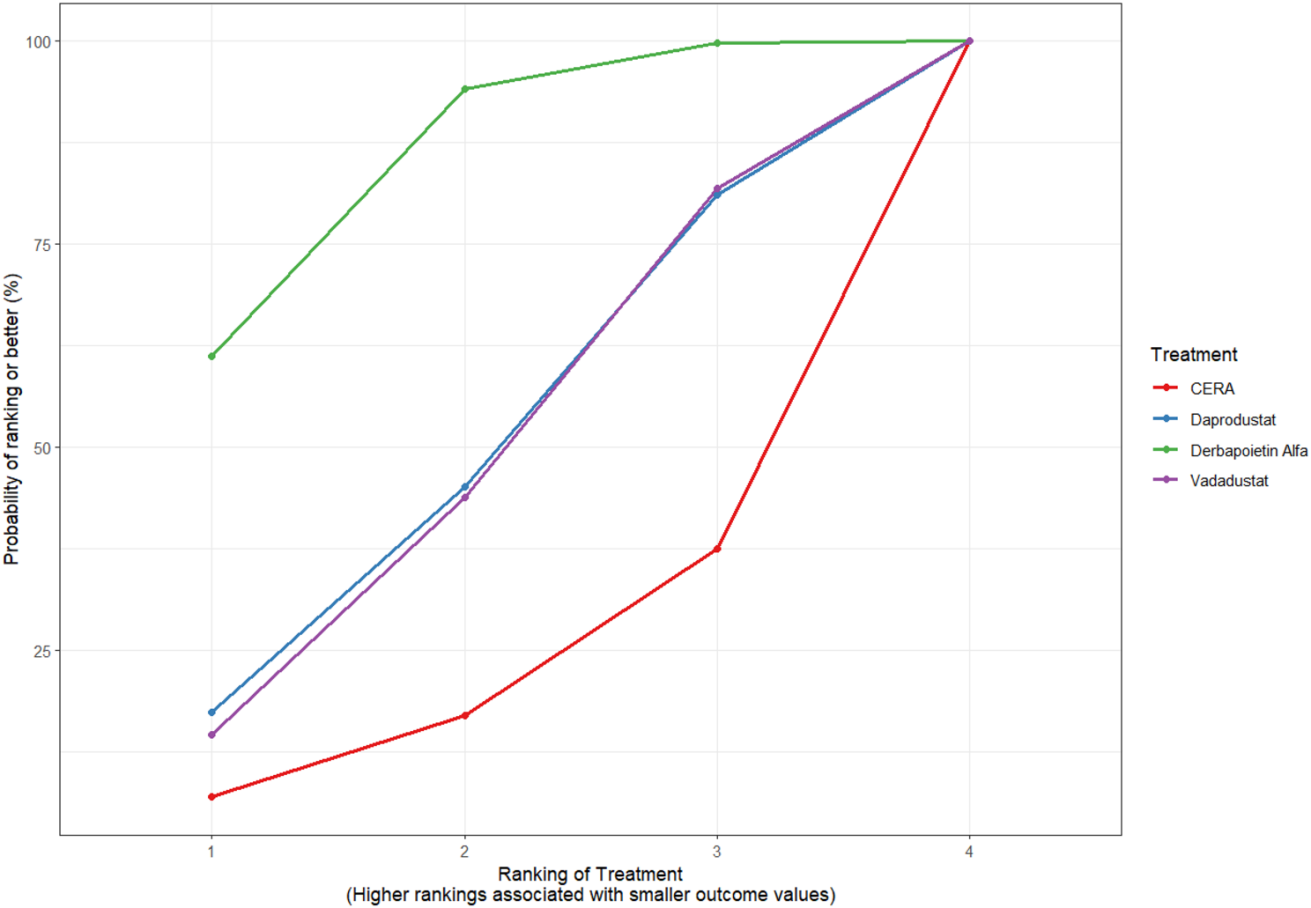
SUCRA plot

#### Thrombotic events

Thrombotic events were rare but analyzed across all treatments. Daprodustat was associated with a relatively favorable profile for thrombovascular events, achieving a SUCRA value of 1.00. CERA and Roxadustat followed closely behind. Risk estimates indicated that Roxadustat compared to traditional ESAs had an OR of 1.08 (95% CrI: 0.63 to 1.87), and Daprodustat had an OR of 0.96 (95% CrI: 0.52 to 1.77), neither reaching statistical significance. These results suggest limited differences in thrombovascular event rates across interventions as shown in Fig. 6 (A-I)

**Fig. 6A:**
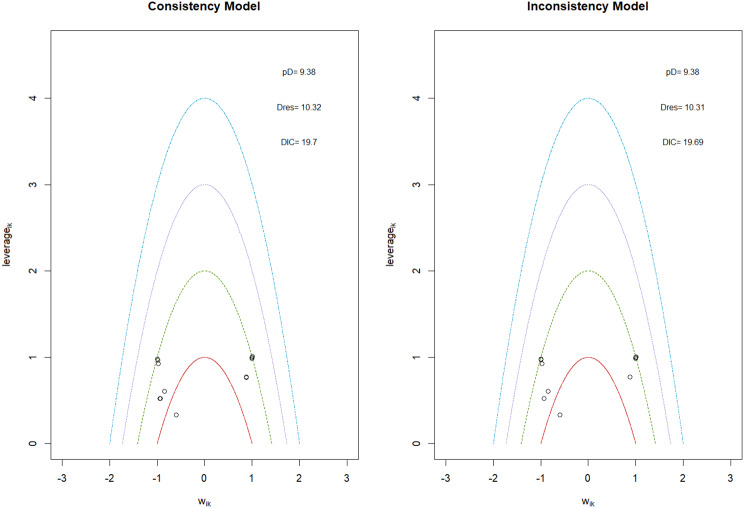
Consistency vs inconsistency plot

**Fig. 6B:**
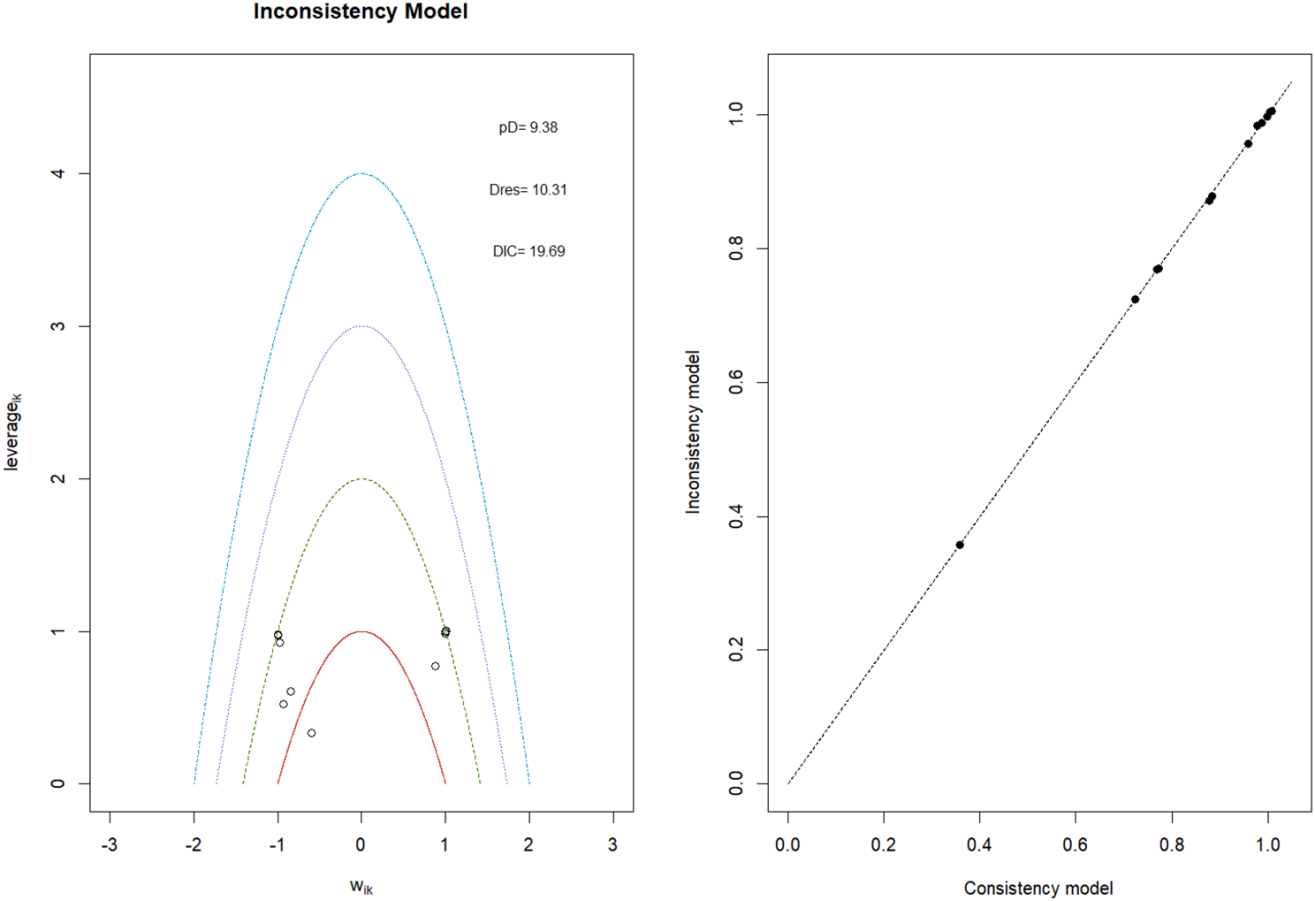
Inconsistency model

**Fig. 6C:**
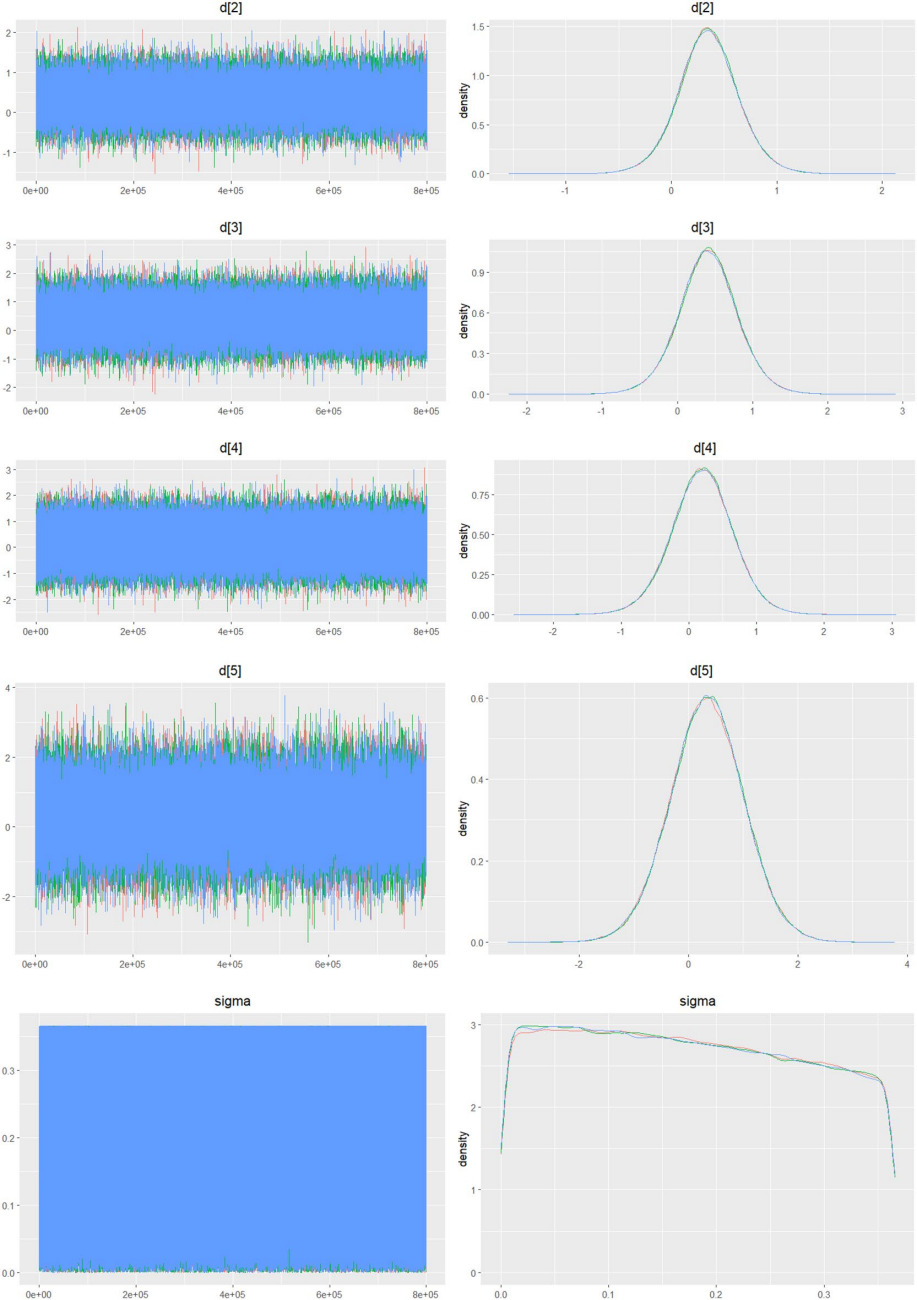
Density and trace plots

**Fig. 6D:**
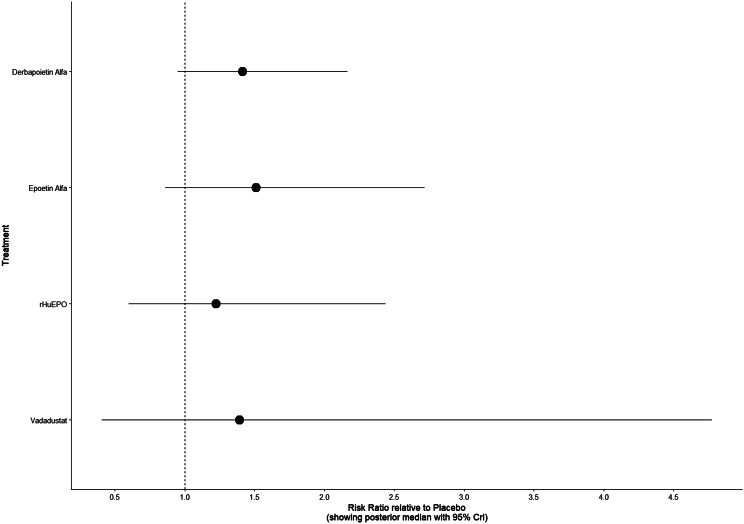
Forest plot

**Fig. 6E:**
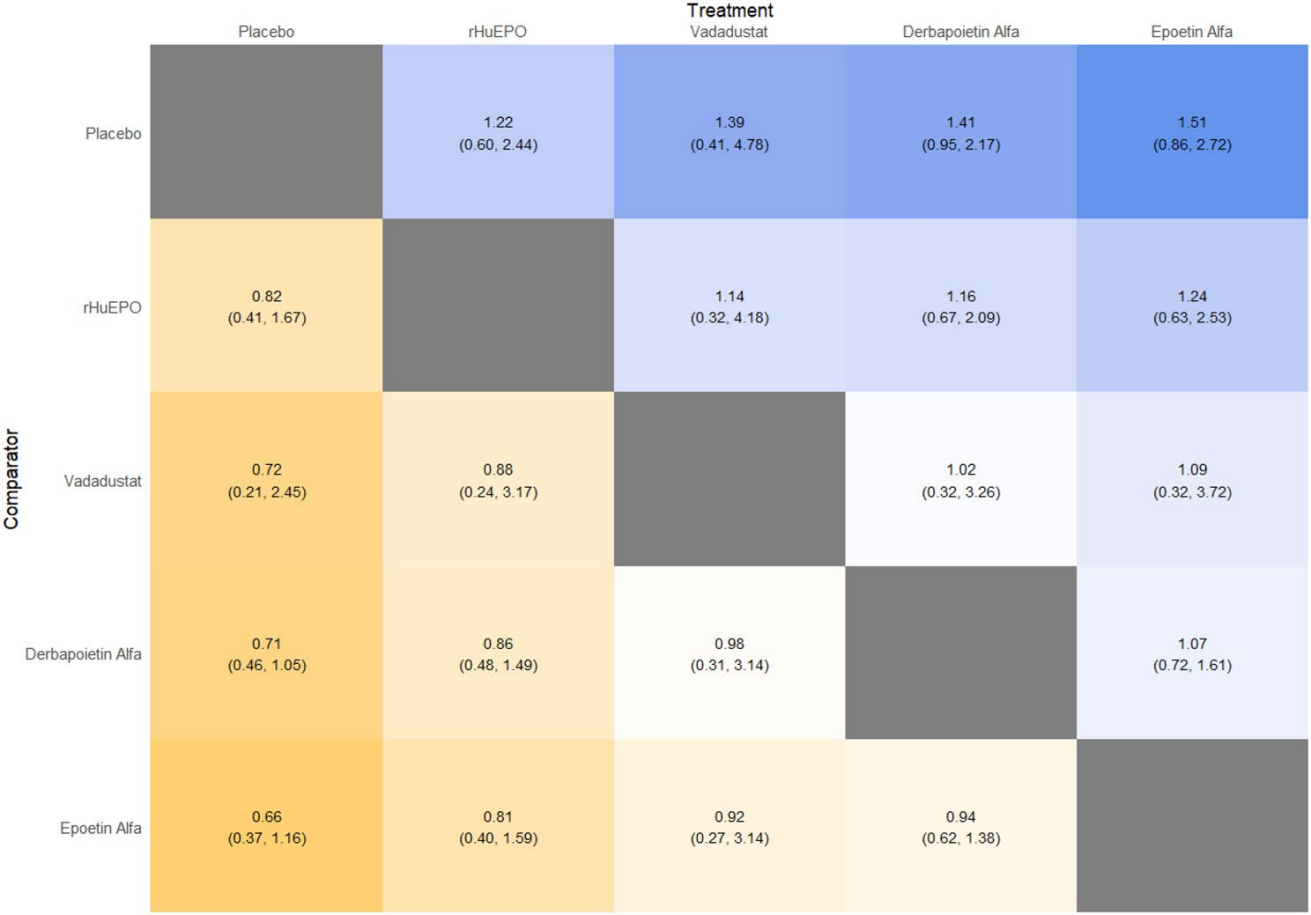
League table

**Fig. 6F:**
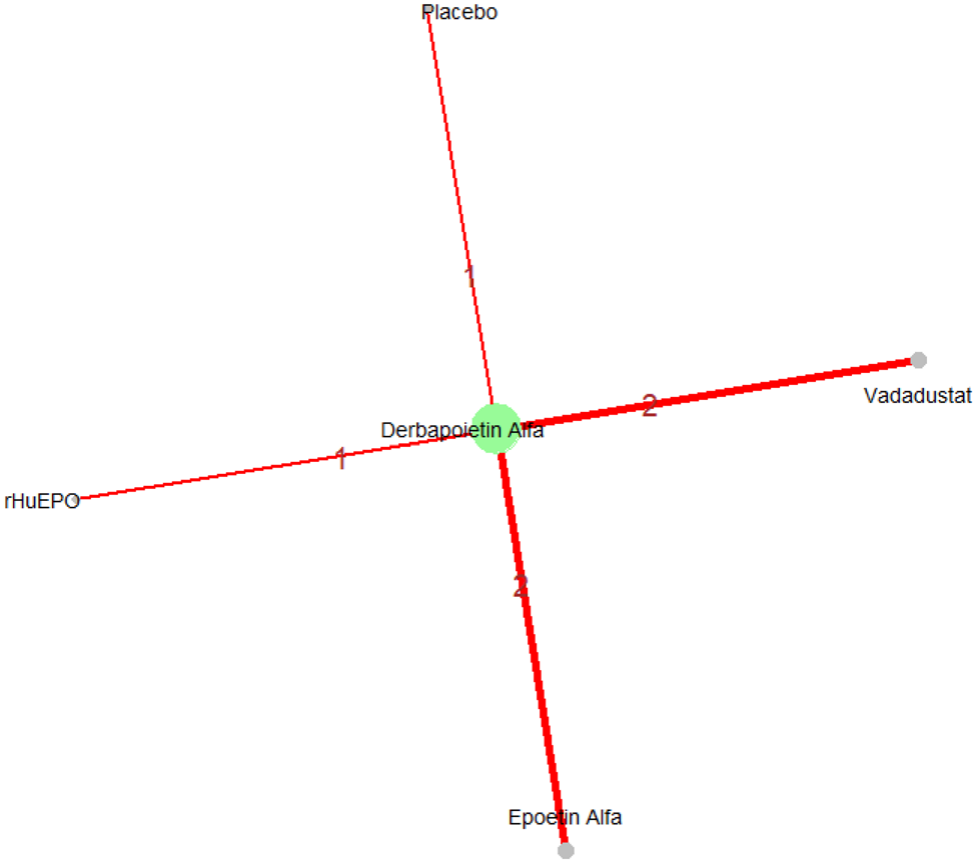
Network geometry

**Fig. 6G:**
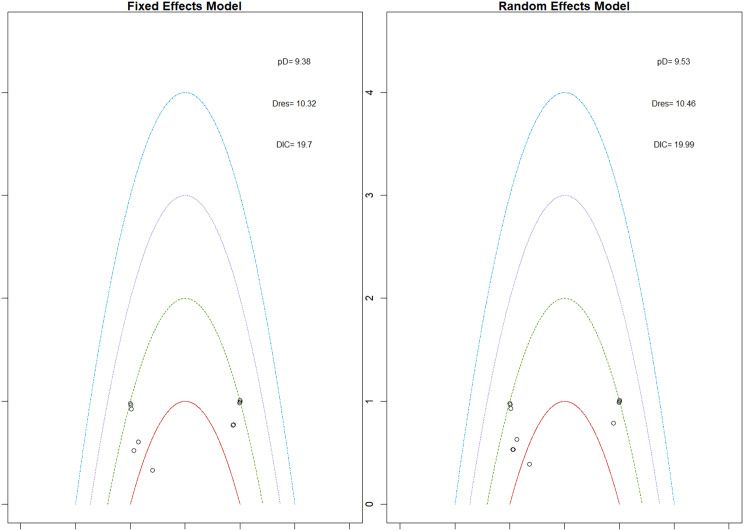
Rainbow plot

**Fig. 6H:**
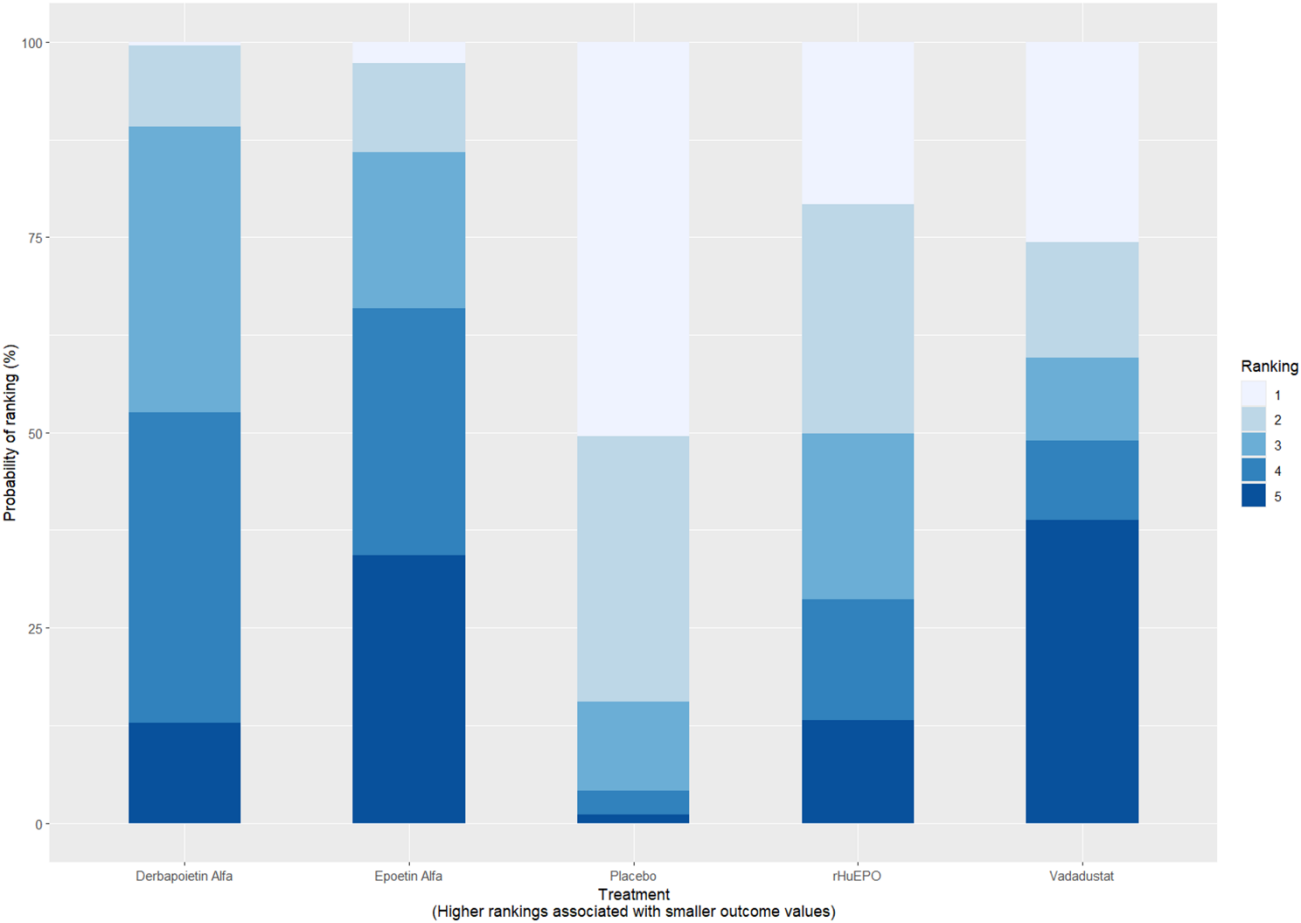
Rankogram

**Fig. 6I:**
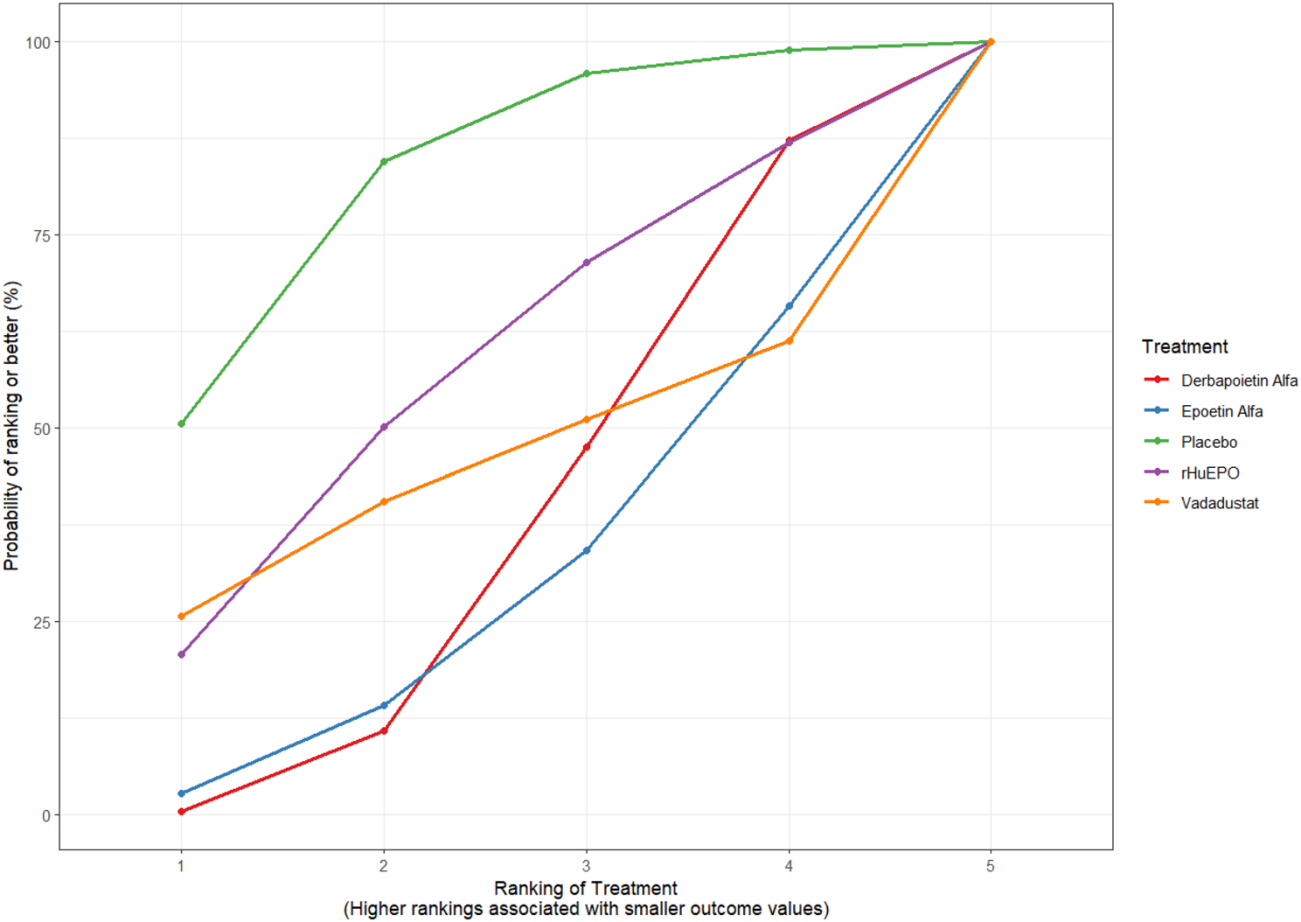
SUCRA plot

#### Hypertension

Hypertension incidence was commonly reported, especially among patients receiving traditional ESAs. Daprodustat-treated patients had a hypertension incidence of 12.3%, compared to 15.1% among darbepoetin users, corresponding to an OR of 0.79 (95% CrI: 0.59 to 1.04). Roxadustat showed a similar trend toward lower hypertension incidence. However, no statistically significant differences between treatments were observed, and event rates remained numerically lower among patients receiving HIF prolyl hydroxylase inhibitors compared to ESAs as shown in Fig. 7 (A-I)

**Fig. 7A:**
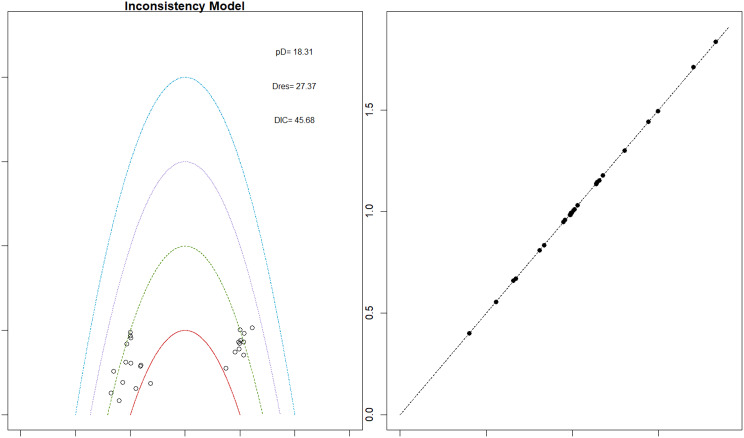
Consistency comparison plot

**Fig. 7B:**
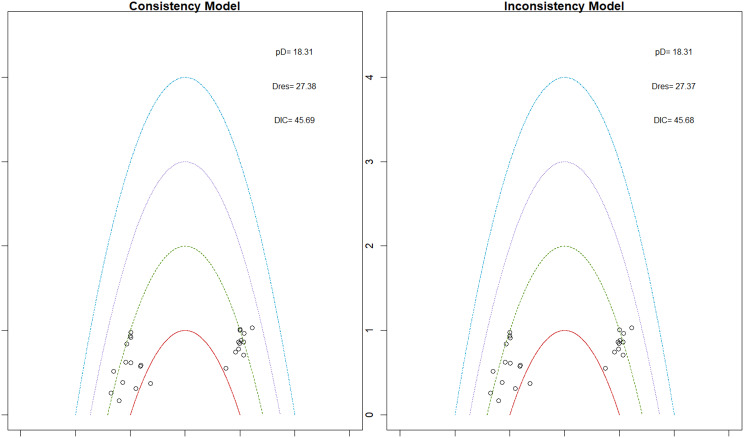
Consistency vs inconsistency plot

**Fig. 7C:**
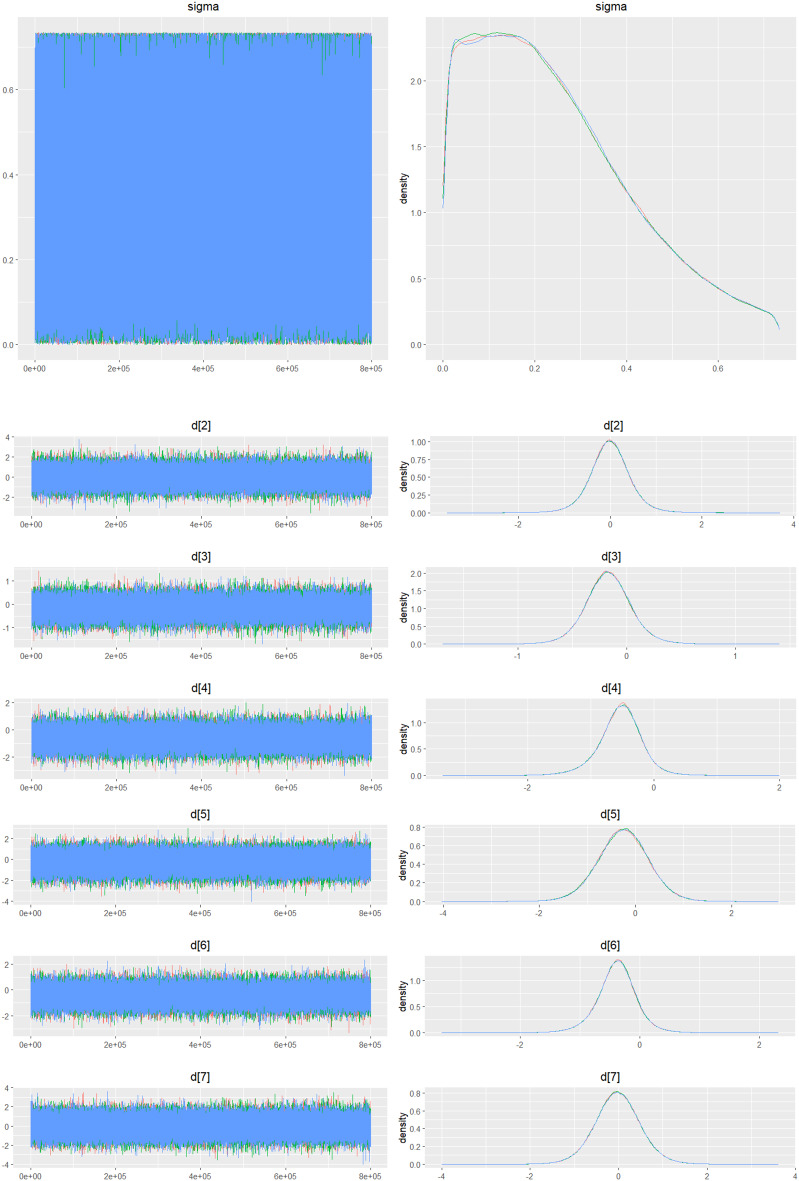
Density and trace plots

**Fig. 7D:**
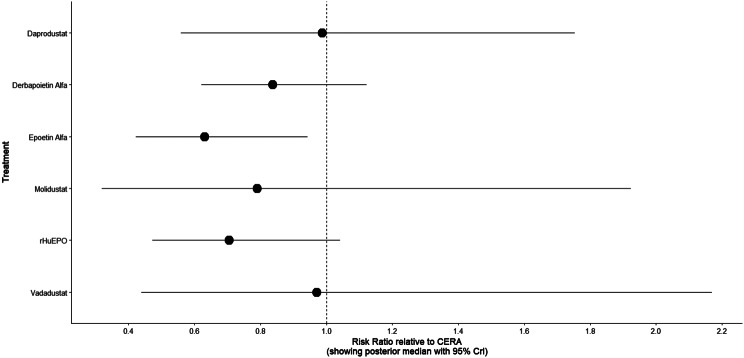
Forest plot

**Fig. 7E:**
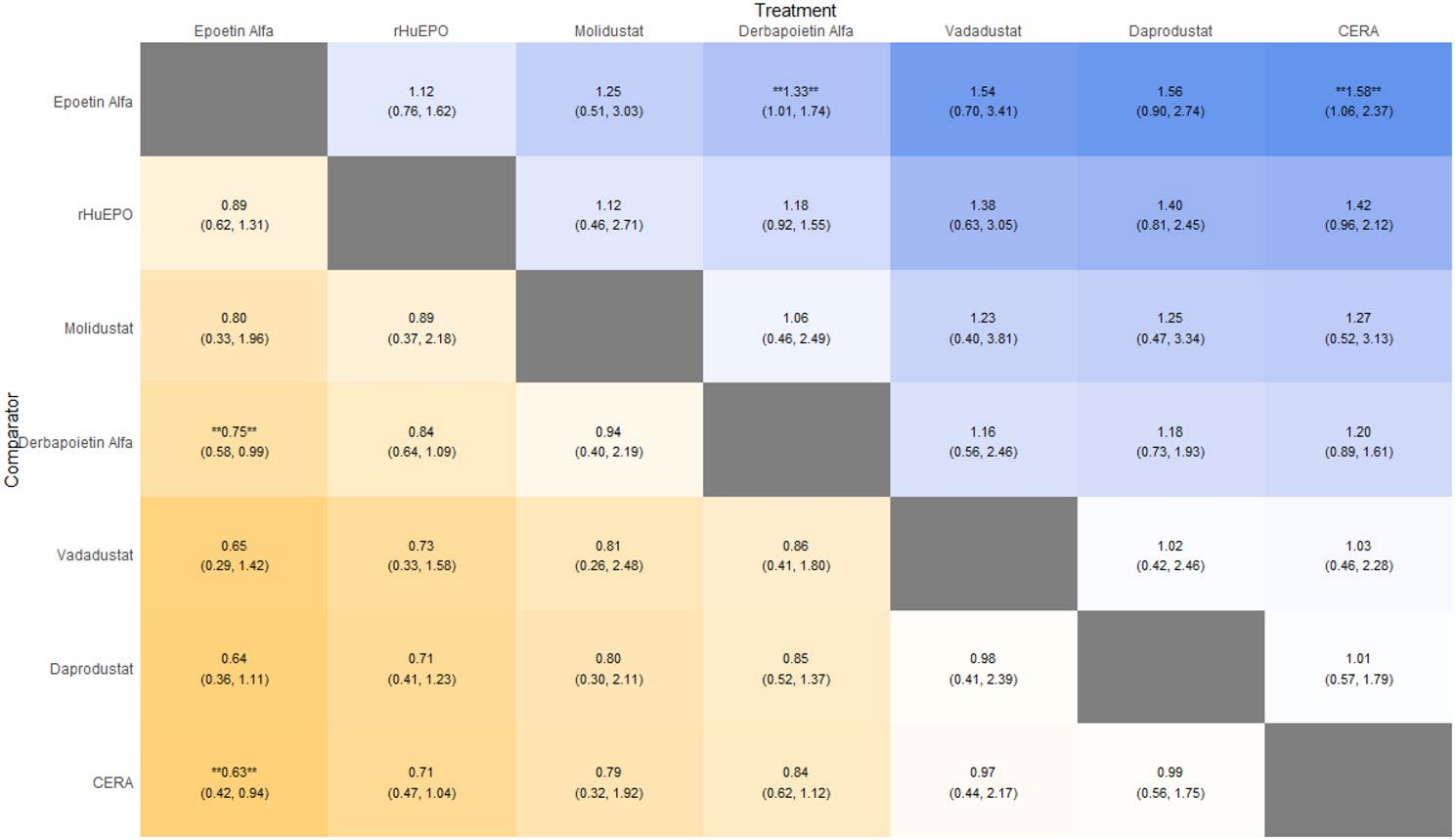
League table

**Fig. 7F:**
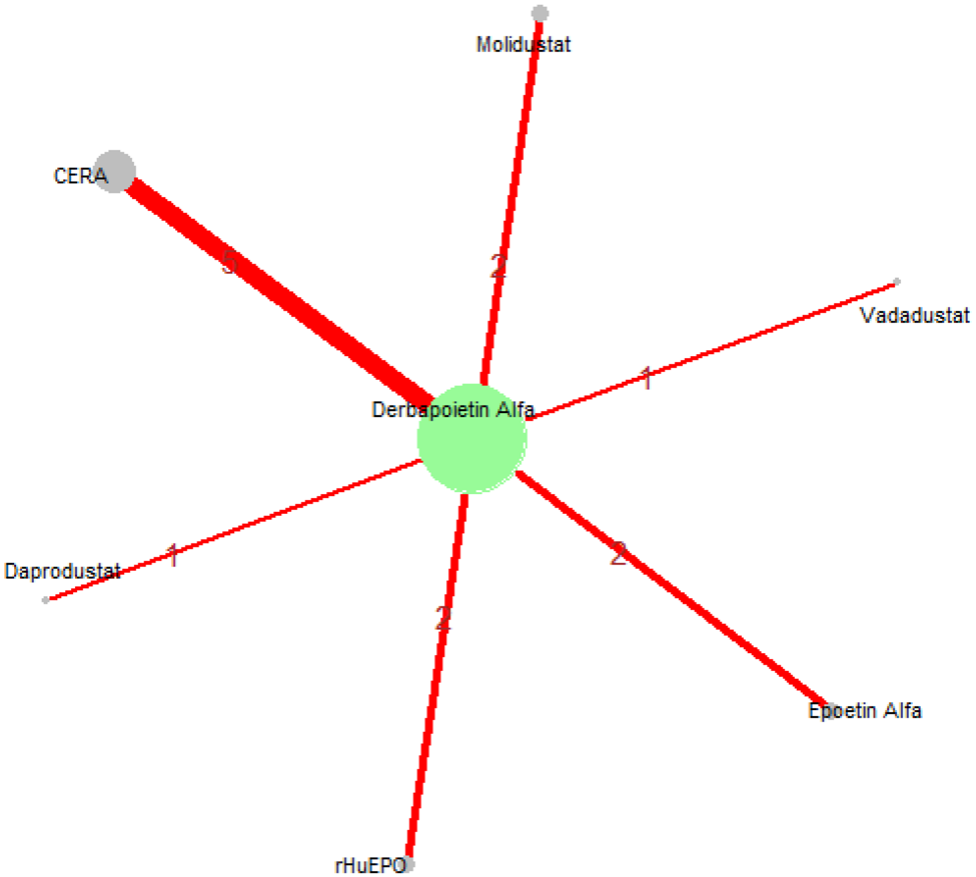
Network geometry

**Fig. 7G:**
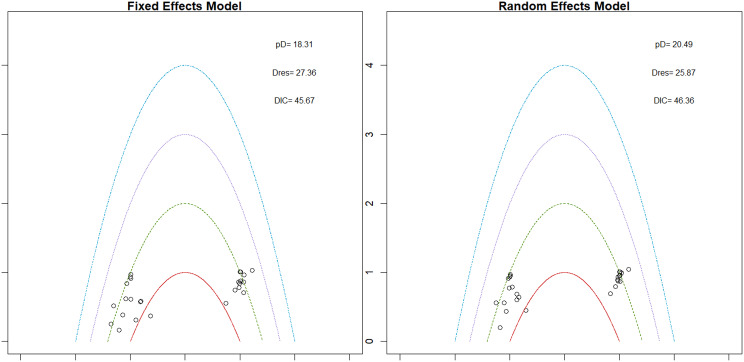
Rainbow plot

**Fig. 7H:**
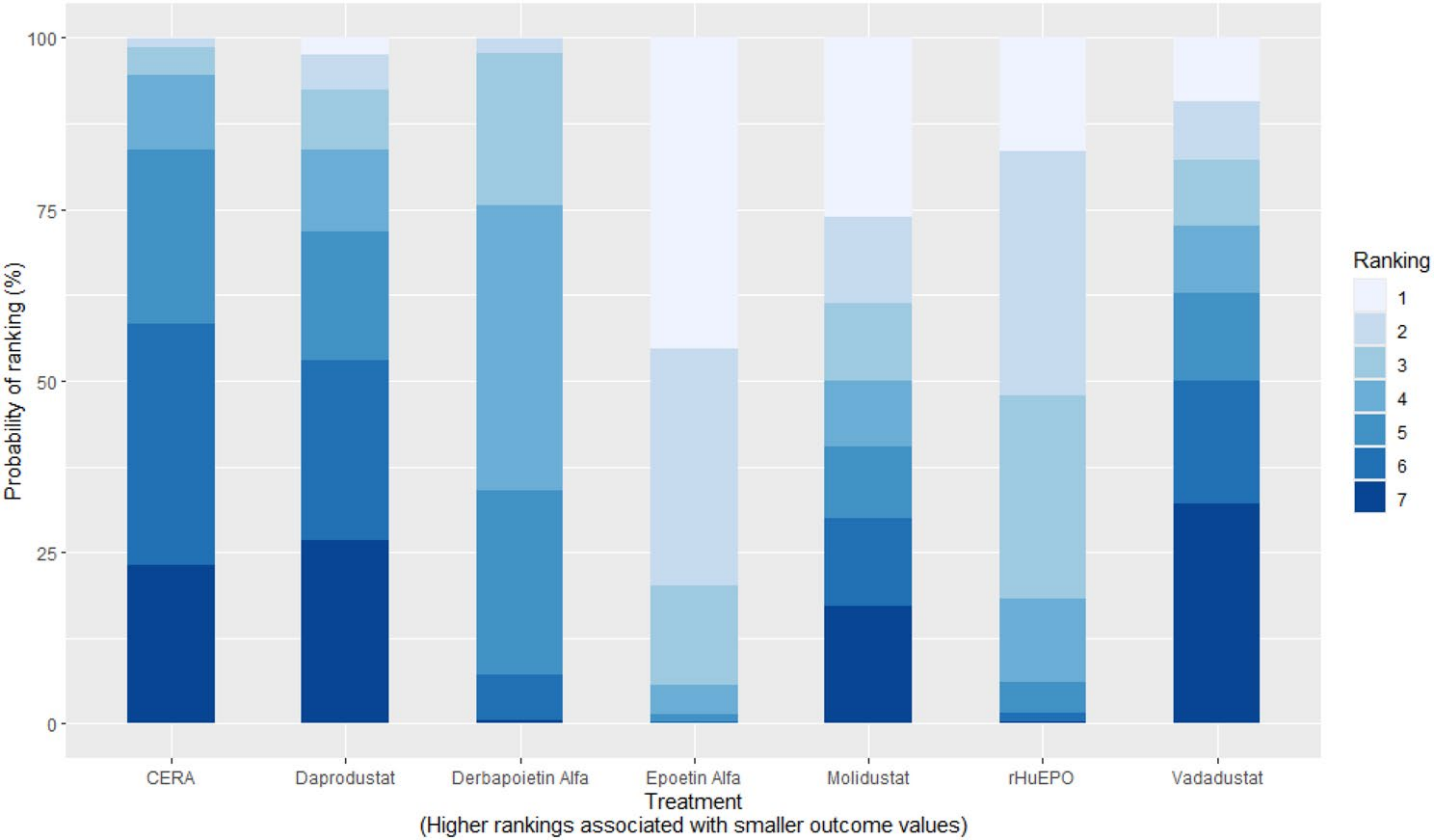
Rankogram

**Fig. 7I:**
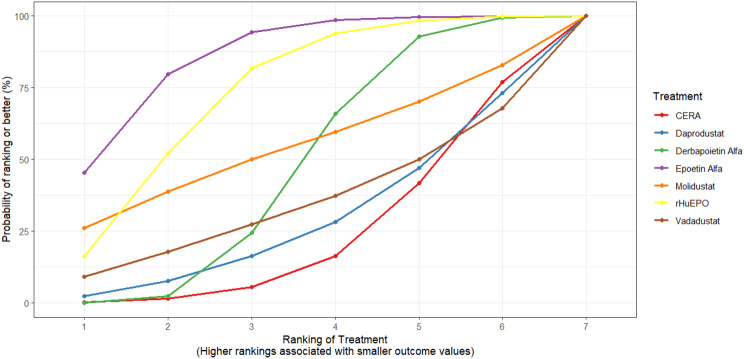
SUCRA plot

#### Gastrointestinal adverse events

Gastrointestinal tolerability was assessed across trials. Diarrhea was the most frequent gastrointestinal adverse event, particularly associated with Roxadustat. In two studies, diarrhea occurred in 14.2% of patients treated with Roxadustat versus 8.1% among those receiving darbepoetin, yielding an OR of 1.88 (95% CrI: 1.10 to 3.22). In contrast, Daprodustat and Vadadustat were not associated with an increased risk of gastrointestinal adverse events relative to comparators, indicating a favorable overall gastrointestinal safety profile for these agents as shown in Fig. 8 (A-I)

**Fig. 8A:**
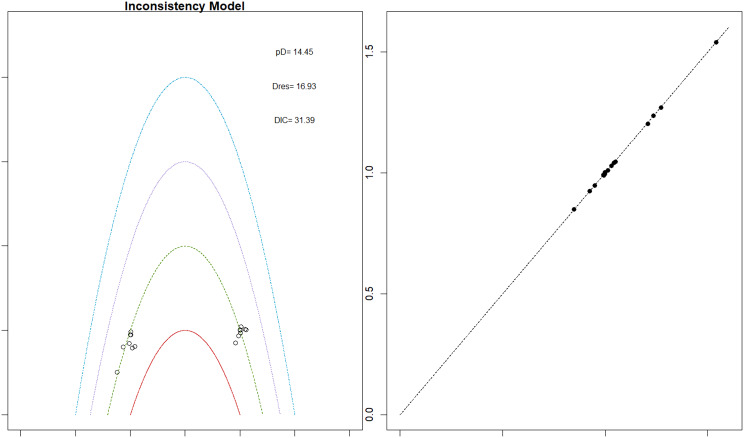
Consistency comparison plot

**Fig. 8B:**
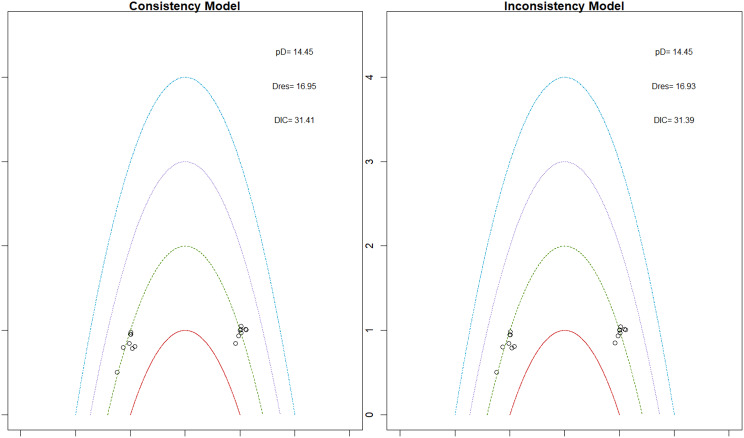
Consistency vs inconsistency Plot

**Fig. 8C:**
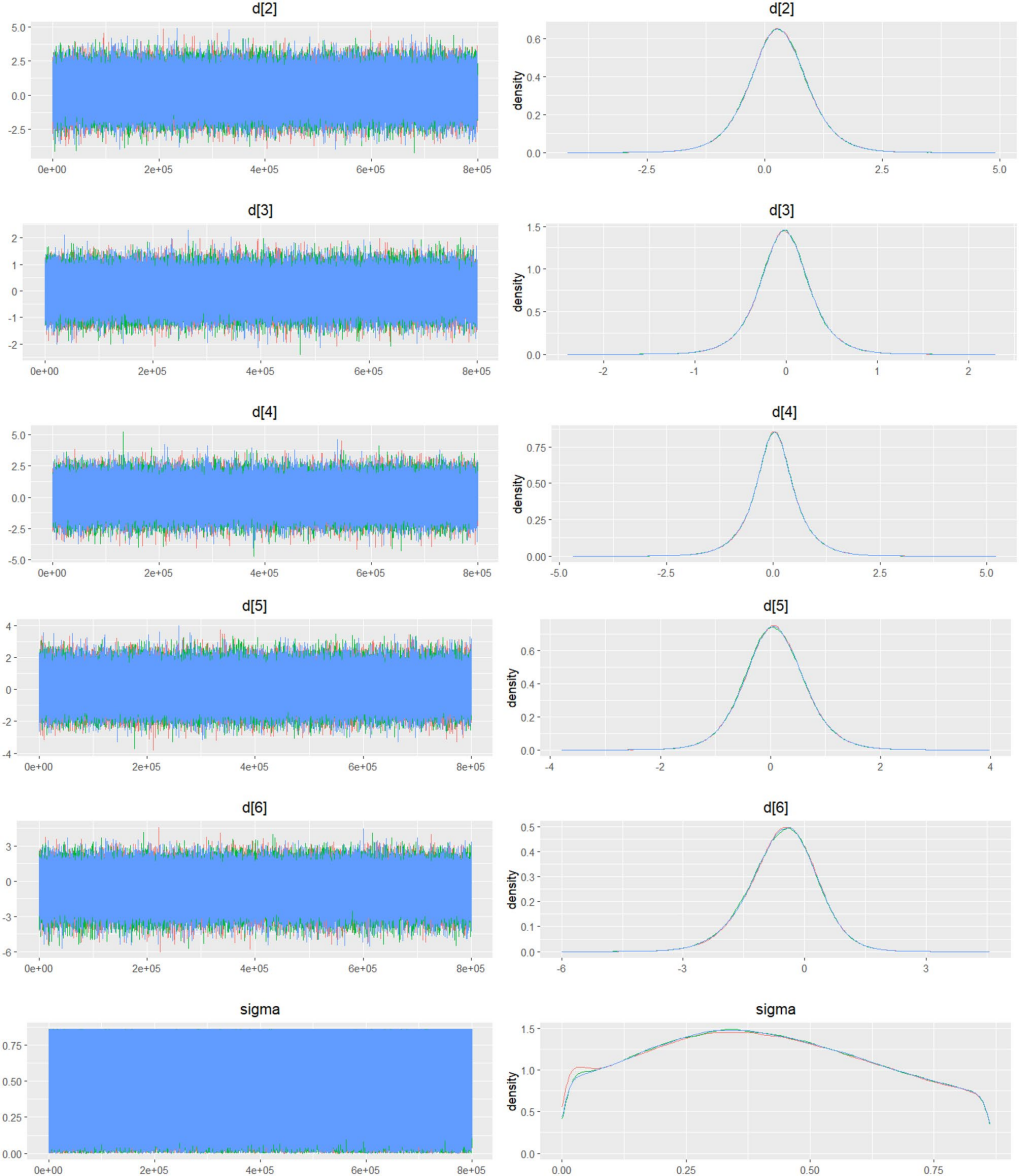
Density and trace plot

**Fig. 8D:**
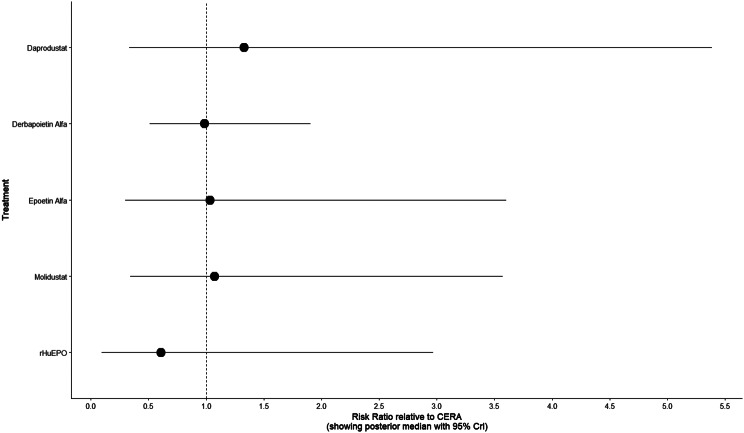
Forest plot

**Fig. 8E:**
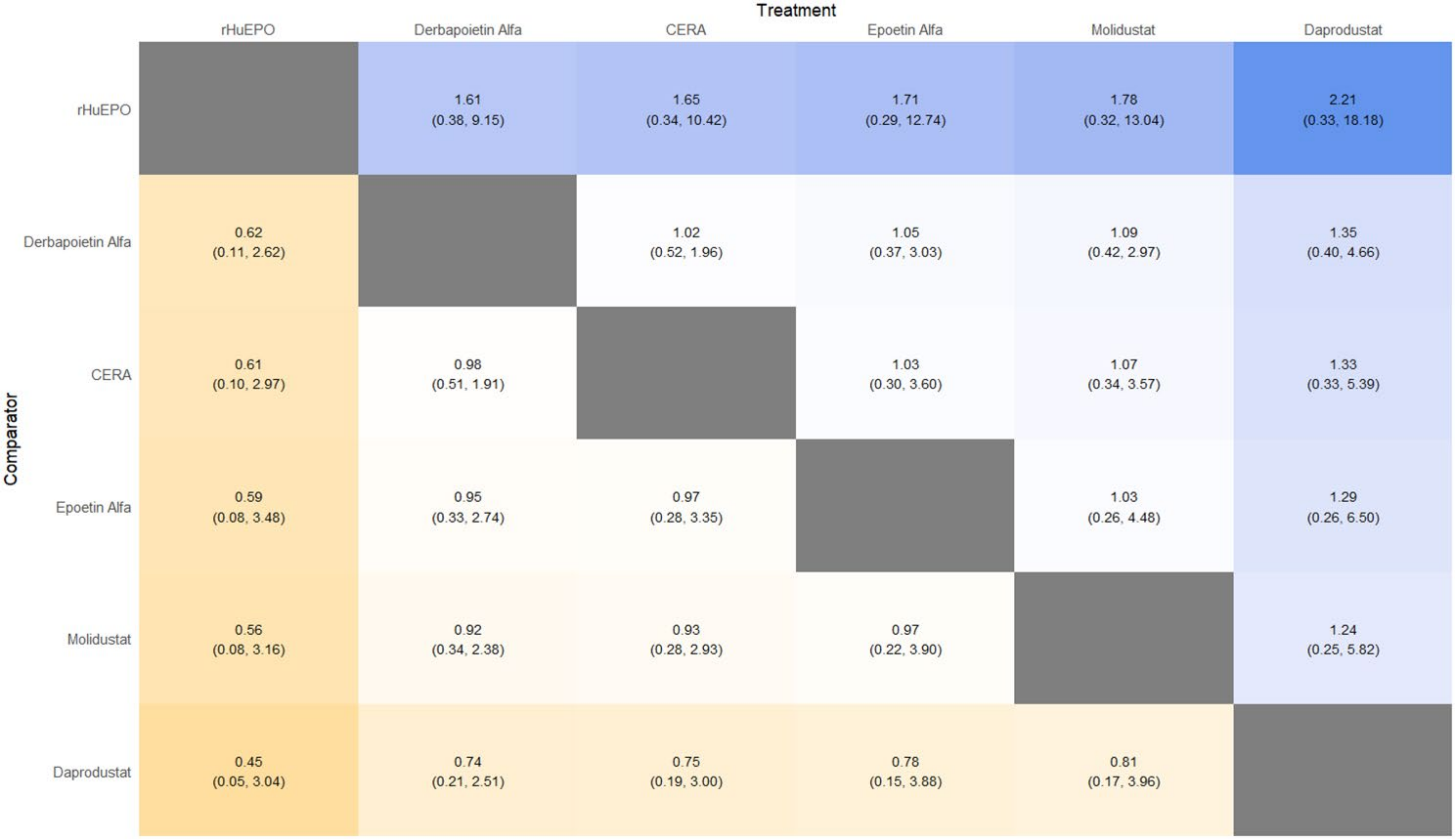
League table

**Fig. 8F:**
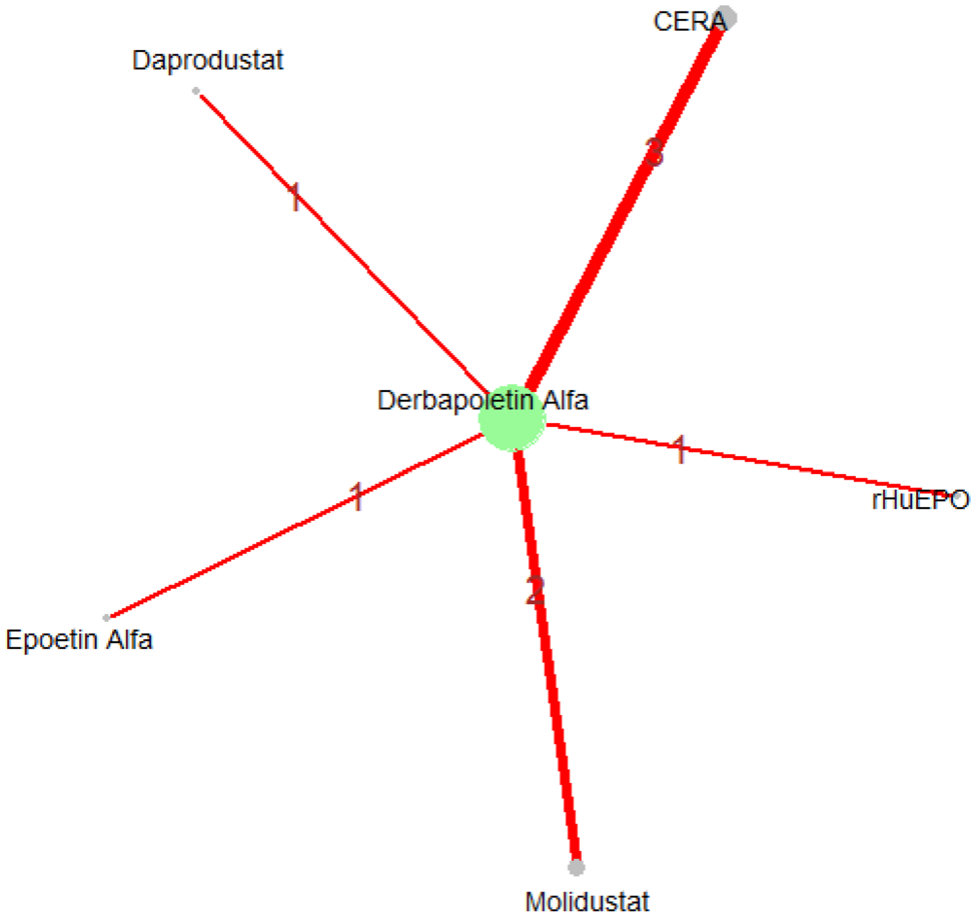
Network geometry

**Fig. 8G:**
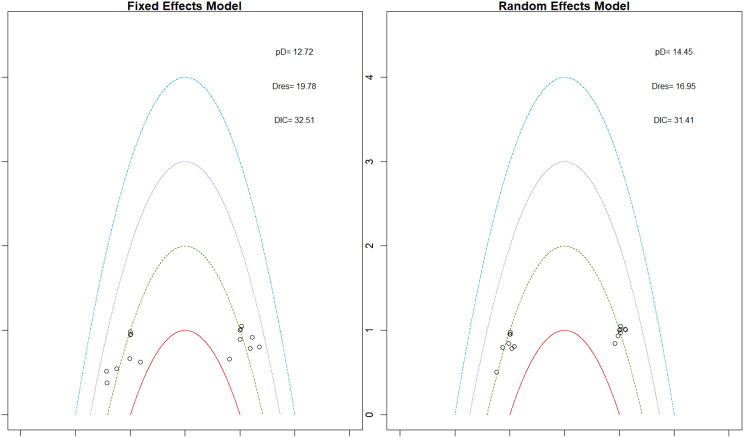
Rainbow plot

**Fig. 8H:**
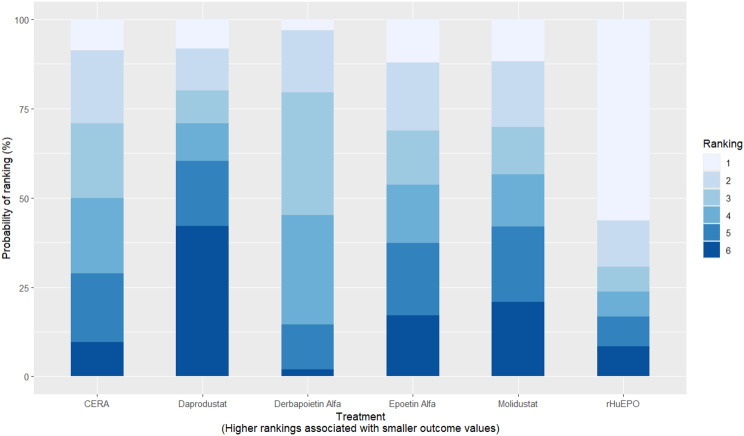
Rankogram

**Fig. 8I:**
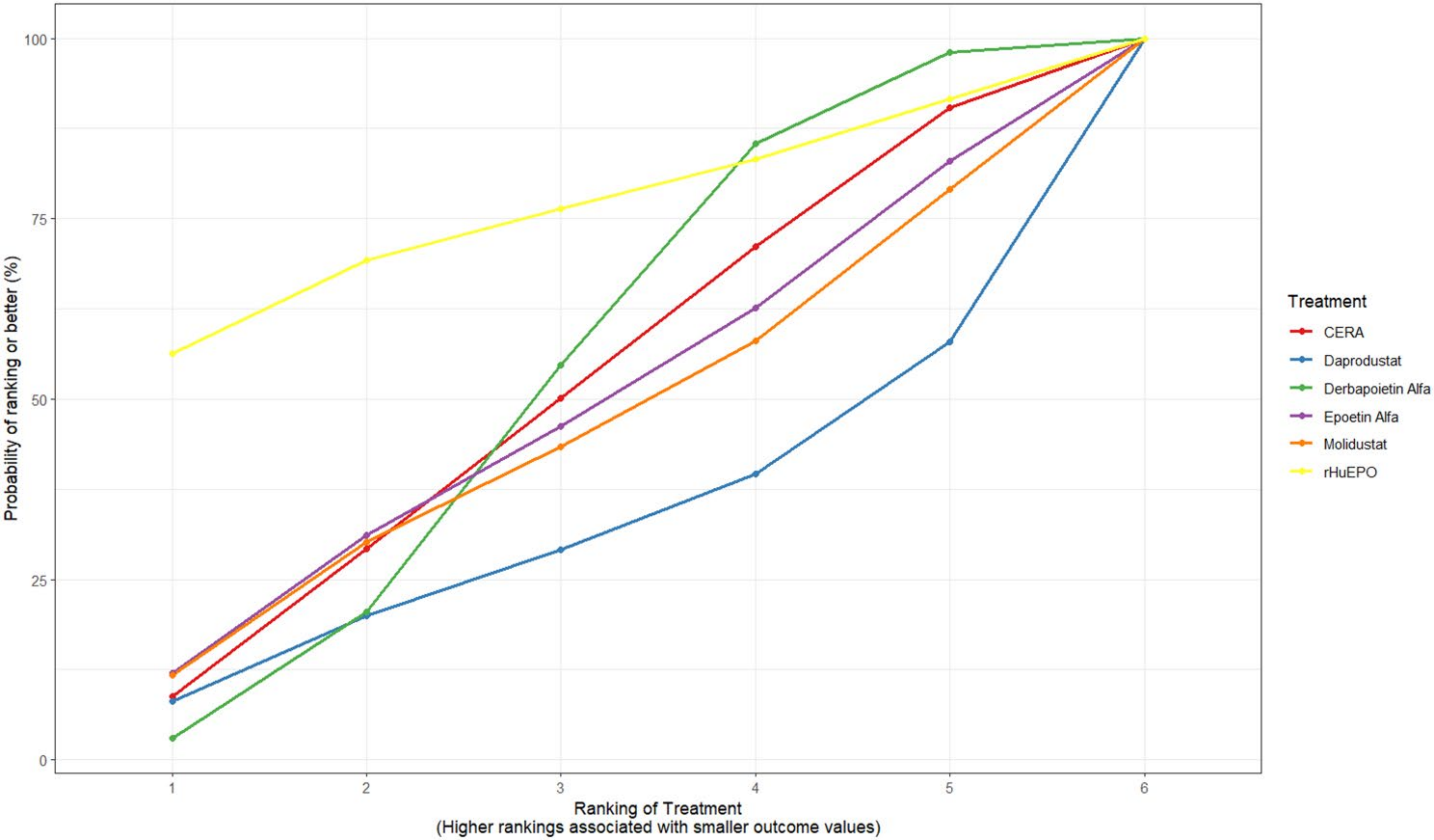
SUCRA plot

#### Diabetes-related adverse events

Diabetes-related adverse events, including the development of new-onset diabetes or worsening glycemic control, were infrequently reported and did not differ significantly between treatment groups. Due to the low event rates and broad credible intervals, no firm conclusions regarding differential diabetes risk across interventions could be drawn. The results are shown in Fig. 9 (A-I)

**Fig. 9A:**
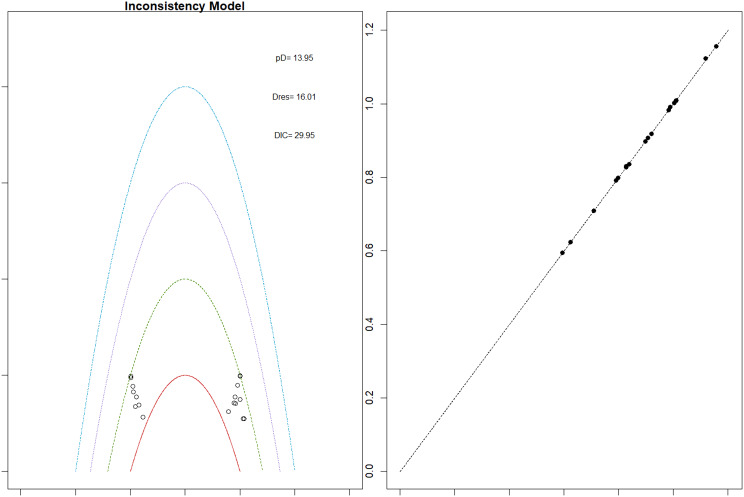
Consistency comparison plot

**Fig. 9B:**
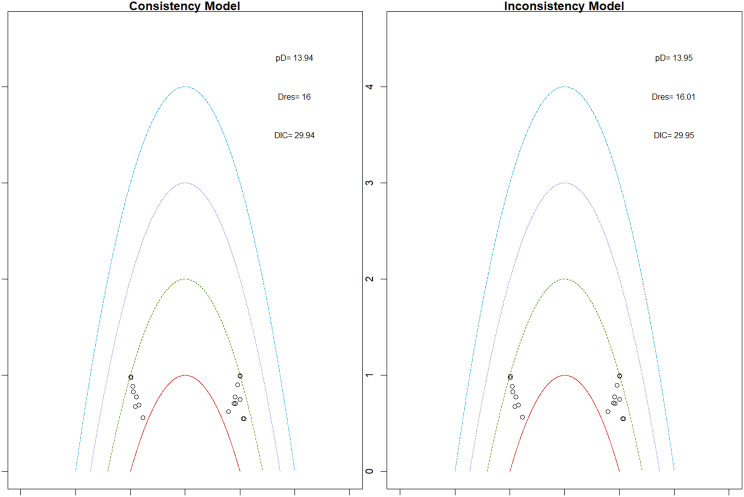
Consistency vs inconsistency plot

**Fig. 9C:**
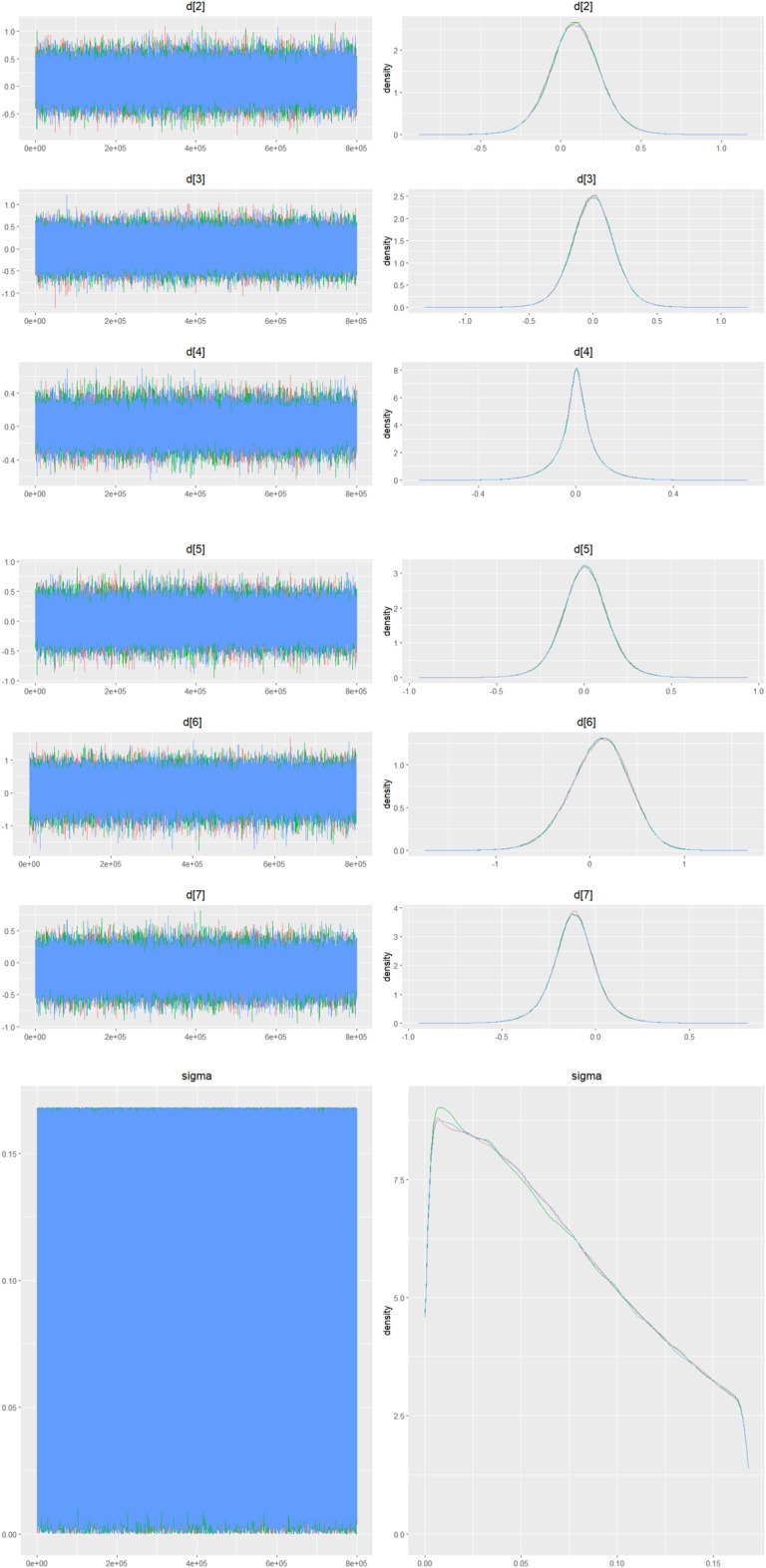
Density and trace plots

**Fig. 9D:**
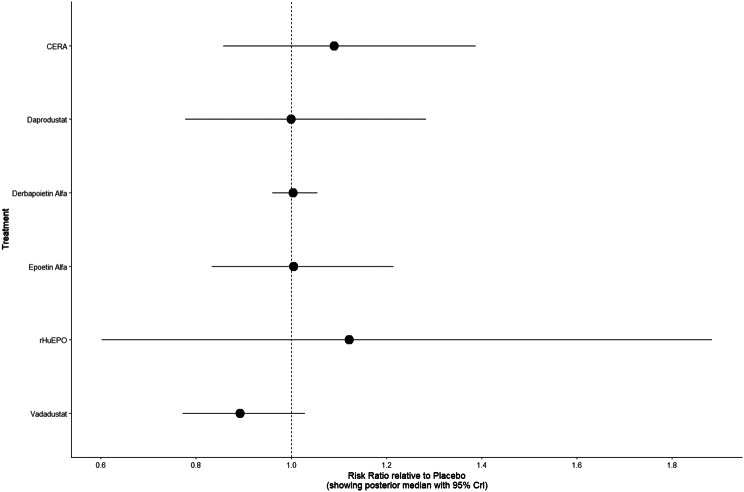
Forest plot

**Fig. 9E:**
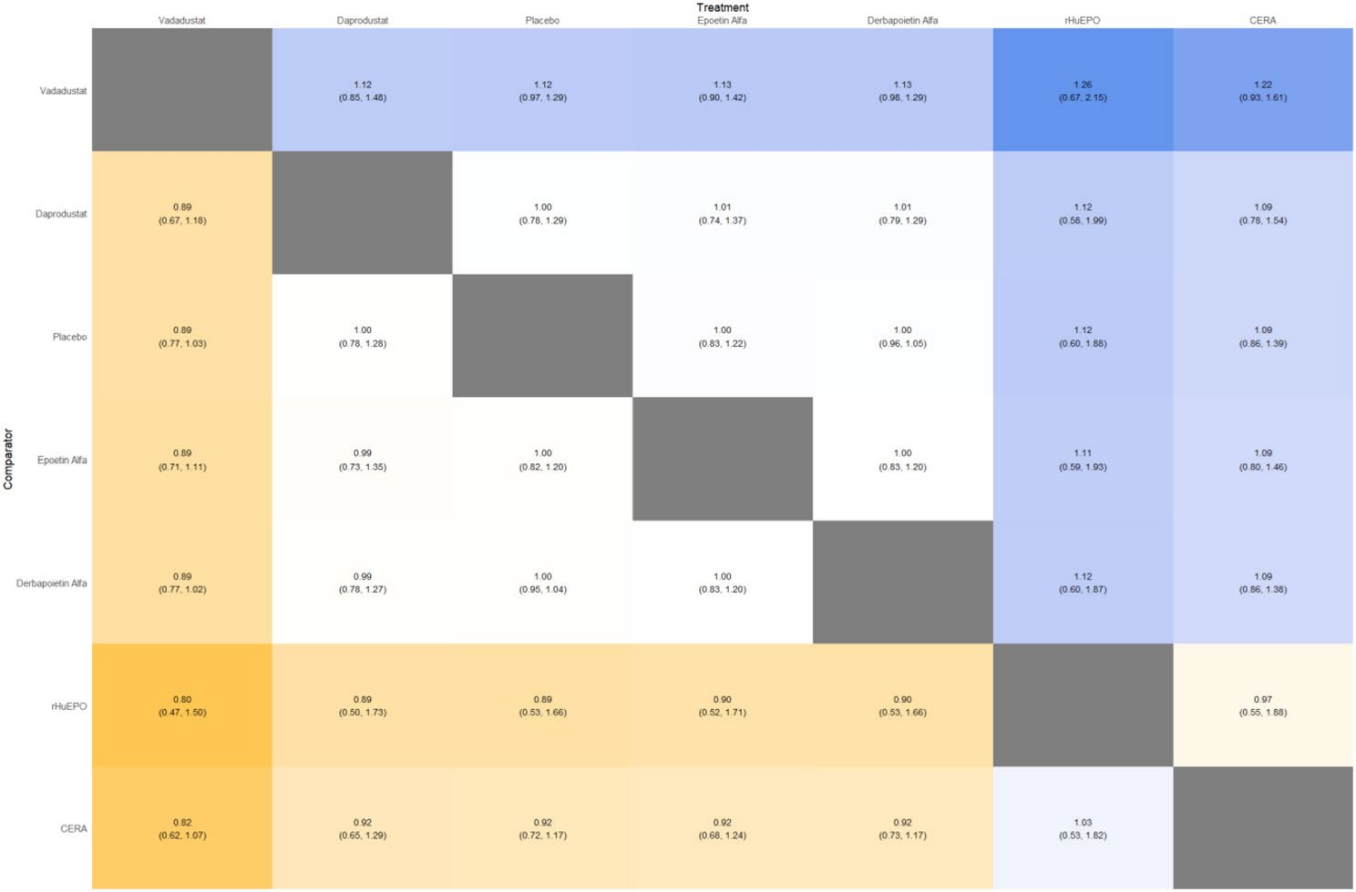
League table

**Fig. 9F:**
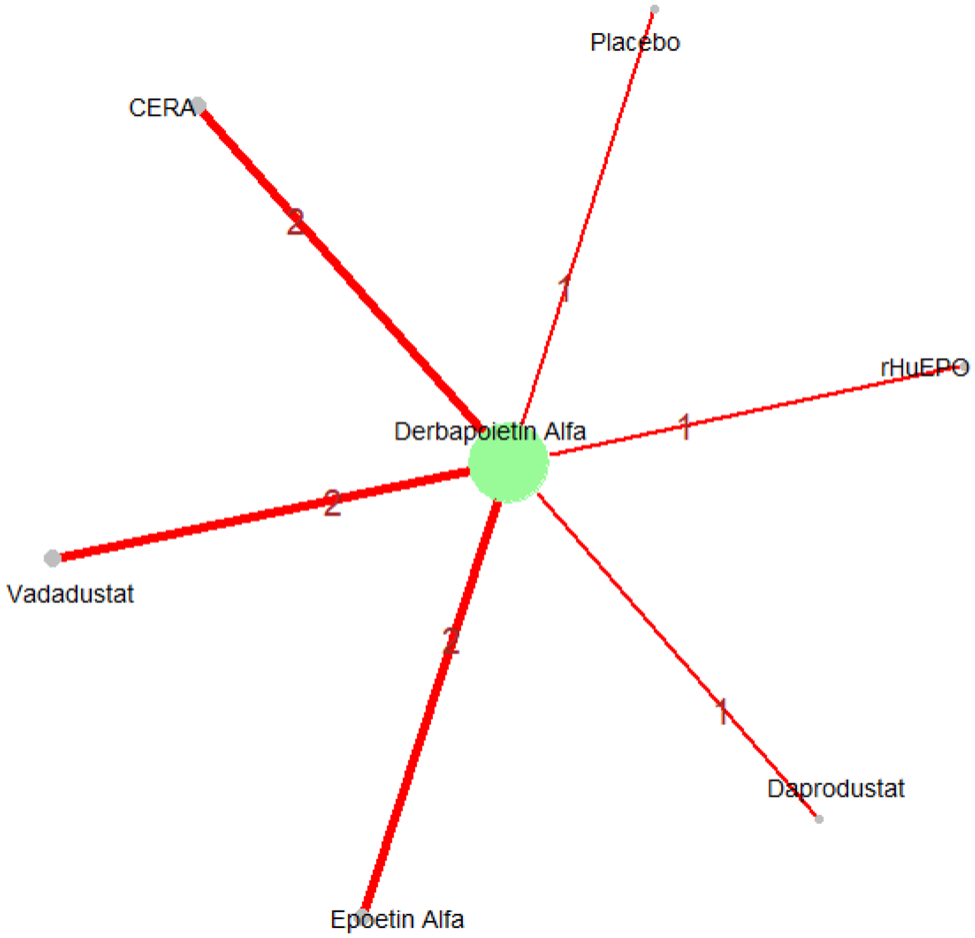
Network geometry

**Fig. 9G:**
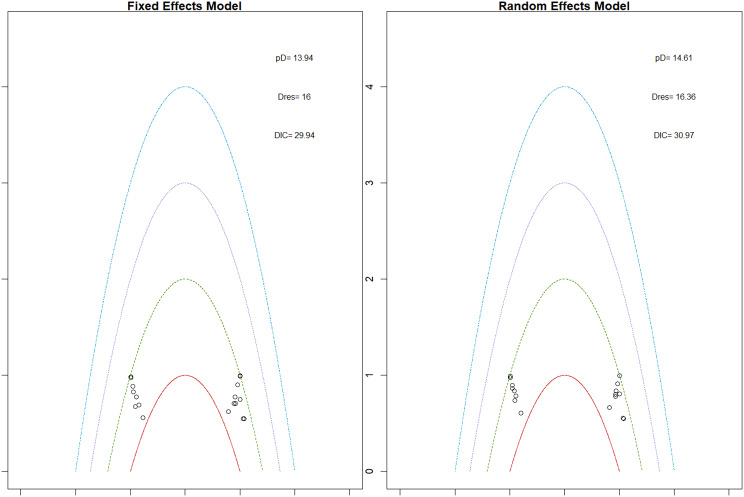
Rainbow plot

**Fig. 9H:**
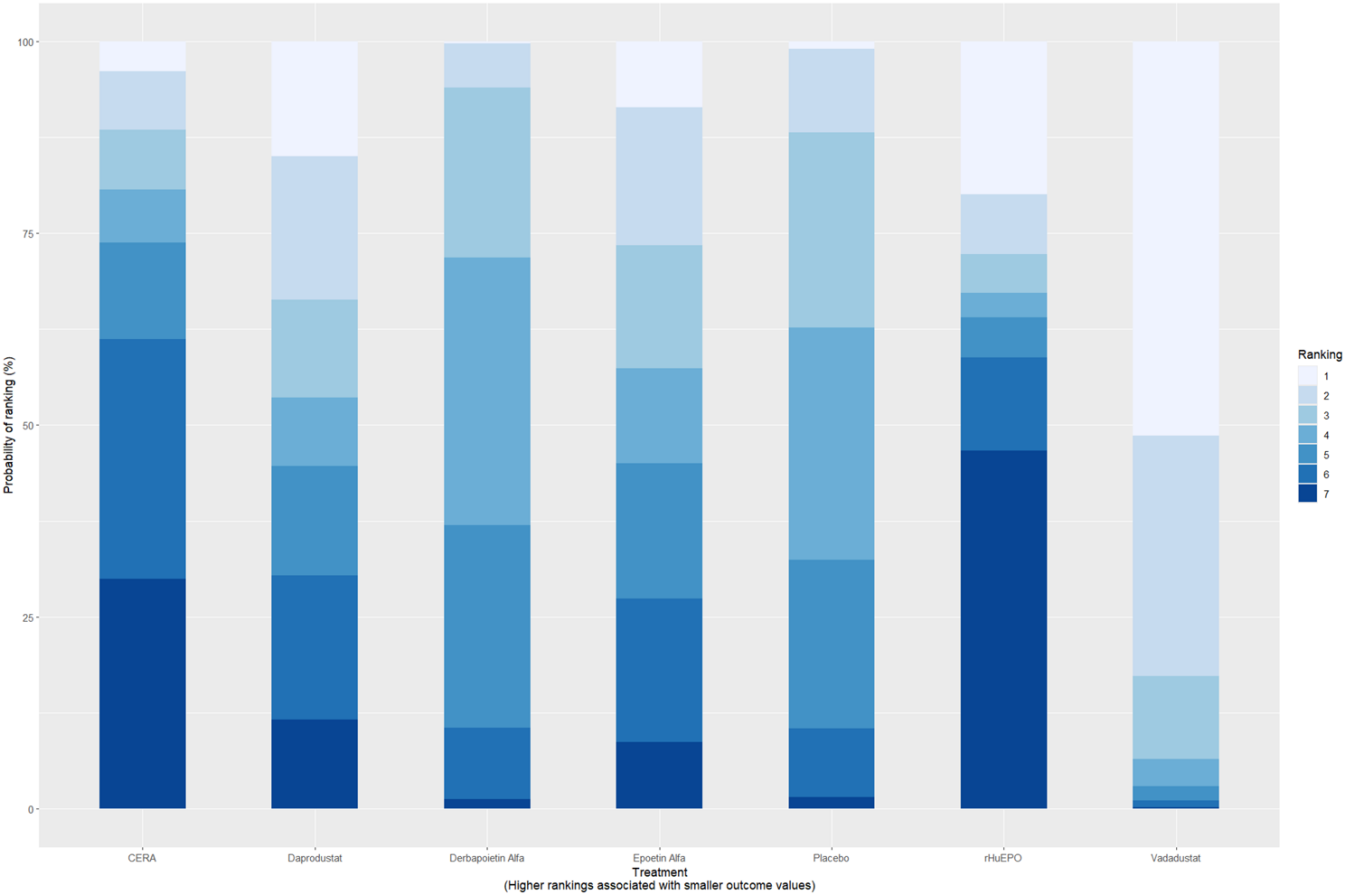
Rankogram

**Fig. 9I:**
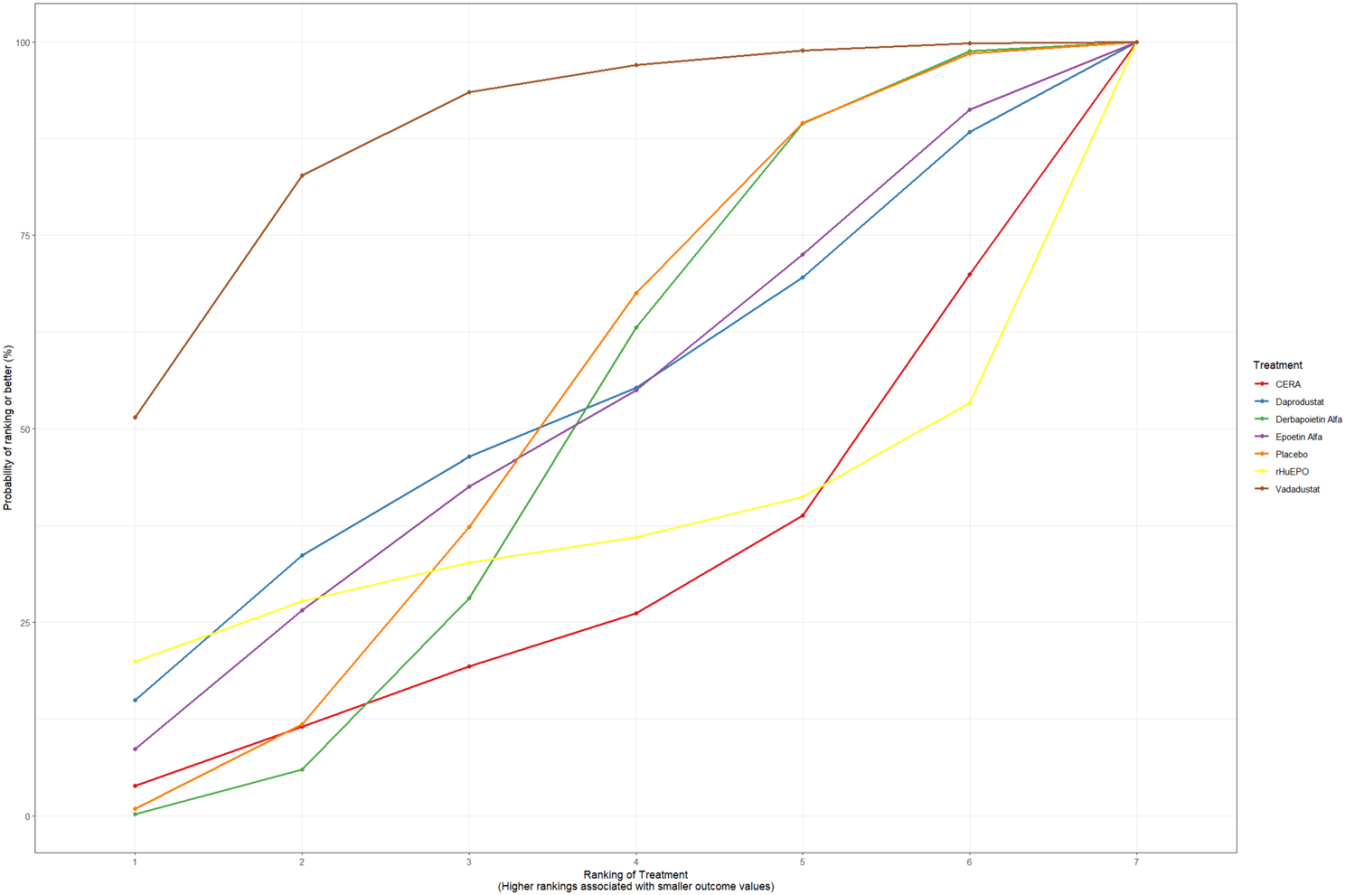
SUCRA plot

### Comparative treatment ranking

Based on the collective analysis of efficacy and safety endpoints, Methoxy polyethylene glycol-epoetin beta ranked highest for efficacy in raising hemoglobin levels, although data on its cardiovascular and thrombovascular safety outcomes were limited. Molidustat, despite lower hemoglobin efficacy, demonstrated outstanding cardiovascular safety, making it an attractive option for patients at high cardiovascular risk. Daprodustat exhibited moderate efficacy with a relatively balanced safety profile but carried potential concerns regarding gastrointestinal adverse events in comparison to other agents. CERA achieved a moderate balance between efficacy and safety. Roxadustat and Vadadustat demonstrated favorable hemoglobin and cardiovascular outcomes in selected comparisons but with considerable uncertainty due to wide credible intervals. Results for efficacy are summarized in Table 3

**Table 3:** Comparative efficacy of ESAs

Agent	Rank for Hgb Rise+	Magnitude of Hb change vs Baseline*	Comments
Darbepoetin Alpha	-	Comparator	Std
Molidustat	4	Lowest efficacy	Safe CV profile
Roxadustat	2–3	Equivalent or Higher vs Darbepoetin	Higher GI AEs
Daprodustat	2	Moderate increase > darbepoetin	Similar mortality and CV event rates
Methyl-PEG-epoetin beta	1	Largest increase in Hgb among all	Long-acting

+ Lower rank number = greater hemoglobin-raising efficacy

Comparison only, not an exact quantitative ranking

## Discussion

This network meta-analysis studied the efficacy and safety outcomes of conventional erythropoiesis-stimulating agents (ESAs) with newer therapies of hypoxia-inducible factor prolyl hydroxylase inhibitors (HIF-PHIs) for managing anemia in chronic kidney disease (CKD). Our findings demonstrate that HIF-PHIs specifically in daprodustat and roxadustat show comparable or marginally better efficacy to ESAs in elevating hemoglobin (Hb) levels, with varied safety outcomes among different agents and populations.

One of the key outcomes of our analysis was the small yet statistically meaningful improvement in Hb concentration linked with daprodustat when compared to darbepoetin (mean difference: +0.15 g/dL; 95% CrI: 0.03 to 0.29), and a comparable directional pattern with roxadustat (mean difference: +0.12 g/dL; 95% CrI: −0.05 to 0.27) [14]. These results are consistent with randomized controlled trials such as ASCEND-D and ROCKIES, which demonstrated that daprodustat and roxadustat were equivalent to ESAs in attaining target Hb levels in dialysis and non-dialysis patients [15, 16]. These findings suggest HIF-PHIs as promising alternatives rather than recognizing a single ideal therapy.

Interestingly, while HIF-PHIs impacted erythropoiesis, their effect on transferrin saturation (TSAT) was less, with intergroup differences persisting non-significant (mean difference: +0.6%; 95% CrI: −1.4 to +2.5) [14]. This reflects that although HIF-PHIs may elevate endogenous erythropoietin synthesis, they do not significantly alter iron use pathways, a finding reflected in previous trials where intravenous iron supplementation stayed essential during HIF-PHI treatment [17].

In terms of safety, mortality differences among agents were not statistically significant. The odds ratio for death comparing daprodustat to darbepoetin was 0.89 (95% CrI: 0.63 to 1.26), and vadadustat exhibited a shift toward increased mortality (OR: 1.12; 95% CrI: 0.81 to 1.56) [14]. Considerably, these results are consistent with those of the PRO2TECT trial, which demonstrated increased cardiovascular events and mortality with vadadustat in non-dialysis patients when compared to darbepoetin, emphasizing caution in its use [18]. In contrast, roxadustat had a comparatively favorable profile in dialysis-dependent patients, especially in Asian cohorts, as seen in pooled analyses from China and Japan [19, 20].

Cardiovascular safety is a key determinant in CKD populations. While daprodustat highlighted a neutral cardiovascular profile compared to darbepoetin (OR: 0.92; 95% CrI: 0.65 to 1.28), vadadustat demonstrated an elevated risk pattern (OR: 1.35; 95% CrI: 0.94 to 1.95) [14, 18]. However, Roxadustat ranked lower for cardiovascular safety but showed favorable effects in dialysis-dependent patients, highlighting its heterogeneity based on population and suggesting the need for individualized agent selection rather than broad generalization [14, 20].

Hypertension, a well-established ESA-related complication, was consistently more frequent with darbepoetin and other traditional ESAs compared to HIF-PHIs. Daprodustat and roxadustat showed reduced hypertension rates (daprodustat OR: 0.79; 95% CrI: 0.59 to 1.04), supporting the hypothesis that HIF-PHIs maintain more physiological erythropoietin levels, avoiding supraphysiologic peaks seen with ESAs [21].

Gastrointestinal adverse effects were more prominent with roxadustat (OR: 1.88; 95% CrI: 1.10 to 3.22), particularly diarrhea. These symptoms, though generally non-severe, can affect adherence and may vary regionally due to dietary or microbiome factors [16, 21]. In contrast, daprodustat and vadadustat showed a relatively favorable gastrointestinal safety profile.

Thrombotic complications and diabetes-related adverse events were unique and demonstrated no notable differences between treatment groups. Prior analyses have likewise reported no major thrombotic signal with HIF-PHIs, despite the exact event rates being low and wide CrIs decrease certainty [17, 19].

However, the clinical transition of these findings should consider drug availability, regulations, and cost. Darbepoetin (ESAs) have been proven to be reimbursed and globally available and used, but the standardization of roxadustat and daprodustat is limited to some regions, including Japan, China, and in certain western markets, having high cost and restricted formulary access in the majority of healthcare settings, confining its real-world applicability despite its good clinical profiles [22, 23].

Regardless of these positive trends for HIF-PHIs, the intersecting credible intervals in almost all comparisons suggest persisting uncertainty. Furthermore, differences in trial populations (dialysis vs. non-dialysis), geography, dosing of the medications, and outcome definitions bring up heterogeneity that restricts direct comparability. Subgroup-specific variabilities such as the cardiovascular benefit of roxadustat in dialysis patients but potential harm from vadadustat in non-dialysis settings highlight the requirement for precision medicine strategies and agent-specific recommendations.

### Clinical implications

This analysis strengthens the use of daprodustat and roxadustat as effective and possibly safer options for ESAs in properly chosen CKD patients. Their oral administration also provides practical benefits, especially in non-dialysis patients where injectable therapies are less practicable. However, given agent-specific variations, clinical decisions should take into consideration the patient’s comorbidities, stage of CKD, local drug accessibility, and cost-effectiveness of the drug.

## Limitations

This systematic review and network meta-analysis have some important limitations that should be acknowledged. First, most of the included trials had comparatively short follow-up times, restricting the evaluation of long-term safety outcomes such as prolonged cardiovascular effects, malignancy risk, or longevity of hemoglobin response. Second, heterogeneity in adverse event findings across studies such as variability in definitions, severity grading, and reporting criteria may have influenced the validity and comparability of risk estimates. Third, the analysis does not account for real-world considerations such as regulatory approval status, drug accessibility, and cost-effectiveness affecting the clinical translation. Fourth, most of the included trials were sponsored by industry, which gives rise to a potential risk of selective outcome reporting bias and publication bias. Fifth, detailed subgroup data, including categorization by dialysis status, geographic region, and dosing strategies, were not consistently reported or were not available, limiting the potential to perform more granular analyses. Finally, while the Bayesian network meta-analysis framework allows for thorough indirect comparisons, assumptions of transitivity and consistency could not be fully assessed due to variability in study populations, designs, and comparator interventions among the included trials. These limitations suggest cautious interpretation of both trial-level and real-world constraints while informing future research and clinical decision-making in CKD-related anemia.

## Future directions

Future direct head to head RCTs with longer follow-up time and real-world registry studies are necessary to validate these findings. Studies should focus on comparative effectiveness across CKD stages, investigate long-term cardiovascular and renal outcomes, and evaluate cost-effectiveness in both dialysis and non-dialysis settings. Pharmacogenomic research may also help identify patient populations most likely to benefit from specific HIF-PHIs.

## Conclusion

This systematic network meta-analysis demonstrates that HIF prolyl hydroxylase inhibitors, especially daprodustat and roxadustat, provide equivalent or modestly improved hemoglobin effectiveness over darbepoetin, with varied safety profiles. Daprodustat demonstrated a balanced profile with potential cardiovascular and hemodynamic benefits, while roxadustat was associated with higher gastrointestinal adverse events. Although conventional ESAs like darbepoetin are still effective, the oral route of administration and potential safety advantages of HIF-PHIs may serve as a promising option in properly selected CKD patients. However, heterogeneity across trials, intersecting credible intervals, and limited regional approval and availability of HIF-PHIs warrant cautious interpretation of findings. Future large-scale, long-term head-to-head trials and real-world data are essential to validate these findings, choose optimal candidates for each therapy, and inform evidence-based, personalized anemia management in CKD.

## Electronic supplementary material

Below is the link to the electronic supplementary material.

Supplementary Material 1

## Data Availability

The datasets generated and/or analyzed during the current study are available within the manuscript and supplementary file.

## Abbreviations

CKD: Chronic kidney disease
ESAs: Erythropoietin-stimulating agents
RoB: Risk of bias assessment
DPO: Darbepoetin alfa
PRISMA: Preferred Reporting Items for Systematic Reviews and Meta-analysis
PRISMA-NMA: Preferred Reporting Items for Systematic Reviews and Meta-analysis - network meta-analysis
MeSH: Medical Subject Headings
RCTs: Randomized Controlled Trials:
GFR: Glomerular Filtration Rate
OR: Odds ratio
MD: Mean Difference
SUCRA: Surface under the cumulative ranking
CERA: Continuous Erythropoietin Receptor Activator
PEG-epoetin beta: Methoxy polyethylene glycol-epoetin beta
rHuEPO: Recombinant human erythropoietin
HIF-PHIs: Hypoxia-inducible factor prolyl hydroxylase inhibitors
Hb: Hemoglobin
