# Supplementary figures and images for: Comparative effectiveness of darbepoetin vs other agents in chronic kidney disease-related anemia: a systematic review and network meta-analysis
Source: BMC Nephrol. 2025 Nov 17;26:641. doi: 10.1186/s12882-025-04557-7 (PMC12625550; doi:10.1186/s12882-025-04557-7)

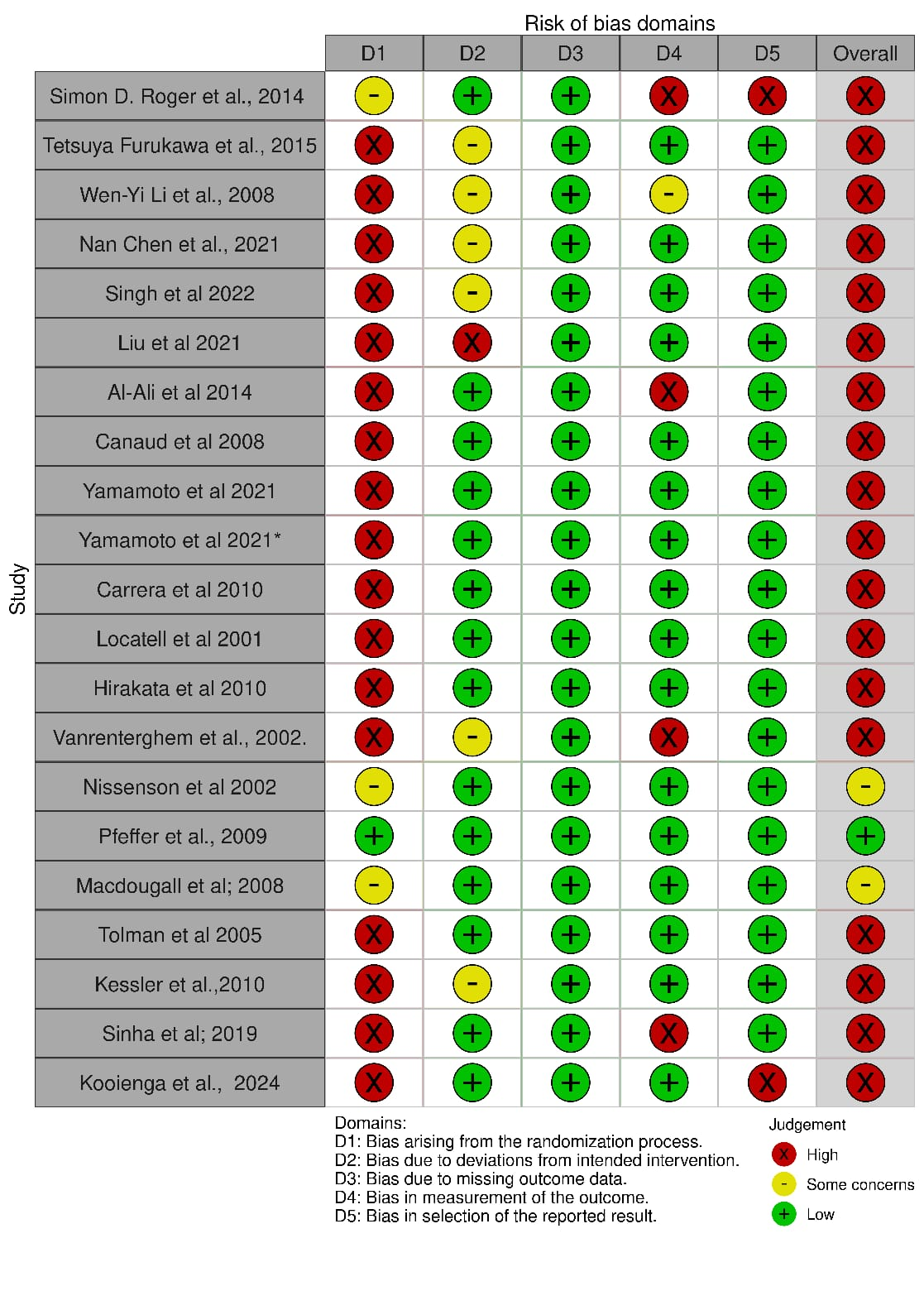


**Figure S1: Assessment of Risk of Bias**


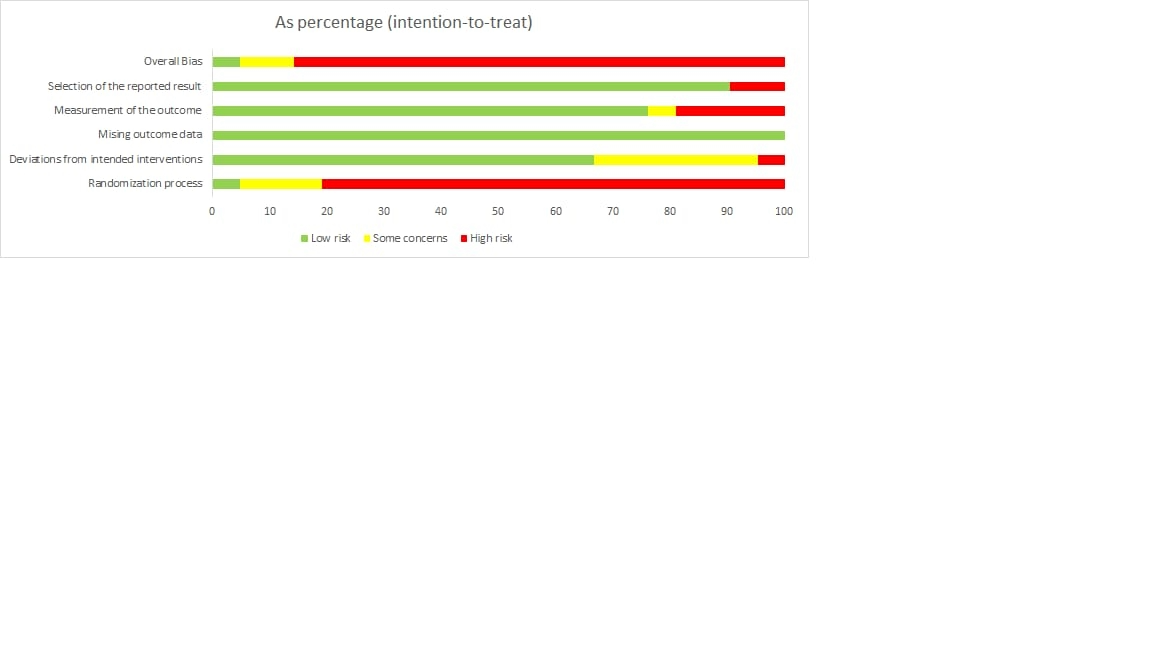
**Figure S2: Risk of Bias Graph**

Supplement: Supplementary file 1 — Supplementary Material 1 [file 12882_2025_4557_MOESM1_ESM.docx]
